# A high‐affinity, bivalent PDZ domain inhibitor complexes PICK1 to alleviate neuropathic pain

**DOI:** 10.15252/emmm.201911248

**Published:** 2020-04-30

**Authors:** Nikolaj R Christensen, Marta De Luca, Michael B Lever, Mette Richner, Astrid B Hansen, Gith Noes‐Holt, Kathrine L Jensen, Mette Rathje, Dennis Bo Jensen, Simon Erlendsson, Christian RO Bartling, Ina Ammendrup‐Johnsen, Sofie E Pedersen, Michèle Schönauer, Klaus B Nissen, Søren R Midtgaard, Kaare Teilum, Lise Arleth, Andreas T Sørensen, Anders Bach, Kristian Strømgaard, Claire F Meehan, Christian B Vægter, Ulrik Gether, Kenneth L Madsen

**Affiliations:** ^1^ Molecular Neuropharmacology and Genetics Laboratory Department of Neuroscience Faculty of Health and Medical Sciences University of Copenhagen Copenhagen Denmark; ^2^ Center for Biopharmaceuticals Department of Drug Design and Pharmacology Faculty of Health and Medicine University of Copenhagen Copenhagen Denmark; ^3^ Danish Research Institute of Translational Neuroscience (DANDRITE) Nordic‐EMBL Partnership for Molecular Medicine Department of Biomedicine Aarhus University Aarhus C Denmark; ^4^ Department of Neuroscience Faculty of Health and Medical Sciences University of Copenhagen Copenhagen Denmark; ^5^ Structural biology and NMR Laboratory Department of Biology University of Copenhagen Copenhagen Denmark; ^6^ Structural Biophysics Niels Bohr Institute University of Copenhagen Copenhagen Denmark

**Keywords:** biopharmaceuticals, calcium permeable AMPARs, maladaptive plasticity, scaffold proteins, synaptic plasticity, Neuroscience, Chemical Biology

## Abstract

Maladaptive plasticity involving increased expression of AMPA‐type glutamate receptors is involved in several pathologies, including neuropathic pain, but direct inhibition of AMPARs is associated with side effects. As an alternative, we developed a cell‐permeable, high‐affinity (~2 nM) peptide inhibitor, Tat‐P_4_‐(C5)_2_, of the PDZ domain protein PICK1 to interfere with increased AMPAR expression. The affinity is obtained partly from the Tat peptide and partly from the bivalency of the PDZ motif, engaging PDZ domains from two separate PICK1 dimers to form a tetrameric complex. Bivalent Tat‐P_4_‐(C5)_2_ disrupts PICK1 interaction with membrane proteins on supported cell membrane sheets and reduce the interaction of AMPARs with PICK1 and AMPA‐receptor surface expression *in vivo*. Moreover, Tat‐P_4_‐(C5)_2_ administration reduces spinal cord transmission and alleviates mechanical hyperalgesia in the spared nerve injury model of neuropathic pain. Taken together, our data reveal Tat‐P_4_‐(C5)_2_ as a novel promising lead for neuropathic pain treatment and expand the therapeutic potential of bivalent inhibitors to non‐tandem protein–protein interaction domains.

## Graphical abstract

Neuropathic pain is characterized by hypersensitivity to temperature and touch as well as spontaneous outburst of pain. This study identifies a peptide that can inhibit PICK1 and thereby interfere with insertion of excess glutamate receptor underlying the hypersensitivity in neuropathic pain.

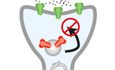

**The paper explained**

**Problem**

Neuropathic pain affects 7–10% of the world population with higher prevalence in women and elderly. Current medication, including anti‐epileptics, antidepressants, and opioids, provides only partial pain relief and comes with considerable side effects. Consequently, there is an urgent need for better treatment. The causes for development of neuropathic pain include diabetes mellitus, treatment with chemotherapeutics, and herpes zoster, which all cause damage to the peripheral nervous system leading to increased central pain perception. This central sensitization involves insertion of excess glutamate receptors in the dorsal horn synapse, and direct modulation of the glutamatergic transmission by glutamate receptor antagonist to obtain better efficacy has been pursued, but clinical development has been discontinued due to adverse side effects.

**Results**

This paper demonstrates a refinement of this approach by targeting the insertion of excess glutamate receptors in the hyper‐sensitized condition rather than blocking the receptors themselves. This was achieved by development of a peptide that can inhibit the scaffold protein PICK1, which has previously been evoked as a molecular target in pain treatment because it sustains insertion of a subtype of glutamate receptors. Injection of the peptide in the spinal cord effectively relief pain in an animal model of neuropathy, whereas injection in the paw of the injured hind limb was ineffective, suggesting a central mechanism of action. This was supported by electrophysiological *in vivo* measurement in injured animals, which confirmed that the peptide reduced spinal cord transmission.

**Impact**

The approach presented here evokes a novel mechanism of action for pain relief and potentially provides a potent tool for interfering with excessive glutamate receptor transmission in pain. Importantly, since the peptide only affect disease‐related receptor insertion, we do not anticipate major impact on basal neurotransmission. Consequently, with the proper formulation to enable appropriate administration, the peptide is a strong candidate for extended pre‐clinical studies leading to future clinical trials for neuropathic pain following peripheral nerve damage.

## Introduction

Excitatory communication between neurons in the central nervous system relies almost exclusively on glutamatergic neurotransmission. Concordantly, most neurological and psychiatric diseases, including devastating conditions such as neuropathic pain, feature distinct glutamatergic components, yet very few drugs targeting glutamate neurotransmission have been approved for clinical applications. Indeed, major efforts have been directed toward developing compounds targeting the ionotropic NMDA (N‐methyl‐D‐aspartate) and AMPA (α‐amino‐3‐hydroxy‐5‐methyl‐4‐isoxazolepropionic acid)‐type glutamate receptors, but most such compounds have failed during clinical development due to lack of efficacy or as a result of unacceptable side effects (Tymianski, 2014).

An attractive alternative approach to manipulate glutamatergic neurotransmission is to target the synaptic scaffold proteins that orchestrate synaptic signaling complexes and dynamically regulate the surface expression and ion conductance of the ionotropic glutamate receptors in the postsynaptic density. Several of these proteins contain PDZ (PSD‐95/Discs‐large/ZO‐1 homology) domains that are characterized by an elongated binding crevice, which binds the extreme C‐terminus of interaction partners, including in several cases the ionotropic glutamate receptors themselves (Khan & Lafon, 2014). Despite the well‐defined binding crevice, it has proven challenging to develop sufficiently potent small‐molecule inhibitors of PDZ domain‐mediated interactions as for protein–protein interactions (PPIs) in general (Laraia *et al*, 2015). Also, given the relatively low micromolar affinity of the interacting C‐terminal peptides, it has likewise been difficult to develop efficacious peptide‐based ligands. An exception is a membrane‐permeable peptide ligand targeting the postsynaptic scaffold protein PSD95 (Aarts *et al*, 2002), which recently passed phase 3 clinical trial for the treatment of ischemia after stroke (Hill *et al*, 2020). By generating a bivalent peptide ligand that simultaneously target two adjacent, “tandem” PDZ domains in PSD‐95, it has been possible to dramatically enhance efficacy affinity/avidity (low nanomolar) and plasma stability (hours) (Sainlos *et al*, 2011; Bach *et al*, 2012).

PICK1 (protein interacting with C kinase) is another PDZ domain containing scaffold protein found in glutamatergic neurons (Hanley, 2008). PICK1 plays a central role in synaptic plasticity (Volk *et al*, 2010) and is a functional dimer with two PDZ domains flanking the central membrane binding BAR (Bin/amphiphysin/Rvs) domain, which also mediates the dimerization (Karlsen *et al*, 2015). The PICK1 PDZ domain interacts directly with the C‐terminus of a number of different membrane proteins and kinases (Staudinger *et al*, 1997), including the GluA2 subunit of the AMPA receptors (AMPARs) (Dev *et al*, 1999; Xia *et al*, 1999). Functionally, PICK1 has been shown to regulate protein kinase C (PKC)‐dependent phosphorylation of serine 880 (S880) of the AMPAR GluA2 subunit (Lu & Ziff, 2005), important for AMPAR trafficking during synaptic plasticity (Kim *et al*, 2001; Chung *et al*, 2003; Seidenman *et al*, 2003; Steinberg *et al*, 2006). Recent studies have further suggested a more direct role of PICK1 in regulating AMPAR surface stabilization, internalization, and recycling (Gardner *et al*, 2005; Jin *et al*, 2006; Cao *et al*, 2007; Rocca *et al*, 2008; Citri *et al*, 2010; Fiuza *et al*, 2017). In this context, expression of calcium permeable (CP) AMPARs in the process of homeostatic scaling (Clem *et al*, 2010) as well as in maladaptive plasticity associated with cocaine addiction (Wolf & Ferrario, 2010; Luscher & Malenka, 2011) and oxygen–glucose depletion (Dixon *et al*, 2009) has been shown to involve PICK1.

Finally, based on studies in animal models using inhibitory peptides, siRNA, and knock‐out mice, PICK1 has been shown to be implicated in central sensitization of neuropathic pain (Garry *et al*, 2003; Atianjoh *et al*, 2010; Wang *et al*, 2011, 2016). This suggests PICK1 as a potential novel target for pharmaceutical intervention in chronic pain conditions where blockade of its PDZ domain can restrain unwanted sensitization by impairing glutamatergic AMPAR signaling (Garry *et al*, 2003; Atianjoh *et al*, 2010; Wang *et al*, 2011, 2016). Nonetheless, development of an efficacious inhibitor of the PICK1 PDZ has so far failed with the best inhibitors having affinities in the sub‐micromolar to micromolar range (Garry *et al*, 2003; Bach *et al*, 2010; Thorsen *et al*, 2010; Marcotte *et al*, 2018).

Here, we present the development of a highly potent (1.7 nM) and efficacious peptide inhibitor of PICK1 for putative neuropathic pain treatment. Although the PICK1 PDZ domains in the functional PICK1 dimer are presumably > 150 Å apart (Erlendsson *et al*, 2015; Karlsen *et al*, 2015), we demonstrate that a bivalent peptide ligand displays a striking ~1,000‐fold increase in affinity over analogous monomeric peptides. Size‐exclusion chromatography and small angle X‐ray scattering (SAXS) demonstrate that the bivalent peptide obtains avidity in a highly unique manner from the assembly of a novel complex of PICK1 dimers‐of‐dimers. We further show that the Tat‐conjugated bivalent peptide is membrane permeable, engages with the target protein, and interferes with the PICK1‐dependent phosphorylation of the GluA2 subunit of the AMPARs. Furthermore, the bivalent, but not the corresponding monovalent, high‐affinity peptide can actively disrupt already established PICK1‐receptor complexes on supported cell membrane sheets, as well as interfere with PICK1‐GluA2 co‐immunoprecipitation and facilitate constitutive internalization of GluA2. Finally, we show that administration of the bivalent high‐affinity peptide reduces spinal cord transmission and alleviates mechanical allodynia for up to 4 h in both the acute and chronic phase of the mouse spared nerve injury (SNI) model of neuropathic pain. Taken together, our results demonstrate that bivalent ligands represent a strong means to achieve high, pharmacologically relevant affinity even when targeting protein–protein interaction domains that only are adjacent to one another in transient oligomeric configurations of the target protein.

## Results

### Design and development of a high‐affinity bivalent inhibitor of PICK1

**Figure EV1:**
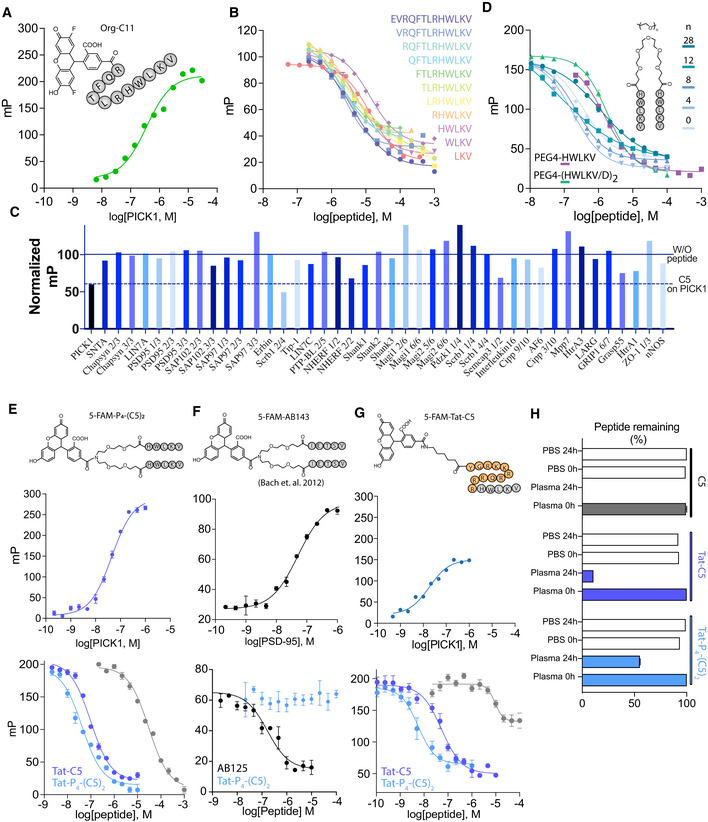
Development of Tat‐C5 and Tat‐P_4_‐(C5)_2_ AFluorescence polarization saturation binding using Org‐C11 (20 nM) against an increasing concentration of PICK1.BEffects of truncation on competitive binding of DAT C‐terminal peptides to PICK1 using Org‐C11 (20 nM) as reporter.CSelectivity screen for Tat‐C5 against a selection of 42 purified PDZ domains, performed with a fixed concentration of C5 (10 μM) in competition with PDZ domains and their respective fluorescent ligands. Data are normalized to binding in absence of peptide (full line). Dashed line represents the level of competition obtained for PICK1.DCompetitive binding curves of bivalent C5 PEG_n_ variants. With PEG linker, length indicated.ESaturation (top) and competition binding curves using 5‐FAM‐PEG_4_‐(C5)_2_ (5 nM) and PICK1 against C5, Tat‐C5, and Tat‐P_4_‐(C5)_2._
FSaturation (top) and competition binding curves using 5‐FAM‐AB143 (5 nM) and PSD‐95 against AB125 (positive control), and Tat‐P_4_‐(C5)_2_.GSaturation (top) and competition binding curves using 5‐FAM‐Tat‐C5 (2 nM) and PICK1 against C5, TatC5, and Tat‐P_4_‐(C5)_2._
HPlasma stability as assessed by incubation of peptides as indicated with human plasma for 24 h followed by quantification by UPLC.Data information: Data are shown as mean with error bars as SEM of *n* ≥ 3. Binding curves were fitted to a log dose response (three parameters) extracting the K_d_ or IC_50_ using GraphPad Prism 8.3. *K*_i_ was calculated using the Cheng–Prusoff equation.

**Figure 1:**
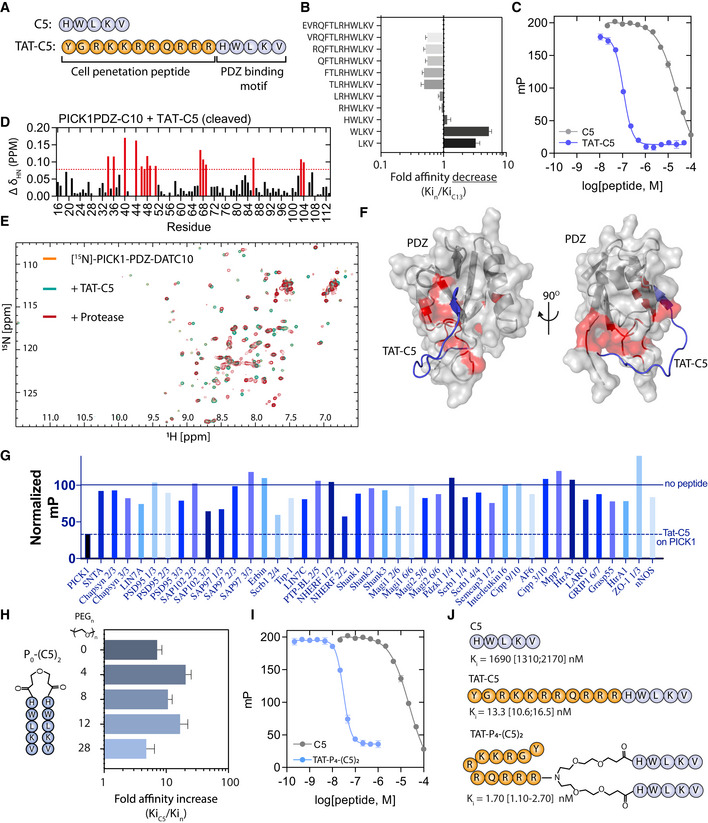
Development of a high‐affinity bivalent inhibitor of PICK1s APrimary sequence of 5‐mer peptide (C5) derived from the dopamine transporter C‐terminus and the Tat‐conjugated variant, Tat‐C5.BFluorescence polarization (FP) competition derived affinity assessment of consecutive truncations of the DAT C13 C‐terminus as indicated by the sequences. Bars indicate the mean fold change relative to C13 (*K*_i_ = 1.69 μM). Data are shown as mean with error bars as SEM with *n* = 6. Representative binding curves are shown in Fig EV1A.CFP competition binding of C5 and Tat‐C5 to PICK1 with 5FAM‐Tat‐C5 (20 nM) as tracer. Saturation binding curve is shown in Fig EV1B.DBackbone amide chemical shift changes (Δδ_HN_) of PICK1‐PDZ‐C10 after addition of Tat‐C5 and addition of protease to cleave C10 from the PDZ domain and allow exchange. Numbers refer to residue number in PICK1 (UniProt: Q9EP80). Red bars indicate a chemical shift larger than mean+SD.E
^1^H‐^15^N‐HSQC 2D spectra of PICK1‐PDZ‐C10 (orange) following addition of Tat‐C5 (green) and subsequent addition of protease (red) to allow for exchange between C10 and Tat‐C5.FDocking model, for visual purposes, of PICK1‐PDZ (PDB: 2LUI) in complex with Tat‐C5 (blue), with perturbed residues indicated from (D) in red.GSelectivity screen for Tat‐C5 against a selection of 42 purified PDZ domains performed with a fixed concentration of Tat‐C5 (10 μM) in competition with PDZ domains and their respective fluorescent ligands. Data are normalized to binding in absence of peptide (full line). Dashed line represents the level of competition obtained for PICK1. Screen for C5 can be seen in Fig EV1C.HAffinity gain of bivalent C5 peptides (P_n_‐(C5)_2_) N‐terminally conjugated with different length PEG linkers as indicated. Bars indicate the mean fold change relative to C5 (*K*_i_ = 1.69 μM). Data are shown as mean with error bars as SEM with *n* = 6. Representative binding curves are shown in Fig EV1C.IFP competition binding of C5 (also used in C) and Tat‐P_4_‐(C5)_2_ to PICK1 with 5FAM‐Tat‐C5 (20 nM) as tracer.JStructure and highest obtained affinity of C5, Tat11‐C5, and Tat11‐P_4_‐(C5)_2_ toward PICK1.Data information: Data points and bars are shown as mean with error bars as SEM of *n* ≥ 3. Docking in (F) was done using HADDOCK (van Zundert *et al*, 2016), with residues indicated from (D) as essential residues for interaction, but no further restraints, therefore being only for visual purposes.

Starting from the best‐known binder of the PICK1 PDZ domain, DAT C13 (corresponding to the C‐terminal 13 residues of the dopamine transporter (DAT)) (*K*_*i*_ = 1 μM) (Madsen *et al*, 2005), we sought to identify the shortest peptide sequence with conserved affinity toward the PICK1 PDZ domain. To this end, we took advantage of a fluorescence polarization (FP) competition assay using a fluorescent tracer peptide corresponding to the C‐terminal 11 residues of the DAT (OrG‐C11) (Fig EV1A). The DAT C13 (C13) competitor peptide was successively truncated from the N‐terminus and retained affinity, down to DAT‐C5 (C5), while further truncation (C4 and C3) slightly reduced affinity (Figs 1A and B, and EV1B). We evaluated the specificity of C5 (Fig EV1C) for a broad selection of PDZ domains (both class I and II) that were previously purified and had known ligands (Stiffler *et al*, 2007). C5 (10 μM) (black column) competed for binding to PICK1 as seen by the reduction in normalized mP (milli‐polarization) compared to no peptide (full line). The C5 peptide primarily bound to the PDZ domain of PICK1, however with notable exceptions for Scribble (Scrb1) PDZ 2/4, Na(+)/H(+) exchange regulatory cofactor NHE‐RF1 (NHERF) PDZ 2/2, and E3 ubiquitin‐protein ligase PDZRN3 (Semcap3) PDZ 1/2, which were inhibited to similar level as for PICK1 (indicated by dashed line) (Fig EV1C). C5, however, did not compete with binding of either domain in full binding curves, suggesting they were false positive (Appendix Fig S1).

N‐terminal modification of the C3‐C5 peptides, similar to Bach *et al* (2011), did not significantly affect affinity (Appendix Table S1). To render the peptide cell permeable, we combined C5 with the 11‐amino‐acid cell penetration peptide from human immunodeficiency virus 1 (HIV‐1) and trans‐activator of transcription protein (Tat) (Richard *et al*, 2003; Lee *et al*, 2014) (YGRKKRRQRRR‐HWLKV, termed Tat‐C5). To be able to do binding studies, we also produced a fluorescent variant (5FAM‐Tat‐C5, Fig EV1E). Surprisingly, the addition of the Tat peptide gave rise to a dramatic increase in affinity of Tat‐C5 for PICK1 (*K*_i_ = 18.3 nM), as assessed in competition with 5FAM‐Tat‐C5 (20 nM) (Fig 1C).

To explore the structural basis for the increased affinity, we took advantage of the PICK1 PDZ‐DAT C10 construct (Erlendsson *et al*, 2014). Upon displacement of the DAT C10 (triggered by protease cleavage of the linker connecting the PDZ domain to the DAT C10 sequence) with equimolar amounts of the Tat‐C5 peptide, we found that residues in the actual binding groove I33, I35, and I37 in βB and L83, A87, and I90 in αB were mostly unaffected and that canonical PDZ binding is therefore not compromised by conjugation of the Tat sequence. Instead, we observed strong chemical shift changes for several residues (G40, C44, C46, L47, Y48, and I49) in the Cys‐loop, connecting βB and βC, as well as, a negatively charged patch constituted by residues D68 and E69, opposite the canonical PDZ domain binding groove (Fig 1D and E). Docking the Tat‐C5 (blue) using the observed chemical shifts as restraints (van Zundert *et al*, 2016) confirmed that canonical binding of the C5 residues allows for the electropositive Tat residues to wrap around the PDZ domain and simultaneously interact with D68 and E69 (Fig 1F). The specificity of Tat‐C5 for the PICK1 PDZ domain increases when compared to the C5 peptide alone (Figs 1G and EV1C), which suggests that the exact spatial positioning of negatively charged residues and interaction with and/or flexibility of the Cys‐loop are important for both affinity and specificity.

Based on our previous work (Bach *et al*, 2012), we next hypothesized that the native dimeric structure of PICK1 could be targeted using a bivalent ligand with two binding peptides connected via a polyethylene glycol (PEG) linker. We therefore designed a range of bivalent peptides of DATC5, which were fused at the N‐terminus with PEG linkers of different lengths, PEG_n_‐(C5)_2_ (Figs 1H and EV1D). All bivalent peptides showed > 10‐fold increased affinity with an optimal linker length determined to be 4 PEG units, P_4_‐(C5)_2_, spanning ~43 Å (*K*_i_ = 98 nM) and giving rise to a 15‐fold affinity increase compared to monovalent C5 (*K*_i_ = 1.42 μM) (Figs 1G and EV1C). This was somewhat surprising given the average distance between the PDZ domains of the PICK1 dimer is estimated to be ~180 Å (Karlsen *et al*, 2015). The dimeric ligand with the longest linker (P_28_‐(C5)_2_), which spans ~130 Å, on the other hand, showed the lowest affinity (*K*_i_ = 593 nM), presumably due to increased entropic penalty (Figs 1H and EV1D). To render the bivalent peptide cell membrane permeable, we modified the PEG4 linker to enable conjugation to Tat (Bach *et al*, 2012), which again increased the affinity considerably (6.2 nM), as assessed in competition with 5FAM‐Tat‐C5 (20 nM) (Fig 1I). The resulting peptide Tat‐PEG_4_‐(DATC5)_2_, termed Tat‐P_4_‐(C5)_2_, was labeled with tetramethylrhodamine (TMR) (TMR‐Tat‐P_4_‐(C5)_2_), and saturation binding curves also demonstrated low nanomolar affinity (Appendix Fig S2). Finally, no binding of Tat‐P_4_‐(C5)_2_ was observed to another bivalent target, PSD95, whereas an analogous dimeric inhibitor with a PSD95 specific sequence showed potent binding (Bach *et al*, 2009) (Fig EV1F).

Finally, using low concentration (2 nM) of 5FAM‐Tat‐C5 as optimal tracer (highest affinity) (Huang, 2003), we obtained final affinities for Tat‐C5 (*K*_i_ = 13.3 nM/125‐fold increase over C5) and Tat‐P_4_‐(C5)_2_ (*K*_i_ = 1.7 nM/994‐fold increase over C5) (Figs 1J and EV1G), respectively, ranking Tat‐P_4_‐(C5)_2_ among the most potent PDZ domain inhibitors. This affinity gain was ~15‐fold from the bivalency and additional ~50‐fold from the Tat sequence. Importantly, the peptides also potently competed with DAT and GluA2 tracers although this was challenging to assess due to inhibitor depletion in the assay (Appendix Fig S3).

Previous studies on the analogous PSD95 peptides have demonstrated that Tat conjugation as well as the PEG linker does not merely increase binding strength but also increases plasma stability, which is crucial for *in vivo* administration. Similarly, we observed that whereas the C5 peptide was completely degraded, ~5% of Tat‐C5 and more than 50% of Tat‐P_4_‐(C5)_2_ endured incubation with human plasma for 24 h (Fig EV1H), making these peptides relevant for *in vivo* application.

### Tat‐P_4_‐(C5)_2_ dissociates PICK1 from membrane‐embedded receptors

**Figure 2:**
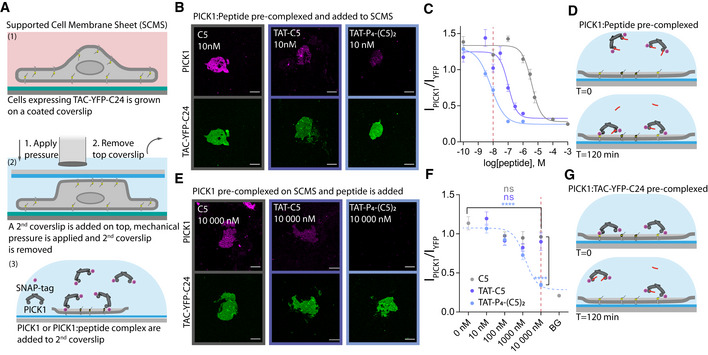
High‐affinity PDZ inhibitors can dissociate PICK1 from transmembrane interaction partner AIllustration of supported cell membrane sheet (SCMS) assay.BRepresentative confocal images of SCMS expressing Tac‐YFP‐DATC24 incubated with pre‐complexed fluorescently labeled PICK1 and unlabeled peptides as indicated (10 nM), scale bar 10 μm. Full concentration curves shown in Appendix Fig S4 and include the representative images shown here.CSCMS derived competitive binding curves for peptides as indicated, red line indicates 10 nM as shown in representative images.DExperimental outline of pre‐incubation with peptide.ERepresentative confocal images of SCMS expressing Tac‐YFP‐DATC24, incubated with fluorescently labeled PICK1 and subsequently unlabeled peptide as indicated (10 μM). Scale bar 10 μm. Full concentration curves shown in Fig EV2 and include the representative images shown here.FEfficacy of the peptides as indicated to facilitate dissociation of PICK1 from SCMS, red line indicates 10 μM as shown in representative images.GExperimental outline post‐incubation with peptide.Data information: Data points are shown as mean with error bars as SEM of *n* ≥ 14. Curves were fitted to a log dose response (three parameters) extracting the IC_50_ using GraphPad Prism 7.0. Data points in (F) were compared at different concentrations using two‐way ANOVA followed by Tukey's multiple comparison test (****P <* *0.0001).

**Figure EV2:**
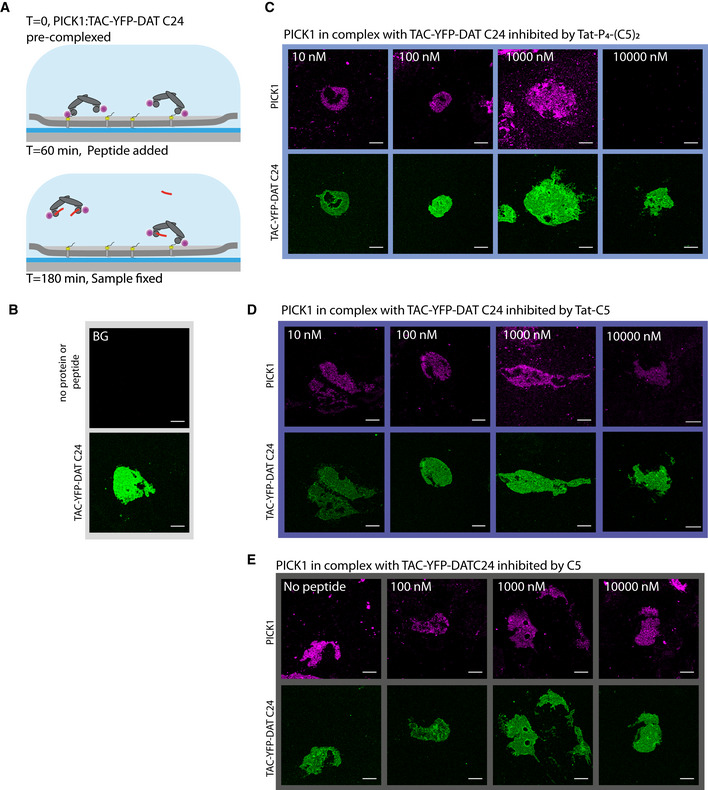
Dissociation of PICK1 binding from SCMSs in presence of inhibitory peptides AIllustration of experimental approach. Fluorescently labeled PICK1 was allowed to interact with the supported cell membrane sheet (SCMS) expressing TAC‐YFP‐DAT C24 prior to incubation with peptide for 1 h.B–ERepresentative fluorescence confocal images of SCMS expressing TAC‐YFP‐DAT C24 (green) incubated with fluorescently labeled PICK1 (magenta) and subsequently incubated with unlabeled peptide at indicated concentrations. Visible reduction in binding of PICK1 (magenta) is observed at 10 μM of Tat‐P_4_‐(C5)_2_, but not for Tat‐C5 and C5, suggesting that Tat‐P_4_‐(C5)_2_ can facilitate dissociation of PICK1 from SCMSs. For curves in Fig 1F, images were pooled from three independent experiments. Note that images with 10,000 nM peptide are the same as used in Fig 2E.Data information: In all images, scale bars represent 10 μm.

PICK1 serves its functional role as a scaffold protein interacting via its PDZ domain with receptors, transporters, and ion channels embedded in the cell membrane. To determine the efficacy of Tat‐P_4_‐(C5)_2_ and Tat‐C5 to interfere with PICK1 binding to membrane‐embedded proteins, we took advantage of the supported cell membrane sheet (SCMS) approach (Erlendsson *et al*, 2019) (Fig 2A). Using this approach, we recently demonstrated that PICK1 interacts with membrane‐embedded Tac‐YFP‐DAT C24 (the single transmembrane IL2 subunit fused to YFP and the 24 C‐terminal residues of DAT) with a binding strength of ~50 nM (Erlendsson *et al*, 2019). First, we pre‐incubated fluorescently labeled recombinant rat PICK1 (300 nM SNAP543‐PICK1) with increasing concentrations of C5, Tat‐C5, or Tat‐P_4_‐(C5)_2_ to compete for binding to SCMSs expressing Tac‐YFP‐DAT C24 (Fig 2B–D and Appendix Fig S4). The IC_50_ values (C5 = 3.80 μM; Tat‐C5 = 101 nM; Tat‐P_4_‐(C5)_2_ = 7.0 nM) were slightly weaker than the affinities obtained by FP competition binding, but confirmed the dimeric ligand as a superior inhibitor of PICK1. Next, we determined the ability of the peptides to dissociate pre‐bound PICK1 from the SCMS. Following pre‐incubation of fluorescently labeled PICK1 on SCMSs expressing Tac‐YFP‐DAT C24, unbound PICK1 was washed away before incubation with increasing concentrations of C5, Tat‐C5, or Tat‐P_4_‐(C5)_2_ (Fig 2E–G and images supporting the full curves and method in Fig EV2A–E). Strikingly, Tat‐P_4_‐(C5)_2_ increased the macroscopic off‐rate of bivalently bound PICK1 with an apparent IC_50_ = 1.17 μM, whereas neither C5 nor Tat‐C5 significantly dissociated PICK1 from SCMSs. PICK1 pre‐binding to the sheet is likely to give rise to depletion of the inhibitors, which means that we can probably not translate the absolute potency obtained from the experiment to a neuronal setting. The relative difference, however, is striking and suggests a unique advantage of the dimeric ligand in a therapeutic context where the ability to dissociate a preformed complex predictably would be highly advantageous.

### High affinity of Tat‐P_4_‐(C5)_2_ results from complex assembly with tetrameric PICK1

**Figure 3:**
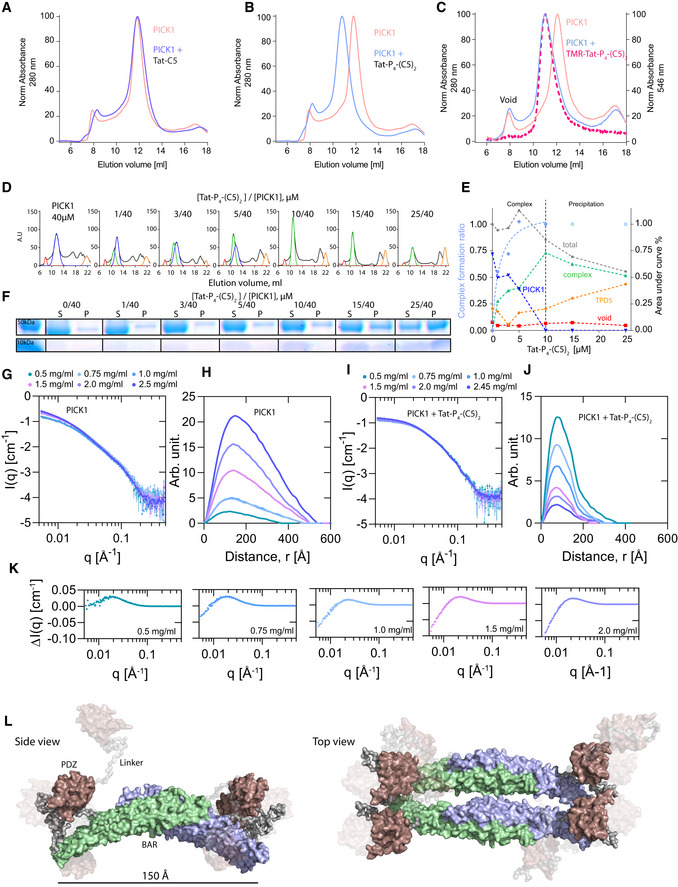
Tat‐P_4_(C5)_2_, but not Tat‐C5, reconfigures PICK1 into a novel tetrameric state ASize‐exclusion chromatography (SEC) profiles of PICK1 in absence (pink, Abs_max_ = 11.7 ml) and presence of Tat‐C5 (purple, Abs_max_ = 11.7 ml).BSEC profiles of PICK1 in absence (pink, Abs_max_ = 11.7 ml) and presence of Tat‐P_4_‐(C5)_2_ (blue, Abs_max_ = 10.7 ml).CSEC profiles of PICK1 in absence (pink, Abs_max_ = 11.7 ml) and presence of TMR‐Tat‐P_4_‐(C5)_2_ (blue (abs_280_ nm), red (abs_544_ nm), Abs_max_ = 10.7 ml).DRepresentative SEC profiles of PICK1 (40 μM) incubated with increasing concentration of Tat‐P_4_‐(C5)_2_, colors indicate Gaussian fits of respective population, (red) aggregates/Void, (blue) dimeric PICK1, (green) oligomeric PICK1, (orange) unbound Tat‐P_4_‐(C5)_2_.EQuantification of abs_280_ at E_vol_ = 11.7 ml (dimer—blue) and E_vol_ = 10.7 ml (tetramer—green) as a function of concentration (0‐10 μM) of Tat‐P_4_‐(C5)_2_. Light blue line indicates tetramer fraction of total PICK1.FSDS–PAGE sedimentation assay showing increased amounts of PICK1 in the pellet (P) fraction with increasing concentrations of Tat‐P_4_‐(C5)_2_. PICK1˜50 kDa, Tat‐P_4_‐(C5)_2_˜10 kDa. S denotes supernatant fraction.GSmall angle X‐ray scattering (SAXS)‐derived scattering curves of a concentration range of PICK1 on absolute scale show concentration‐dependent aggregation.HPair distance distribution function (pddf) of PICK1 in absence of Tat‐P_4_‐(C5)_2_, indicating very large protein complexes present in sample.ISAXS‐derived scattering curves of a concentration range of PICK1 in complex with Tat‐P_4_‐(C5)_2_ on absolute scale show minor concentration‐dependent aggregation.JPair distance distribution function (pddf) of a concentration range of PICK1 in presence of Tat‐P_4_‐(C5)_2_, indicating a smaller complex than for PICK1 in absence of Tat‐P_4_‐(C5)_2_.KScattering difference (ΔI(q)  = I(q)_PICK1+Tat‐P4‐(C5)2_ – I(q)_PICK1_) between PICK1 in presence and absence of Tat‐P_4_‐(C5)_2_ at indicated concentration shows a clear difference in scattering below 0.1 Å^−1^.LProposed EOM ensemble for PICK1, in complex with Tat‐P_4_‐(C5)_2_ (not visible in structure) with shading according to model ensemble percentage (10/20/70%). C‐terminal unstructured regions are removed for clarity.Data information: In (A), all elution profiles were normalized to the lowest and highest main elution peak. In (D), elution profiles were normalized to the lowest and highest points of PICK1 in absence of Tat‐P_4_‐(C5)_2_. In (G and H), the pddf was fitted using BayesApp. EOM fits were done using known structures of PICK1 PDZ domain (PDB: 2LUI) and the previously published model for the dimeric BAR domain, connected by full flexible linkers, N‐ and C‐terminal. Model in (L) was prepared using PyMoL. Source data are available online for this figure.

We recently demonstrated that PICK1 forms elongated oligomers in solution using SAXS. Interestingly, the PDZ domains between the individual dimers were predicted to be in much closer proximity than the two PDZ domains within the dimers (Karlsen *et al*, 2015). We therefore wanted to assess whether the dimeric Tat‐P_4_‐(C5)_2_ peptide potentially could stabilize higher order PICK1 complexes. In analytical size‐exclusion chromatography (SEC), purified PICK1 eluted with a main peak at ~12 ml (Fig 3A, pink), which, according to our previous studies, corresponds to dimeric PICK1 (Karlsen *et al*, 2015; Madasu *et al*, 2015). The SEC profile was unchanged by incubation with Tat‐C5 (Fig 3A). Upon incubation with Tat‐P_4_‐(C5)_2_, however, the main elution peak shifted ~1 ml toward a larger hydrodynamic radius far above the hypothetical increase of 3 or 6 kDa resulting from binding of 1 or 2 bivalent peptides, respectively (Fig 3B, light blue). Incubation of PICK1 with the fluorescently labeled TMR‐Tat‐P_4_‐(C5)_2_ peptide showed overlapping curves of the 280 nm absorbance and the 546 nm fluorescence, confirming that the left‐shifted peak indeed contained both PICK1 and the peptide, whereas the void peak (8 ml) showed no peptide binding (Fig 3C). To address the stoichiometry of the complex, we incubated a fixed amount of PICK1 (40 μM) with increasing concentrations of Tat‐P_4_‐(C5)_2_ (Fig 3D and E) and found that, at a molar ratio of 1:4 (Tat‐P_4_‐(C5)_2_:PICK1), all dimeric PICK1 (blue line) was shifted into the complexed state (green line), indicating that complex formation involves 1 bivalent peptide and two PICK1 dimers. At peptide to protein ratios above 1:4, the complex started to precipitate and could be pelleted by centrifugation (Fig 3F). To assess whether the ability of Tat‐P_4_‐(C5)_2_ to form higher order PICK1 complexes was dependent on the dimeric assembly of the BAR domain, PICK1 was dissociated into a monomeric state using detergent (0.1% Triton X‐100) (Karlsen *et al*, 2015). Monomeric PICK1 eluted at 12.7 ml, and interestingly, a 1 ml shift was still observed upon incubation with Tat‐P_4_‐(C5)_2_. Moreover, Tat‐P_4_‐(C5)_2_ still maintained an affinity gain in FP binding of > 300‐fold compared to C5, demonstrating that complex formation is independent of the dimeric BAR domain (Appendix Fig S5 and Appendix Table S2).

**Figure EV3:**
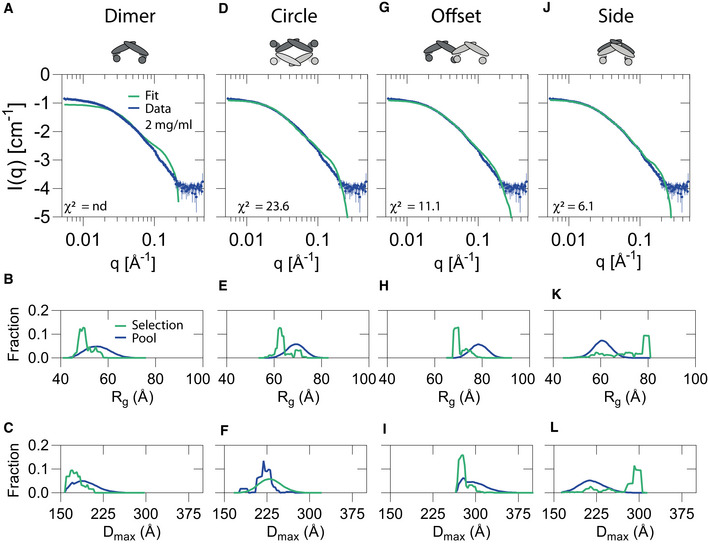
Ensemble optimization method fit of putative tertiary PICK1 configurations to the SAXS data A–LEnsemble optimization method (EOM) fit (green) to scattering data from Fig 3H of PICK1 in complex with Tat‐P_4_‐(C5)_2_ (2 mg/ml) (blue) using a pool (10,000 structures) with R_g_ and D_max_ distribution shown below for (A‐C) dimeric PICK1, (D‐F) a circularly configuration of tetrameric PICK1, (G‐I) an offset configuration of tetrameric PICK1, and (J‐L) a side‐by‐side circularly configuration of tetrameric PICK1. The side‐by‐side configuration provided the best fit as indicated by the lowest χ^2^‐value.

To elucidate the number of PICK1 subunits in the complex stabilized by Tat‐P_4_‐(C5)_2_ and to further investigate its structure, we used SAXS. Data were collected for a concentration range of 0.5–2.5 mg/ml for PICK1 both in absence and presence of Tat‐P_4_‐(C5)_2_ (Fig 3G–J and Appendix Table S3). The SAXS data and the corresponding pair distance distribution functions, *p(r),* obtained by Bayesian Indirect Fourier transformations (www.bayesapp.org) for PICK1 alone (Fig 3H) changed considerably by incubation with Tat‐P_4_‐(C5)_2_ (Fig 3J), suggesting major conformational changes to the quaternary structure. The changes were most evident at low q/long distances (Fig 3K), indicating that the concentration‐dependent large oligomer formation previously observed for PICK1 (Karlsen *et al*, 2015) was absent from the complex with PICK1:Tat‐P_4_‐(C5)_2_. Moreover, whereas the forward scattering, *I(0)*, of PICK1 without Tat‐P_4_‐(C5)_2_ showed a clear concentration dependence consistent with oligomerization as previously observed (Karlsen *et al*, 2015), *I(0),* of the PICK1:Tat‐P_4_‐(C5)_2_ complex averaged ~4 PICK1 masses (229 kDa) across the PICK1 concentration range without any concentration dependence (Fig 3G–K, and Appendix Table S3). In conclusion, this demonstrates the formation of a stable, compact, tetrameric PICK1 complex by Tat‐P_4_‐(C5)_2_. Although structurally stabilized by Tat‐P_4_‐(C5)_2,_ PICK1 was still flexible in solution (Appendix Fig S6) and its shape cannot be represented by a single rigid structure (Karlsen *et al*, 2015). Consequently, we used ensemble optimization method (EOM) (Bernadó *et al*, 2007; Tria *et al*, 2015) on the 2 mg/ml dataset of PICK1 with Tat‐P_4_‐(C5)_2_ to investigate the structural organization of the complex. EOM sampling of multiple configurations of dimeric PICK1 fitted the data poorly (Fig EV3A–C), so we constructed tetrameric configurations of PICK1, including a circular (Fig EV3D–F), elongated (Fig EV3G–I), and a side‐by‐side configuration (Fig EV3J‐L) and tested how they fitted the data. The most reliable fit of the SAXS data (simple model, low χ^2^) was obtained for a configuration of a compact tetrameric state (Fig EV3J–L), where the BAR domains align in a side‐by‐side configuration (Fig 3J) and the molecular model corresponding to this configuration is shown in Fig 3L. Taken together, the model independent SAXS analysis show that Tat‐P_4_‐(C5)_2_ induces a stable, compact, tetrameric state of PICK1 with the EOM analysis suggesting configurations with the PDZ domains from two individual PICK1 dimers within distances that can be bridged by the bivalent peptide.

### Tat‐C5 and Tat‐P_4_‐(C5)_2_ are membrane permeable and interfere with hippocampal PICK1 function *ex vivo*

**Figure 4:**
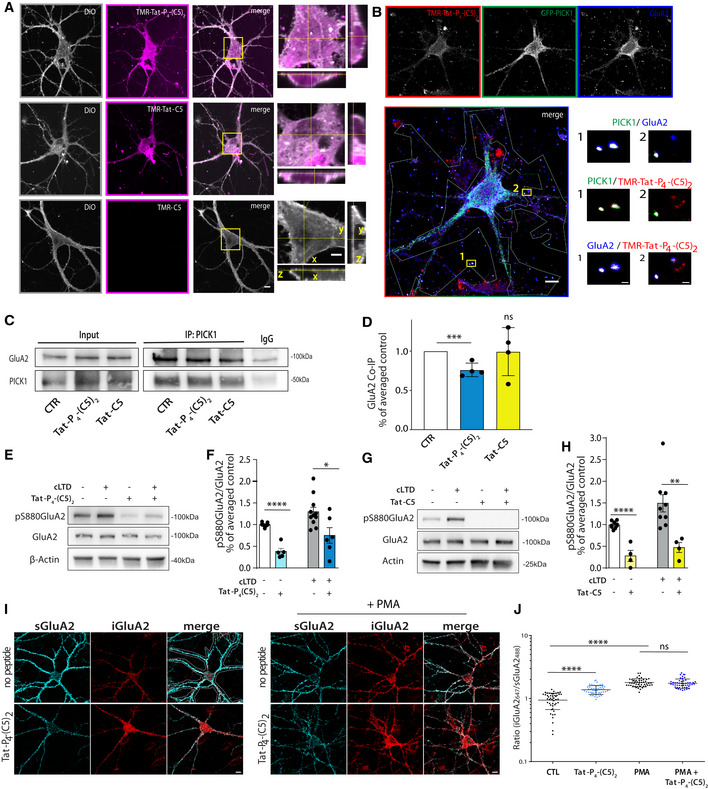
Tat‐P_4_‐(C5)_2_ is membrane permeable and compromise PICK1:GluA2 functional interaction in hippocampal neurons ARepresentative confocal images of hippocampal neurons showing membrane penetration of TMR‐Tat‐P_4_‐(C5)_2_ and TMR‐Tat‐C5 (5 μM) (magenta), but not the control TMR‐C5 (all 20 μM). The cell membrane was stained with DiO (gray). Scale bars: 10 μm and 5 μm on the zooms/orthogonal views.BRepresentative confocal images of hippocampal neurons transduced with the viral vector encoding GFP‐PICK1 and incubated with 5 nM TMR‐Tat‐P_4_‐(C5)_2_. Partial colocalization between TMR‐Tat‐P_4_‐(C5)_2_ (red), GFP‐PICK1 (green), and GluA2 (blue) is seen in insert zooms. Experiment was done three times. Scale bars: 10 μm and 1 μm on the zooms.CRepresentative immunoblots from hippocampal slices following treatment with Tat‐P_4_‐(C5)_2_ or Tat‐C5 (bath application, 20 μM).DDensitometric analysis of immunoblots from (C) shows reduced PICK1:GluA2 Co‐IP following incubation with Tat‐P_4_‐(C5)_2_ but not Tat‐C5. Bar graphs show mean with error bars as SEM (one‐way ANOVA followed by Dunnett's multiple comparison test, ***P ≤ 0.001, *n* = 4/group).ERepresentative immunoblots from hippocampal slices following Tat‐P_4_‐(C5)_2_ bath application (20 μM) show reduction in the phosphorylation level of S880 GluA2 both under basal condition and after chemical LTD (cLTD).FBar graphs show densitometric analysis of immunoblots from (E).GRepresentative immunoblots from hippocampal slices following Tat‐C5 bath application (20 μM) show reduction in the phosphorylation level of S880 GluA2 both under basal condition and after chemical LTD (cLTD).HBar graphs (right panel) show densitometric analysis of immunoblots from (G).IRepresentative confocal images of hippocampal neurons stained for surface GluA2 (sGluA2, light blue) and internalized GluA2 (iGluA2, red). Treatment with Tat‐P_4_‐(C5)_2_ (20 μM) significantly increases the constitutive, but not PMA induced internalization. Scale bars 10 μm.JQuantification of iGluA2/sGluA2 within the region of interest for individual neuron (see I, top right corner). Tat‐C5 did not increase GluA2 internalization (see Appendix Fig S9D–F).Data information: Statistics in (F and H) was done by two‐way ANOVA, followed by Sidak post‐test, which revealed a significant effect of the peptide (F(1, 192)  = 27,17), *P ≤ 0.05, **P ≤ 0.01, *n* = 4–8 as indicated by individual values. Similar analysis in (J), PMA treatment (F(1, 192) = 217.6)) and a significant interaction factor (F(1, 192) = 40,39), ****P ≤ 0.0001, *n* = 46–50 neurons/group as indicated by individual values. All data expressed as mean with error bars as SEM.

To confirm that the Tat sequence confers membrane permeability, dissociated hippocampal neurons were incubated with the TMR‐labeled peptides TMR‐Tat‐P_4_‐(C5)_2_, TMR‐Tat‐C5, or TMR‐C5 together with the membrane dye DiO. Both of the Tat‐fused peptides labeled neurons, whereas C5 alone did not (Fig 4A). Inspection of the 3D profile of the somatic region further revealed that the DiO staining, and thus membrane, surrounded a substantial fraction of the fluorescent signal from both TMR‐Tat‐P_4_‐(C5)_2_ and TMR‐Tat‐C5, consistent with presence of the peptides in the cytosol (Fig 4A, boxed area highlighted in right panels). TMR‐Tat‐C5 in general, however, showed a more punctate distribution, whereas the TMR‐Tat‐P_4_‐(C5)_2_ was mostly diffuse (Fig 4A).

To enable visualization of synaptic PICK1, we used a knock‐down and replacement strategy (Citri *et al*, 2010) to exchange endogenous PICK1 with GFP‐PICK1 in hippocampal neurons (Fig 4B). As previously shown, GFP‐PICK1 colocalized with GluA2 containing AMPARs, both in the somatic region and in punctate structures associated with the dendritic compartment (Fig 4B, zooms). Upon incubation (1 h) of the neurons with TMR‐Tat‐P_4_‐(C5)_2_, we observed a punctate localization of the peptide signal that was associated with dendritic structures outside the somatic region and this signal showed partial overlap with the GFP‐PICK1 and GluA2 signal (Fig 4B, zooms). These data demonstrate penetration of Tat‐P_4_‐(C5)_2_ into neurons and are consistent with subsequent *in vitro* target engagement of Tat‐P_4_‐(C5)_2_ with PICK1.

To further substantiate this conclusion, we knocked down PICK1 expression (without replacement) in the hippocampal neurons, which significantly reduced the amount of TMR‐Tat‐P_4_‐(C5)_2_ signal in agreement with the reduced level of the target (Appendix Fig S7). We also observed clear colocalization of the TMR‐Tat‐P_4_‐(C5)_2_ signal with GFP‐PICK1 in HEK293 cells; however, this colocalization was neither seen for a PICK1 mutant with compromised PDZ binding (GFP‐PICK1 A87L) nor with GFP alone (Appendix Fig S8A–I). The compromised binding of PICK1 A87L to TMR‐Tat‐P_4_‐(C5)_2_ was confirmed by FP binding (Appendix Fig S8K). Finally, we were able to pull down GFP‐PICK1, but not GFP‐PICK1 A87L, from the transfected HEK293 cells using a biotinylated Tat‐P_4_‐(C5)_2_ (Appendix Fig S8J), directly supporting target engagement of TMR‐Tat‐P_4_‐(C5)_2_ with PICK1.

To address if Tat‐P_4_‐(C5)_2_ or Tat‐C5 could disrupt the interaction between PICK1 and AMPARs in neurons, we performed co‐immunoprecipitation (co‐IP) experiments. Incubation of hippocampal slices with Tat‐P_4_‐(C5)_2_, or Tat‐C5, significantly reduced the co‐IP of GluA2 by PICK1 (Fig 4C and D). Similarly, a reduction was observed in the reverse co‐IP (Appendix Fig S9). Next, we addressed whether the peptides could interfere with the functional regulation of AMPARs by PICK1. Indeed, both peptides significantly reduced phosphorylation of Ser880 in GluA2 (Lu & Ziff, 2005), both under basal conditions and after a chemical long‐term depression (LTD) protocol involving NMDAR‐dependent kinase activation (Fig 4E–H). Finally, since PICK1 is believed to play a critical role in AMPAR trafficking, we addressed whether the peptides might affect GluA2 internalization in cultured hippocampal neurons. Somewhat surprisingly, Tat‐P_4_‐(C5)_2_ significantly increased constitutive internalization of surface labeled GluA2. Activation of protein kinase C (PKC) by PMA treatment further increased GluA2 internalization, which occluded the effect of Tat‐P_4_‐(C5)_2_ (Fig 4I and J). Importantly, we observed a similar effect on GluA2 trafficking upon shRNA‐mediated knock‐down of PICK1 (Appendix Fig S10A–C). In contrary, Tat‐C5 neither affected constitutive nor PMA induced GluA2 internalization (Appendix Fig S10D–F). In summary, these data support that both peptides are membrane permeable and reduce GluA2 S880 phosphorylation, but only Tat‐P_4_‐(C5)_2_ can disrupt the PICK1:GluA2 complex and increase constitutive internalization of GluA2.

### Tat‐P_4_‐(C5)_2_, but not Tat‐C5, reduces functional interaction of PICK1 with AMPAR in the spinal cord

**Figure 5:**
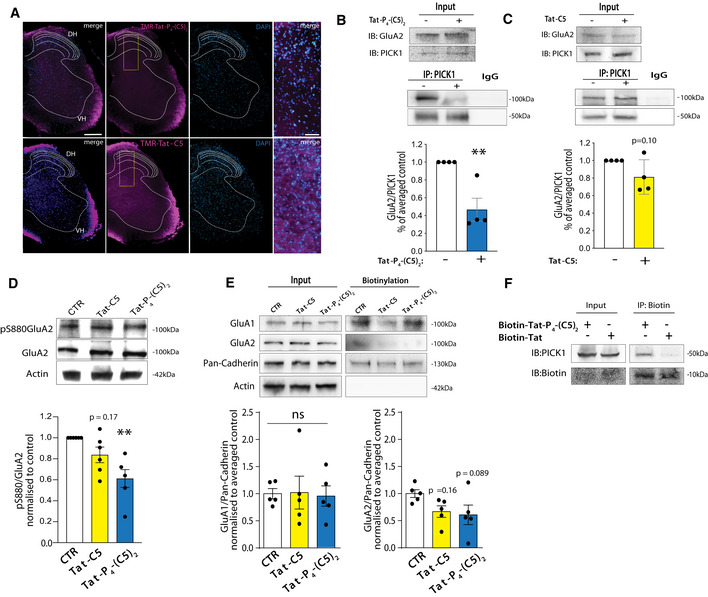
Tat‐P_4_‐(C5)_2_ interferes with PICK1:GluA2 functional interaction in mouse spinal cord ARepresentative coronal slices from lumbar spinal cord show the presence of both TMR‐Tat‐P_4_‐(C5)_2_ and TMR‐Tat‐C5 peptides (*magenta*) 1 h after i.t. administration in naïve mice. Scale bar 200 μm. DH dorsal horn, VH ventral horn. Insert zooms of merged channels (right panels), scale bar 50 μm.BRepresentative immunoblots of co‐immunoprecipitated PICK1:GluA2 from spinal cord lumbar tract total lysates demonstrate partial disruption of this interaction 1 h after 20 μM i.t. injection of Tat‐P_4_‐(C5)_2_. Densitometric analysis of immunoblots indicates a significant effect following Tat‐P_4_‐(C5)_2_ treatment, and graph shows mean ± SEM and individual points, unpaired *t*‐test, **P ≤ 0.01, *n* = 4/group.CRepresentative immunoblots of co‐immunoprecipitated PICK1:GluA2 from spinal cord lumbar tract total lysates demonstrates no disruption of this interaction 1 h after 20 μM i.t. injection Tat‐C5. Densitometric analysis of immunoblots indicates no significant effect of Tat‐C5, and graph shows mean ± SEM and individual points, unpaired *t*‐test, *P* = 0.10, *n* = 4/group.DRepresentative immunoblots of lumbar spinal cord total lysates exhibit decreased pS880‐GluA2 total levels following 20 μM Tat‐P_4_‐(C5)_2_, and Tat‐C5 i.t. injections compared to saline injected animals. Densitometric analysis of immunoblot indicates a significant effect only for Tat‐P_4_‐(C5)_2_ (graph show mean ± SEM and individual points, one‐way ANOVA followed by Dunnett′s multiple comparison test, **P ≤ 0.01, *n* = 5–6/group as indicated by individual values).ESurface biotinylation of spinal cord slices under basal condition demonstrates unchanged GluA1 and GluA2 surface level upon Tat‐P_4_‐(C5)_2_, Tat‐C5 peptides incubation compared to the untreated condition (CTR). Densitometric analysis of immunoblots shows that the reduction in mean surface for GluA2 in not significant (graph show mean ± SEM and individual points, one‐way ANOVA followed by Dunnett′s multiple comparison test, *n* = 5/group as indicated by individual values).FImmunoblot of PICK1 following pull‐down from lumbar spinal cord with Biotin‐Tat‐P_4_‐(C5)_2_ or Biotin following 20 μM i.t. administration demonstrating *in vivo* target engagement.

PICK1 has been proposed as a putative target for treatment of neuropathic pain (Garry *et al*, 2003; Wang *et al*, 2011, 2016). To address pharmacokinetic and pharmacodynamic properties *in vivo*, peptides were administered intrathecally (i.t.) in naïve mice. TMR‐Tat‐P_4_‐(C5)_2_ and TMR‐Tat‐C5 (20 μM) were clearly visible in both the ventral and dorsal parts of the spinal cord 1 h after injection (Fig 5A). Moreover, TMR‐Tat‐C5 (Fig 5A, bottom) appeared to distribute more homogenously to the gray matter part of the spinal cord than TMR‐Tat‐P_4_‐(C5)_2_ (Fig 5A, top). Administration of Tat‐P_4_‐(C5)_2_ also reduced co‐IP of GluA2 by PICK1 from the spinal cord (Fig 5B), similar to our observation in hippocampal slices, whereas Tat‐C5 did not significantly reduce GluA2 co‐IP by PICK1 (Fig 5C), suggesting different pharmacodynamic/kinetic properties of the two peptides also in the spinal cord upon i.t. administration. Next, we tested the effect of both peptides on GluA2 Ser880 phosphorylation levels on spinal cord slices and, in contrast to hippocampal slices, Tat‐P_4_‐(C5)_2_, but not Tat‐C5, significantly reduced GluA2 S880 phosphorylation (Fig 5D). To address putative effects on AMPAR surface levels, we performed surface biotinylation experiments on spinal cord slices. Neither peptides affected GluA1 surface levels, whereas both peptides gave rise to a small but non‐significant reduction in surface GluA2 levels (Fig 5E). Lastly, the ability of Tat‐P_4_‐(C5)_2_ to engage the target protein *in vivo* following i.t. administration in naïve mice was confirmed by a pull‐down experiment between PICK1 protein and an N‐terminally biotinylated Tat‐P_4_‐(C5)_2_ peptide (Fig 5F).

### Tat‐P_4_‐(C5)_2_, but not Tat‐C5, reduces surface AMPAR levels to alleviate neuropathic pain

**Figure 6:**
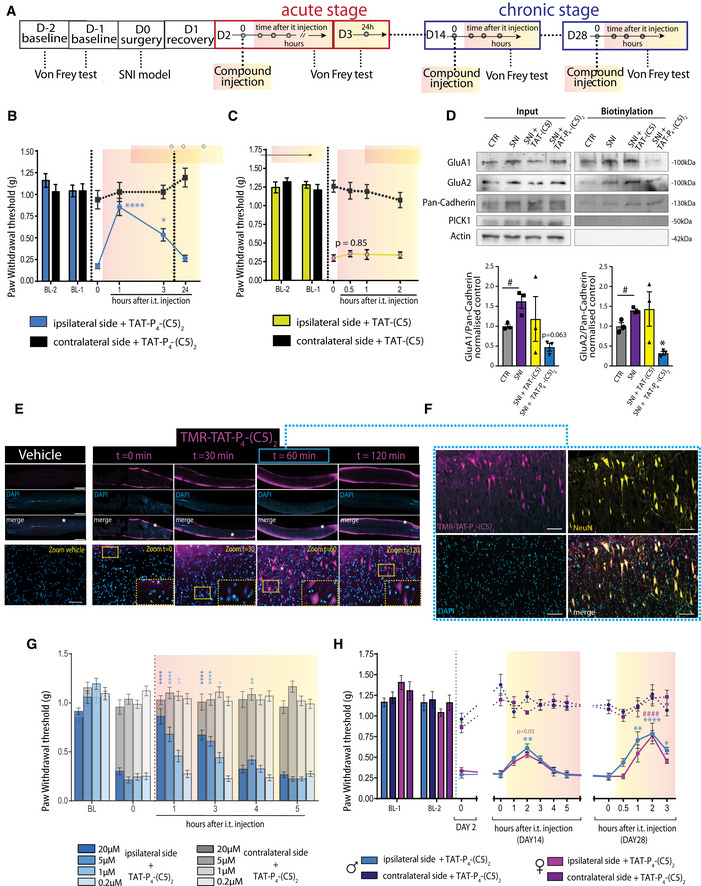
Tat‐P_4_‐(C5)_2_, but not Tat‐C5, reduce AMPAR surface expression and allodynia in SNI model of neuropathic pain ADiagram of *in vivo* timeline (D = day).Bi.t. administration (20 μM) of Tat‐P_4_‐(C5)_2_ significantly reduced ipsilateral paw hypersensitivity of SNI mice in the initiation stage (2 days after injury) of the spared nerve injury model at time points 1 and 3 h (*n* = 7).Ci.t. administration (20 μM) of Tat‐C5 did not affect ipsilateral paw hypersensitivity of SNI mice in the initiation stage (2 days after injury) of the spared nerve injury model (*n* = 8).DRepresentative Western blots (top) for GluA1 and GluA2 following surface biotinylation of spinal cord slices. Densitometric analysis (bottom) of immunoblots. We observed upregulation of both GluA1 and GluA2 surface levels following SNI surgery compared to non‐operated animal CTL (unpaired *t*‐test GluA1: t(4) = 3.372, GluA2: t(4) = 3.991,#*P* ≤ 0.05, *n* = 3/group). Tat‐P_4_‐(C5)_2_ but not Tat‐C5 significantly reduced the SNI‐induced GluA2 surface expression and shows a strong tendency for GluA1 as well. Representative immunoblot shows unaltered PICK1 total level in the total lysate in the four conditions.EImages of lumbar spinal cord sagittal sections showing the time course of TMR‐Tat‐P_4_‐(C5)_2_ (magenta) after i.t. administration (20 μM) in SNI mice showing maximal accumulation in cells after 60 min. Scale bar 1,000 μm and 100 μm in zooms.FOverlay with immunohistochemical staining for the neuronal marker NeuN (yellow) highlights strong neuronal tropism of TMR‐Tat‐P_4_‐(C5)_2_ (magenta). Scale bar 100 μm.GDose‐dependent von Frey test in the acute phase (2 days after injury) of the spared nerve injury model following i.t. administration of Tat‐P_4_‐(C5)_2_ (*n* = 8/group) did not affect ipsilateral paw hypersensitivity of SNI mice and lasts up to 4 h post‐injection.Hi.t. administration of Tat‐P_4_‐(C5)_2_ (20 μM) at days 14 and 28 after injury in the maintenance phase of SNI model in mice shows significant increase in pain withdrawal threshold up to 3 h with equal efficacy in male and female mice (compared to time 0 pre‐injection, D14 *n* = 8/group/gender, D28 *n* = 6 male, *n* = 7 female).Data information: In (B and C), ipsi‐ and contralateral paw withdrawal thresholds at different time points were compared to time 0 using two‐way repeated‐measures ANOVA followed by Dunnett's multiple comparison test (*P <* *0.05, ****P <* *0.0001). In (D), GluA1/Pan‐Cadherin levels were compared using one‐way ANOVA followed by Dunnett′s multiple comparison test, GluA2: F_(2,6)_=6.256, *P ≤ 0.05; GluA1: F_(2,6)_=2.843 *P* = 0.096, *n* = 3/group). In (G), ipsi‐ and contralateral paw withdrawal thresholds at different time points were compared to time 0 using two‐way ANOVA followed by Dunnett's multiple comparison test (*P <* *0.05, **P <* *0.01, ****P <* *0.0001). All data expressed as mean ± SEM. In (H), ipsi‐ and contralateral paw withdrawal thresholds at different time points were compared to time 0 using two‐way repeated‐measures ANOVA followed by Dunnett's multiple comparison test (*P <* *0.05, **P <* *0.01, ****/^####^ *P *<* *0.0001).

To test whether the PICK1 inhibitors can alleviate neuropathic pain symptoms that involve central sensitization, we employed the spared nerve injury model (SNI) on adult mice (Decosterd & Woolf, 2000; Fig 6A). On day two after the nerve injury, the model produced a robust hypersensitivity (reduced paw withdrawal threshold (PWT)) to mechanical stimuli applied to the ipsilateral hind paw without affecting the contralateral hind paw (Fig 6B). I.t. administration of Tat‐P_4_‐(C5)_2_ significantly alleviated SNI‐induced hypersensitivity 1 h after administration (compared to the pre‐injection time point, 0 h) reaching a level similar to both the contralateral paw and pre‐injury level. The pain threshold was still significantly increased at 3 h (compared to the pre‐injection time point, 0 h), while no effect of the peptide was seen after 24 h (Fig 6B). Intraplantar administration of the same dose did not affect PWT and even a 10‐fold higher intraplantar dose did not significantly alleviate allodynia (Appendix Fig S11), suggesting little peripheral effect of Tat‐P_4_‐(C5)_2._

**Figure EV4:**
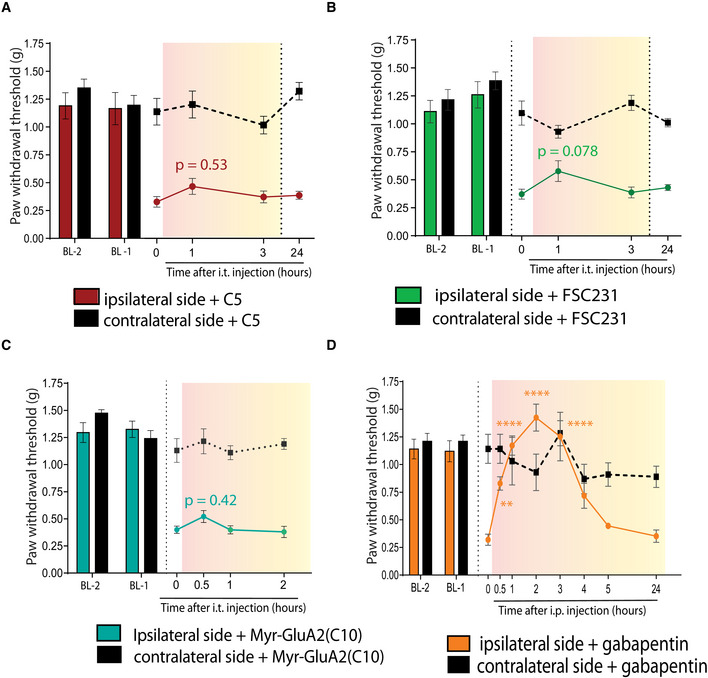
Other PICK1 inhibitors fail to attenuate mechanical hyperalgesia but gabapentin does Ai.t. administration (20 μM) of C5 did not significantly change PWT in the initiation stage (2 days after injury) of SNI‐induced hypersensitivityBi.t. administration (20 μM) of the small‐molecule PICK1 inhibitor FSC231 did not significantly change PWT in the initiation stage (2 days after injury) of SNI‐induced hypersensitivityCi.t. administration (20 μM) of myrGluA2 did not significantly change PWT in the initiation stage (2 days after injury) of SNI‐induced hypersensitivityDi.p. administration of gabapentin (30 mg/kg) produced full recovery of ipsilateral paw threshold in the initiation stage (2 days after injury) of SNI‐induced hypersensitivity serving as reference compound in acute phase SNI animals at time 1, 2, 3 h.Data information: Data on graphs are mean ± SEM. Ipsi‐ and contralateral paw withdrawal thresholds at different time points were compared to time 0 using two‐way ANOVA followed by Dunnett's multiple comparison test (**P <* *0.01, ****P <* *0.0001). (A‐C) *n* = 8 mice/group, (D) *n* = 4 mice.

In concordance with our previous results, i.t. administration of C5 did not affect the PWT, neither did Tat‐C5 (Figs 6C and EV4A). Also, the small‐molecule inhibitor of PICK1 FSC231 (Thorsen *et al*, 2010) as well as a myristoylated GluA2 inhibitory peptide (Garry *et al*, 2003) failed to elicit a significant effect on the PWT (Fig EV4B and C), whereas gabapentin, a first‐line treatment for neuropathic pain, reversed PWT to baseline levels of the contralateral paw and prior to nerve injury of the ipsilateral paw (Fig EV4D). Surface biotinylation on spinal cord slices showed upregulation of both surface GluA1 and GluA2 following SNI, while treatment of SNI animals with Tat‐P_4_‐(C5)_2_, but not Tat‐C5, reduced the surface level of both GluA1 and GluA2 below control level (Fig 6D).

Following i.t. administration in SNI animals, TMR‐Tat‐P_4_‐(C5)_2_ initially (0 min and 30 min) distributed along the white matter of the spinal cord, and at later time points (60 min and 120 min), it was clearly visible in the gray matter as well (Fig 6E). We also noticed that the peptide signal in the gray matter was exclusively confined toward neurons (NeuN) (Fig 6F) and not on glial cells (GFAP) (Appendix Fig S12), suggesting neuronal tropism of TMR‐Tat‐P_4_‐(C5)_2_.

Mice subjected to SNI showed similar alleviation of mechanical allodynia after the first (2 days after operation) and second (3 days after operation) administration of Tat‐P_4_‐(C5)_2_ (Appendix Fig S13) indicating no development of tolerance for Tat‐P_4_‐(C5)_2_ upon repeated administration. Using this setup, we could also demonstrate that the effect of Tat‐P_4_‐(C5)_2_ on PWT was dose‐dependent (Fig 6G). Administration of Tat‐P_4_‐(C5)_2_ also significantly increased PWT of the animals at 14 days after injury and again at 28 days after injury (Fig 6H). Initial inflammation ceases 9–10 days after surgery, and therefore, these later time points are believed to better mimic chronic pain in humans (Percie du Sert & Rice, 2014). Injected gabapentin had comparable efficacy 14 days after nerve injury (Appendix Fig S14), suggesting that Tat‐P_4_‐(C5)_2_ may be an attractive lead for development of efficacious neuropathic pain therapy.

### Tat‐P_4_‐(C5)_2_ represses expression of CP‐AMPARs and reduce neurotransmission in the dorsal horn of SNI mice

**Figure 7:**
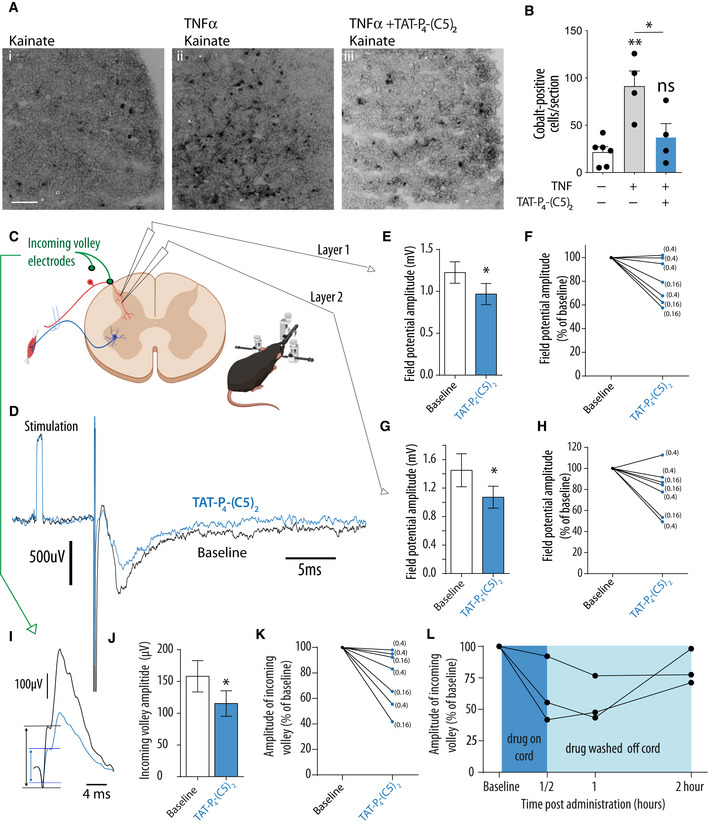
Tat‐P_4_‐(C5)_2_ reduces functional CP‐AMPAR expression and electrophysiological transmission in the spinal cord ARepresentative images of kainate‐induced cobalt uptake in spinal cord slices from p14 mouse pups treated with (i) saline, (ii) TNFα, and (iii) TNFα + Tat‐P_4_‐(C5)_2_. Scale bar: 100 μm.BQuantification of cobalt positive (black soma) cells. Each data point represents the average of 4‐6 25‐μm sections from a single 400 μm slice. Bars show means of 4–6 slices, and error bars represent SEM (one‐way ANOVA followed by Bonferroni's multiple comparison test, *P < 0.05, **P < 0.01).CIn vivo electrophysiological recordings for the dorsal spinal cord before and after peptide administration following stimulation of the peripheral sural nerve at intensities sufficient to activate C and Aδ fibers. Illustration of the experimental setup indicates where measurements were made (image made using Biorender.com).DExample of field potentials recorded in the same position before (black) and after (blue) peptide administration. The recording starts with a 1 mV calibration pulse followed by a stimulus artifact (S), which is truncated for the figure and finally the field potential at a depth corresponding to lamina 1. Each trace is an average of at least 10 successive trials.EBar chart showing the mean amplitude of the field potential at lateral positions in lamina 1 recorded before and approximately 30 min after peptide administration, which was significantly reduced (Wilcoxon matched pairs, *P =* *0.0313, W = −26, *n* = 7 mice, error bars: SEM).FLine chart to show data from (E), as separate data points for individual mice expressed as a percentage relative to baseline measurements. From this, it can be seen that the response was variable across mice with just over half showing a large reduction and the remaining mice only showing a relatively minor reduction (with no reduction in one mouse). The von Frey thresholds for the individual mice before the experiment are shown next to each line.GBar chart showing the mean amplitude of the field potential at lateral positions in lamina 2 recorded before and 30 min after peptide administration, which was significantly reduced (Wilcoxon matched pairs, *P =* *0.0313, W = −26, *n* = 7 mice, error bars: SEM).HLine chart to show data from (G), as separate data points for individual mice expressed as a percentage relative to baseline measurements. From this, it can be seen there was reduction in all but 1 mouse, although the magnitude of the reduction was again variable across mice. The von Frey thresholds for the individual mice before the experiment are shown next to each line.IExample of cord dorsum potentials recorded following stimulation of the peripheral sural nerve. The initial 3 inflections represent the incoming volley of action potentials along the afferent axons just before they enter the spinal cord. This was recorded before (black) and again 30 min post‐peptide administration (blue). In this particular example, an extreme drop in the amplitude of the incoming volley is observed post‐administration.JBar chart showing that the mean amplitude of the incoming volley is reduced post‐administration (Wilcoxon matched pairs, *P =* *0.0156, W = −28, *n* = 7 mice, error bar: SEM).KLine chart to show data from (J), as separate data points for individual mice expressed as a percentage relative to baseline measurements. From this, it can be seen that the response was variable across mice with half showing a large reduction and the remaining mice only a minor reduction.LAmplitude of the incoming volley expressed as a percentage of the baseline recording prior to peptide administration. In these 3 mice, a reduction in the incoming volley at 30 min post‐administration is seen. After this recording, the remaining peptide was removed by washing the spinal cord and two further recordings made at 1 and 2 h. Here, it can be seen that the incoming volley does not deteriorate further but stabilizes or starts to return toward baseline levels confirming that the initial reduction was not due to a deterioration in the health of the animal or damage to the roots.

Both inflammatory and neuropathic pain models have been shown to cause an upregulation of CP‐AMPARs (Vikman *et al*, 2008; Gangadharan *et al*, 2011; Chen *et al*, 2013). Since PICK1 has been shown to underlie maladaptive expression of CP‐AMPARs in midbrain and hippocampus (Dixon *et al*, 2009; Wolf & Ferrario, 2010; Luscher & Malenka, 2011), we asked whether this might also be the case in the spinal cord. To assess functional expression of CP‐AMPARs in neurons, we used kainate‐induced cobalt uptake on spinal cord slices and as previously reported modest staining of cobalt was observed under basal conditions (Fig 7Ai and B) (Engelman *et al*, 1999). To mimic the effect of peripheral injury on spinal cord slices, we applied TNFα which gave rise to robust induction of CP‐AMPAR function (Fig 7Aii and B) (Xu *et al*, 2006; Wigerblad *et al*, 2017), and indeed, this induction was significantly decreased by co‐incubation of TNFα with Tat‐P_4_‐(C5)_2_ (20 μM) (Fig 7Aiii and B), suggesting that Tat‐P_4_‐(C5)_2_ can interfere with functional expression of CP‐AMPARs as one mechanism to reduce pain transmission.

Finally, to directly determine the effect of Tat‐P_4_‐(C5)_2_ on DRG excitability and transmission in the spinal cord, *in vivo* electrophysiological recordings were performed in adult decerebrate SNI mice to avoid possible interactions with anesthetics (Fig 7C and D). Hypersensitivity of the operated animals was confirmed by von Frey measurements prior to DRG excitability and transmission experiments (threshold for injured paw in individual mice is depicted in parenthesis Fig 7F and H). The sural nerve was stimulated and the incoming volley of action potentials along the DRG were recorded (Lloyd and McIntyre 1948), together with the resulting field potentials in the spinal cord at depths consistent with lamina 1 and 2 (Fig C, D).

Baseline recordings were made before Tat‐P_4_‐(C5)_2_ (20 μM) was applied to the exposed surface of the spinal cord and recordings performed again in the same location approximately 30 min after peptide application. Averaged field potentials in the spinal cord at depths corresponding to lamina 1 and lamina 2 were significantly reduced at 30 min following peptide administration relative to baseline measurements (Fig 7E and G) although there was significant variability in the magnitude of this effect between mice (Fig 7F and H). This confirms that the peptide reduced the net synaptic activity in the dorsal horn.

There was also a significant initial depression of the incoming volley observed at 30 min after administration (Fig 7I–K). In half of the mice, this was pronounced, as illustrated in Fig 7K. In the remaining mice, a smaller depression was observed. To confirm that this reduction was not due to damage to the dorsal roots or deterioration in the health of the mouse in three of the mice, the peptide was washed off the surface of the cord (Fig 7L). In all 3 mice, consistent with the time course in von Frey experiments, the incoming volley amplitude stabilized or started to return by 2 h, demonstrating that the effect was related to the action of Tat‐P_4_‐(C5)_2_, rather than a deterioration in the health of the animals of damage to the roots.

Taken together, these data demonstrate that Tat‐P_4_‐(C5)_2_ in addition to the modulation of central sensitization may also exert its action by suppression of excitability of the DRG axons at, or around, the dorsal root entry zone, which are likely significantly more accessible to the peptide than the secondary afferents in the spinal cord.

## Discussion

In this study, we develop two cell‐permeable, high‐affinity inhibitors, Tat‐C5 and Tat‐P4‐(C5)_2_, of the PICK1 PDZ domain with the purpose of disrupting the interaction of PICK1 with AMPARs and thereby reverse central sensitization and its related maladaptive plasticity believed to be associated with neuropathic pain. We demonstrate that the five C‐terminal residues of DAT (called C5) are sufficient for maintaining affinity of the endogenous DAT ligand. Conjugation of C5 to the HIV‐derived Tat sequence (Tat‐C5), which rendered the peptide membrane permeably, serendipitously also increased affinity more than 100‐fold—an effect that was specific for the PICK1 PDZ domain leading to increased selectivity. Our structural data suggest that this affinity gain results from interaction of Tat with residues extending from the binding groove and all the way to the opposite side of the PDZ domain.

In a parallel approach, we show that linking two C5 peptides together with a PEG linker result in a 15‐fold affinity gain, while addition of the Tat sequence increased affinity further 50‐fold leading to more than 1,000‐fold higher affinity for PICK1 than the C5 peptide alone. Indeed, bivalency is well known to endow biological molecules with high affinity/avidity and it is a well‐known strategy in receptor pharmacology (Portoghese, 2001; Krishnamurthy *et al*, 2007) as well as in the case of the analogous bivalent inhibitor of PSD‐95 (Sainlos *et al*, 2011; Bach *et al*, 2012). However, whereas these targets traditionally have been bivalent themselves, such as the tandem PDZ domain of PSD‐95 or strict PDZ dimers (Paduch *et al*, 2007), we found that Tat‐P_4_‐(C5)_2_ works by bridging two PDZ domains from individual PICK1 dimers to produce a novel tetrameric complex. Importantly, although analogous to our previously published bivalent PSD‐95 inhibitors, Tat‐P_4_‐(C5)_2_ does not bind PSD95, suggesting that its function in pain does not relate to the ability of PSD‐95 to sustain hyperalgesia (D'Mello *et al*, 2011). Our finding implies that bivalent inhibitors may hold an unforeseen potential as high‐affinity inhibitors—not only of tandem domains, but also of PDZ domain proteins with propensity to oligomerize, such as PDZ‐GEF and LARG (Paduch *et al*, 2007).

Interestingly, the *in vivo* efficacy of Tat‐P_4_‐(C5)_2_ clearly exceeded the one of Tat‐C5, as well as the efficacy of two other PICK1 inhibitors (myr‐GluA2 and FSC231), with respect to relieving mechanical allodynia in the SNI model of neuropathic pain. Tat‐P_4_‐(C5)_2_ was likewise considerably better than Tat‐C5 at; i) blocking the interaction between PICK1 and GluA2 according to co‐immunoprecipitation analysis; ii) regulating GluA2 Ser880 phosphorylation, and iii) reducing AMPAR surface levels. It is possible that this in part may reflect better pharmacokinetic properties of Tat‐P_4_‐(C5)_2_ compared to Tat‐C5. That is, although distribution and membrane permeability appeared to be better for Tat‐C5 (Figs 4A and 5A), the *in vivo* stability of Tat‐P_4_‐(C5)_2_ is likely better than that of Tat‐C5 as suggested by our plasma stability measurements (Fig EV1H). On the other hand, the differences observed in the surface biotinylation assay (Fig 6D), which was performed on spinal cord slices *ex vivo,* suggest that also the pharmacodynamic properties of the two compounds differ. This was specifically supported by the ability of Tat‐P_4_‐(C5)_2_, but not Tat‐C5, to affect GluA2 internalization in dissociated hippocampal neurons.

**Figure EV5:**
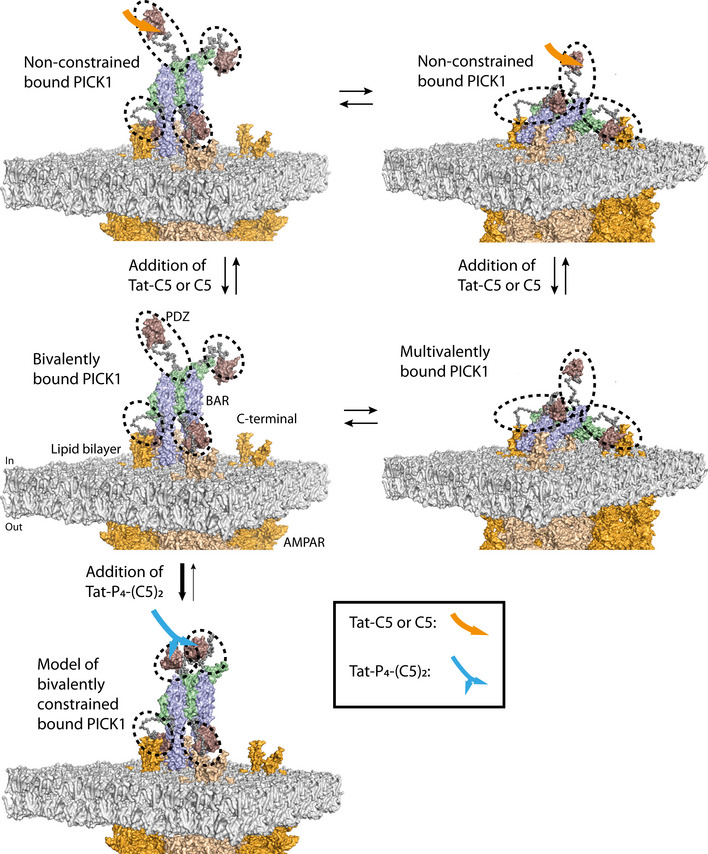
Illustration of proposed model for the ability of Tat‐P_4_‐(C5)_2_ to increase macroscopic off‐rate of PICK1 bound to receptors embedded in the membrane PICK1 binds to membrane‐embedded receptors, such as the AMPARs (orange), in two distinct states, a bivalent and a multivalent bound conformation, mainly driven by lipid binding motifs (black dashed circles) positioned in the PDZ domain and the amphipathic BAR‐PDZ linker (Erlendsson *et al*, 2019). Upon addition of Tat‐P_4_‐(C5)_2_, two or more PDZ domains of the PICK1 tetramer and the lipid binding motifs gets constrained, thereby favoring an upright position of PICK1, with a maximum of two PDZ domains and lipid binding regions of the PICK1 tetramer binding to the membrane and membrane‐embedded receptors. Addition of Tat‐C5 or C5 does not constrain the PDZ domains, which are then free to exchange between the bivalent upright PICK1 and the multivalent membrane parallel bound PICK1, thereby not affecting the macroscopic off‐rate. The model was created using PyMoL 2.0. The tetrameric PICK1 model was generated in earlier EOM modeling, and the position of the model is based on earlier work (Erlendsson *et al*, 2019). The structure of AMPARs (PDB: 5VHW) was positioned into a POPE lipid bilayer manually, and a *de novo* model of the GluA2 cytoplasmic tail (UniProt: P42262, residues 834‐883) was generated using PEP‐FOLD 3 (Thevenet *et al*, 2012; Shen *et al*, 2014; Lamiable *et al*, 2016) and orientated to the C‐terminal helix of the AMPAR structure manually.

From a mechanistic perspective, we propose that the differences in efficacy between the two peptides relate to the increased ability of Tat‐P_4_‐(C5)_2_ to dissociate a preformed cluster of PICK1 bound to membrane‐embedded ligands as observed on SCMSs. For multivalent interactions, the macroscopic dissociation rate is composed several individual microscopic dissociation rates. In the first dissociation step, the dissociation rate is balanced by an association rate (rebinding) governed by the local concentration of the PDZ domain, rather than global concentration of PICK1, which give rise to avidity (Chi *et al*, 2010). Competitive inhibitors do not affect microscopic association or dissociation constants and, thus, cannot increase dissociation of monovalent interactions for which the macroscopic dissociation rate equals the microscopic one. For bivalent interactions, on the other hand, competitors may increase the macroscopic dissociation rate, given that it can effectively compete with association rate (rebinding) governed by the local concentration of the PDZ domain in this case. Clearly, Tat‐P_4_‐(C5)_2_ does this much more efficiently than both Tat‐C5 (Figs 2E–G and EV2) despite only ~10‐fold difference in in‐solution binding strength. We propose that PICK1 may assemble in a tetrameric configuration, similar to our SAXS structure (Fig 3L), upon binding to membrane‐embedded receptors. In this scenario, peptide binding (monovalent as well as bivalent) will interfere with PDZ interaction with ligand in the membrane. We recently demonstrated, however, that PICK1 achieve most of the avidity, not only from multiple PDZ interaction at the membrane the avidity, but rather from interaction with the lipid membrane by the amphipathic helix lining the BAR domain (Herlo *et al*, 2018; Erlendsson *et al*, 2019) and likely also the CPC loop in the PDZ domains (Shi *et al*, 2010) (Fig EV5, black dashed circles). Interestingly, according to our SAXS structure (Fig 3L), binding of Tat‐P_4_‐(C5)_2_ restricts orientation of the two PDZ domains, which may sterically compromise these membrane‐interacting motifs and thereby also the rebinding events they govern (Fig EV5, bottom). Since Tat‐C5 will not restrict the PDZ orientation (Fig EV5, bottom), this could explain why only Tat‐P_4_‐(C5)_2_ facilitate the macroscopic off‐rate.

Our trafficking experiments performed in cultured hippocampal neurons with Tat‐P_4_‐(C5)_2_ and shRNA suggest that PICK1 serves to stabilize GluA2 on the surface by saving it from constitutive internalization. This is in concurrence with previous findings in the PICK1 KO mice showing reduced extra‐synaptic, surface GluA2 (Gardner *et al*, 2005); however, little work has been done on the role of PICK1 in constitutive internalization of AMPARs. Several studies, however, show that removal of or interference with PICK1 reduces activity‐dependent internalization of GluA2 (Matsuda *et al*, 1999; Chung *et al*, 2000; Iwakura *et al*, 2001; Hanley *et al*, 2002; Seidenman *et al*, 2003; Bell *et al*, 2009). Although this might seem contradictory, we propose that the increased constitutive internalization upon interference with PICK1 might, at least in some cases, explain the reduced activity‐dependent internalization, either due to fewer mobile surface receptors for activity‐dependent endocytosis to act upon or because of normalization to different constitutive rates. Note that our data would indicate a compromised PMA‐dependent internalization in the presence of Tat‐P_4_‐(C5)_2_ if normalized to the respective constitutive internalization with and without peptide (Fig 4H).

Interestingly, the surface biotinylation experiments suggest that while Tat‐P_4_‐(C5)_2_ only modestly decreases surface GluA2 in the spinal cord under basal conditions, it robustly reduces surface GluA1 and GluA2 after induction of neuropathic pain. Moreover, Tat‐P_4_‐(C5)_2_ reduced the TNF‐induced increase in CP‐AMPARs, supporting that Tat‐P_4_‐(C5)_2_ is capable of potently modulating the central sensitization often associated with neuropathic pain. Indeed, *in vivo* electrophysiology confirmed reduced transmission in layer 1 and layer 2 after stimulation of peripheral afferents; however, the measurements also revealed a robust decrease in the incoming presynaptic fiber volley in agreement with previous detection of PICK1 immunosignal in layers 1 and 2 of the spinal cord as well as in DRGs (Wang *et al*, 2011). Whether this reflects a direct effect on DRG excitability or indirect modulation by central neurons remains to be determined but regards such mechanistic speculations this finding is important when evaluating pharmacokinetic properties of putative pharmaceutical with PICK1 as target in pain.

The selective engagement of PICK1 in AMPAR surface expression in the spinal cord after SNI‐induced plasticity makes PICK1 a very promising drug target. In analogy, PICK1 seems to serve a specific role in potentiating AMPAR function during acquisition and reacquisition of cocaine dependence (Bellone & Luscher, 2006; Famous *et al*, 2008; Schmidt *et al*, 2013; Jensen, 2017), suggesting that PICK1 is a promising target in addiction. Indeed, Tat‐P_4_‐(C5)_2_ strongly reduces cocaine reinstatement in a self‐administering paradigm (Turner *et al*, 2020). Moreover, PICK1 has been evoked as a putative target in ischemia (Dixon *et al*, 2009), Alzheimer's disease (Alfonso *et al*, 2014), and Parkinson's disease (He *et al*, 2018).

In conclusion, we developed a high‐affinity inhibitor toward the PICK1 PDZ domain, Tat‐P_4_‐(C5)_2_. Tat‐P_4_‐(C5)_2_ obtains its high affinity by cross‐linking PDZ domains from individual PICK1 dimers and demonstrates favorable pharmacodynamic properties to potently alleviate neuropathic pain in both acute and chronic states, regardless of gender. We believe the peptide to be a promising lead compound and, with the proper formulation to enable appropriate administration, to be a strong candidate for extended pre‐clinical studies leading to future clinical trials for neuropathic pain following peripheral nerve damage (Costigan *et al*, 2009).

## Materials and Methods

### Protein expression and purification

*Escherichia coli* cultures (BL21‐DE3‐pLysS) transformed with a PICK1 encoding plasmid (pET41) were inoculated in 50 ml LB with kanamycin overnight and transferred into 1 L LB medium with kanamycin and grown at 37°C until OD600 = 0.6. Protein expression was induced with 1 mM IPTG and grown overnight at 20°C. Bacteria were harvested and suspended in lysis buffer containing 50 mM Tris, 125 mM NaCl, 2 mM DTT (Sigma), 1% Triton X‐100 (Sigma), 20 μg/ml DNase 1, and half a tablet of cOmplete protease inhibitor cocktail (Roche) pr. 1 l culture at pH 7.4. Suspended pellet was frozen at −80°C. The bacterial suspension was thawed and cleared by centrifugation (F20 rotor, 36,000 × *g* for 30 min at 4°C). The supernatant was collected and incubated with Glutathione‐Sepharose 4B beads (GE Healthcare) for 2 h at 4°C under gentle rotation. The beads were pelleted at 3,500 × *g* for 5 min, and supernatant was removed and beads were washed 2 times in a wash buffer consisting of 50 mM Tris, 125 mM NaCl, 2 mM DTT, and 0.01% Triton X‐100, pH 7.4. Washed beads were transferred to a PD10 gravity column and were washed additionally three times. Bead solution was incubated with thrombin overnight at 4°C under gentle rotation. Cleaved PICK1 was eluted on ice, and absorption at 280 nm was measured on a NanoDrop 2000, and protein concentration was calculated using Lambert–Beer's law (A=εcl), ε_A280_PICK1 = 32,320 (cm*mol/L)^−1^. PDZ domains for selectivity test were purified as described previously (Stiffler *et al*, 2007).

### Peptide synthesis

Fluorescently labeled peptides were conjugated by either cysteine–maleimide in the case of Oregon Green peptides or N‐terminal Ahx linkage in case of 5FAM labeling. All the FAM‐conjugated and Oregon Green‐conjugated peptides were purchased, respectively, from TAG‐Copenhagen and Schafer‐N. The peptide corresponding to the 5 most C‐terminal amino acids of the dopamine transporter C5, HWLKV, as well as 5FAM‐C5 5FAM‐Ahx‐HWLKV, Tat‐C5 YGRKKRRQRRR‐HWLKV, 5FAM‐Tat‐C5 5FAM‐Ahx‐YGRKKRRQRRR‐HWLKV, and TMR‐Tat‐C5 TMR‐Ahx‐YGRKKRRQRRR‐HWLKV, was purchased from TAG‐Copenhagen.

Bivalent ligands PEG_0_‐(HWLKV)_2_, PEG_4_‐(HWLKV)_2_, PEG_8_‐(HWLKV)_2_, PEG_12_‐(HWLKV)_2_, PEG_28_‐(HWLKV)_2_, and 5FAM‐P4‐(C5)_2_ were synthesized in‐house by solid‐phase peptide synthesis as described in Bach *et al* (2009).

TMR‐Tat‐P_4_‐(C5)_2_ (TMR‐YGRKKRRQRRR‐*N*PEG_4_‐(HWLKV)_2_) and Tat‐P_4_‐(C5)_2_ (YGRKKRRQRRR‐*N*PEG_4_‐(HWLKV)_2_) were synthesized by Wuxi AppTec Co., Ltd. (China) accordingly. All peptides were purified or delivered to > 95% purity verified by UPLC‐MS.

### Plasma stability

The peptides were dissolved to a final concentration of 50 μM in human plasma (3H biomedical, Uppsala, Sweden) containing 2% DMSO and incubated at 37°C. Aliquots (45 μl) were added directly to 26 mg guanidine hydrochloride (GnHCl), vortexed, and incubated for 2 min. 90 μl of 10% trichloroacetic acid (TCA) in acetone was then added, and the suspension was incubated at 5°C for 24 h.

The suspension was centrifuged at 18,000 *g* for 5 min. The supernatants were quantified by analytical C8 RP‐HPLC and normalized to the compound concentration at T = 0. Ligand recovery was > 82%.

### Fluorescence polarization

Fluorescence Polarization (FP) saturation binding was carried out using an increasing amount of PICK1 incubated with a fixed concentration of fluorescently labeled peptides as indicated. Competition FP was done at a fixed concentration of PICK1 and indicated fluorescent tracer, against an increasing concentration of unlabeled peptide. In general, we sought to match tracer affinities to expected competitor affinity for optimal assessment of actual binding strength (Huang, 2003). After mixing, the 96‐well plate (a black half‐area Corning Black non‐binding) was incubated for 20 min on ice, after which the FP was measured directly on an Omega POLARstar plate reader using excitation filter at 488 nm and long pass emission filter at 535 nm. The data were plotted using GraphPad Prism 8.3 and fitted to either a sigmoidal dose response for saturation experiments or One site competition for competition experiments. Kis were automatically calculated using the Cheng–Prusoff equation. All binding isotherms were repeated at least twice with different purifications of PICK1 and each time with 3 technical replicates. For selectivity screens, individual PDZ domains (42 domains + PICK1) with their respective fluorescent ligand were spotted in 96‐well plates. FP was measured in parallel for plates incubated with buffer, 10 μM of C5 or Tat‐C5.

### Nuclear magnetic resonance

Isotopically labeled PICK1 PDZ‐DAT C10 was expressed and purified as described in Erlendsson *et al* (2014). The isolated unbound PICK1 PDZ domain is not stable in solution; however, extending the PDZ domain with a cleavable (PreScission C3 protease) linker followed by the 10 C‐terminal residues of DAT yields a highly stable complex suitable for structural determination by NMR (Erlendsson *et al*, 2014). Cleaving the linker in absence of Tat‐C5 produces only very minor structural perturbation. Chemical shift and/or intensity changes observed when cleaving the linker in presence of a saturating concentration Tat‐C5 can be directly related to the displacement of C10 and subsequent binding of Tat‐C5 peptide.

NMR experiments were recorded on a Bruker Avance III HD 600 MHz spectrometer with a QCI quadrupole resonance cryoprobe. All experiments were carried out at 25°C in 150 mM NaCl, 50 mM Tris, pH 7.4, 10% D2O, 0.02% NaN3 (Sigma‐Aldrich), and 0.25 mM 4,4‐dimethyl‐4‐silapentane‐1‐sulfonic acid (DSS) (Sigma‐Aldrich). We used a PICK1 PDZ‐DAT C10 concentration of 200 μM. For the competition binding experiments, we added Tat‐C5 peptide to an equimolar concentration of 200 μM. For the PreScission protease (GE Healthcare)‐induced competition binding experiments, we subsequently added 10 U (10 U/ml). In both cases, we monitored the change in amide chemical shifts by sequential acquisition of ^1^H‐^15^N HSQC spectra recorded using 8 scans and 128 increments.

The backbone chemical shifts for the construct were obtained by importing the peak list from the previous published NMR structure of the PICK1‐PDZ‐DAT C10 (Erlendsson *et al*, 2014) construct (BMRB entry: 18522, PDB ENTRY:2LUI). The imported peak list was adapted manually in CCPN analysis 2.4.1. The peak list were copied and adapted for each individual spectrum, and the chemical shift perturbation was quantified as the Euclidian distance (Δδ_HN_ = √(0.5([δ_H_^2^+(α(δ_N_^2^)])) between the center position of the original peak (before cleavage and addition of Tat‐C5) and the position of the resulting peak after addition of Tat‐C5 or Tat‐C5 and C3 protease, respectively. Where Δδ_HN_ is the change in chemical shift in ppm, δ_H_ is the change in position of the peak in the 1H dimension, δ_N_ is the change in position of the peak in the ^15^N dimension, and α is a scaling factor between the two dimensions; in this case, 0.153 was used. The active peaks in the exchange between DATC10 and Tat‐C5 were selected as being the residues with a chemical shift perturbation greater than two times the standard deviation of the mean chemical shift perturbation (2σ = 0.077).

The HADDOCK (High Ambiguity Driven protein–protein Docking) (van Zundert *et al*, 2016) algorithm was used to dock Tat‐C5 into the PDZ domain of PICK1. Only, the chemical shift perturbation obtained was used as restraints in the docking. For the HADDOCK modeling, the following residues were chosen as active: I33,G34, I35, S36, I37, G40, C44, C46, L47, Y48, I49, Q51, G67, D68, E69, K83, V86, K103, L104, and the algorithm was provided with a the lowest energy structure of PICK1‐PDZ‐DAT C10 (PDB: 2LUI) where the DATC10 was removed prior to docking (final residues: 23‐107). In Tat‐C5 the residues, Y1, R3, R6, R7, Q8, H12, W13, L14, K15, and V16 were chosen as active residues. A total of 1,000 structures were initially docked, and the top 200 models were refined further using simulated annealing followed by water refinement. The best cluster of structures contained 42 structures, with a mean HADDOCK score of −159.8 ± 7.5 arbitrary units with an overall RMSD of 0.8 ± 0.4 Å.

### HEK293‐GT cultures and transfection

Human Embryo Kidney 293 GripTite cells (Thermo Fisher, catalog number: A14150) (HEK293‐GT) for SCMS were grown in 75‐cm^2^ flasks until 70% confluent in Dulbecco's modified Eagle's medium 1965 with fetal calf serum and pen‐strep antibiotics (DMEM 1965 + +). Cells were transfected with Tac‐YFP‐DAT C24 (pEYFP‐C1 vector) (Erlendsson *et al*, 2019) using lipofectamine in opti‐MEM^®^ (Invitrogen) overnight. Cells were washed in PBS and detached using 0.5% Trypsin with EDTA (Sigma‐Aldrich). Cells were counted in a Countess FL Automated Cell Counter (Thermo Fisher) using Trypan blue (Sigma‐Aldrich). Cells were seeded with a density of 200,000 cells pr. ml in a 6‐well plate. Cells were then grown overnight at 37°C in a humidified 10% CO_2_ atmosphere.

### Supported cell membrane sheets

The SCMS was prepared as described in Erlendsson *et al* (2019). In brief, round (Ø25 mm) coverslips (VWR 631‐1346) were plasma cleaned (Harrick plasma cleaner) and coated with 0.3 mM poly‐L‐ornithine hydrobromide (Poly‐ORN Sigma‐Aldrich) for 30 min. Poly‐ORN was washed out with 2 ml MilliQ water. The 6‐well plates containing the cells expressing Tac‐YFP‐DAT C24 were washed twice in MilliQ for a total of 1 min, to allow cells to swell. The swelled cells were covered with a cover glass with the Poly‐ORN‐coated side facing down. Dynamic pressure was manually applied to the cover glass using the piston from a 12‐ml plastic syringe for a total of 1 min. subsequent removal of the cover glass caused cell rapture leaving SCMS on the surface of the cover glass. The cover glass was covered in sheet buffer (10 mM HEPES, 120 mM KCl, 2 mM MgCl_2_, 0.1 mM CaCl_2_, and 30 mM glucose at pH 7.35) supplemented with 1 mg/ml BSA to block unbound Poly‐ORN for 20 min on ice (in the dark). Coverslips were washed 3 times with sheet buffer. Protein solution (freshly prepared) was added and incubated according to the experiment. In the experiments using a premixed PICK1:peptide solution, 100 nM DY549‐SNAP‐PICK1 was incubated for at least 20 min with different concentrations of C5, Tat‐C5, or Tat‐P_4_‐(C5)_2_ before addition to the cover glass. The protein:peptide solution incubated with the SCMS‐cover glass for 2 h in the dark on ice. In the case of pre‐binding of SCMS with PICK1, the SCMS was incubated with 400 nM of DY549‐SNAP‐PICK1 for 1 h on ice in the dark. Unbound protein was washed away with sheet buffer, and peptide solutions were added and incubated for 2 h in the dark on ice. The SCMS coverslips were then washed in sheet buffer and twice in PBS. The SCMS was fixed for 40 min in 4% PFA, washed 3× in PBS, and mounted onto objective glasses using Prolong Gold Antifade™ mounting medium (Life Science Technologies).

### SAXS

Purified recombinant PICK1 (40 μM) was incubated with Tat‐P_4_‐(C5)_2_ (20 μM). Precipitates were removed by centrifugation (5 min, at 16,100 *g* at 4°C). Sample composition was verified by size‐exclusion chromatography (Superdex200, 10/300, 24 ml) to be homogeneous, and fractions were collected, pooled, and concentrated using a 10 kDa cutoff spin filter (Millipore) to a final concentration of 2.45 mg/ml. The PICK1 samples was prepared by isolating the dimer peak by collecting fractions from a HiLoad Superdex200 PG 16/600 (120 ml) and concentrating the dimer fractions to 3 mg/ml using a 10 kDa cutoff spin filter. Concentration series was prepared for both PICK1 and PICK1:Tat‐P_4_‐(C5)_2_, ranging from 0.5 mg/ml to 3 mg/ml and 0.5 mg/ml to 2.45 mg/ml, respectively. Samples were transported in an ice bath to the Deutsche Electron Synchrotron (DESY) facility, Hamburg, Germany. SAXS experiments (Appendix Table S3) were conducted at the DESY, Hamburg, Germany, using the P12 SAXS Beamline at the PetraIII storage ring (Blanchet *et al*, 2015). Preliminary data reduction includes radial averaging and conversion of the data into absolute scaled scattering intensity, *I(q),* as a function of the scattering vector *q,* where *q = (4π* sin*θ)/λ* (where *θ* is the half scattering angle) were done using the standard procedures at the beamline (Blanchet *et al*, 2015). As part of the process, the individual buffer and sample frames were looked through manually to check for radiation damage. The frames where indications of radiation damage were observed were removed, and the data averaging was done manually for the remaining files. For PICK1:Tat‐P_4_‐(C5)_2_, data merging was done by merging the low *q*‐range data [0.01045–0.04036] Å^−1^ from the 0.5 mg/ml sample together with the high q‐range [0.03237–0.2694] Å^−1^ data from the 2.45 mg/ml sample, with an overlap in the [0.03237–0.04036] Å^−1^ *q*‐range. The scattering data were merged, and buffer subtracted curves analyzed by Guinier analysis using the inbuilt analysis tools of the ATSAS program package (Petoukhov *et al*, 2012). The p(r) functions were plotted using http://bayesrelax.org/test/ with default settings. The shown data were binned using WillItRebin with default settings for clarity. Ensemble optimization method (EOM) (Bernadó *et al*, 2007; Tria *et al*, 2015) was used to fit the merged data using the all atom relaxed MD model of the dimeric PICK1 BAR domain from Karlsen *et al* (2015) together with the lowest energy structure of the PICK1 PDZ domain (PDB:2LUI) as fixed domains in the ensemble. The unstructured C‐terminal, N‐terminal, and PDZ:BAR linker were modeled using fully flexible dummy atoms and allowed for flexibility in the relative orientation of the domains. Tetrameric BAR domain models were created using PyMOL by duplicating, translating, and rotating the dimeric BAR domain into suggested conformations of the PICK1 tetramer (Karlsen *et al*, 2015). EOM (Ranch) (Bernadó *et al*, 2007; Tria *et al*, 2015) was used to generate 10,000 models for each BAR domain configuration with 15 harmonics. The models were fitted with either complete asymmetry or with 10% symmetric structures (in this case the 10% symmetric structures). EOM (Gajoe) (Bernadó *et al*, 2007; Tria *et al*, 2015) was used to fit the merged dataset to single pool (10,000 models) and multi‐pool ensembles. Gajoe was run using 1,000 generations in the genetic algorithm with 100 ensembles, a non‐fixed ensemble size, with a maximum of 20 curves pr. ensemble and 100 repetitions. Fits were evaluated on the basis of their χ^2^ value and their *D*_*max*_ and *R*_*g*_ distributions.

### Primary cultures of rat hippocampal neurons

Hippocampal neurons were prepared from prenatal E19 Wistar rat pups (mixed gender). Brains were isolated from 6 to 8 rat embryos, and brains were dissected in ice‐cold dissection medium (HBSS (Gibco) supplemented with 30 mM glucose (Sigma‐Aldrich), 10 mM HEPES (Gibco) pH 7.4, 1 mM sodium pyruvate (Gibco), 100 μg/ml penicillin, 100 μg/ml streptomycin (Sigma‐Aldrich)). The cerebellum and meninges were removed, and hippocampi from both hemispheres were dissected out. The hippocampi were treated with papain (20 units/ml dissection medium, Worthington) for 20 min at 37°C, triturated (2 × 10 times) using differentially fire‐polished Pasteur pipettes, and filtered through a 70‐μM cell strainer. Dissociated neurons were seeded at a density of 50,000–100,000 cells/coverslip on poly‐L‐lysine (Sigma‐Aldrich)‐coated 25‐mm glass coverslips emerged in Neurobasal medium (Gibco 21103‐049) (supplemented with 2% (vol/vol) glutamax (Gibco 35050‐038), 1% pen/strep, 2% (vol/vol) B27 (Gibco 7504‐044), and 4% FBS (Gibco 7504‐044)). Cell cultures were grown in a 37 °C, 5% CO_2_, and 95% humidified atmosphere. After 24 h, the growth medium was substituted with serum‐free medium, and cells were grown for 21 days, with addition of fresh growth medium every 3–4 days.

### Transduction of hippocampal neurons

At 14 days *in vitro* (DIV), lentiviral constructs packed with FUGWH1sh18eGFP (GFP‐sh18) or FUGWH1sh18deleGFP (GFP) were added to each well. FUGWH1sh18eGFP expresses a short hairpin (sh18) that targets endogenous PICK1 and a shRNA‐resistant eGFP‐tagged PICK1, while the FUGWH1sh18deleGFP did not contain sh18 (Holst *et al*, 2013; Jensen, 2017). Lentiviruses were produced as described previously (Rasmussen *et al*, 2009).

### Immunocytochemistry on hippocampal neurons

21 DIV hippocampal neurons were incubated with 5 μM TMR‐Tat‐P_4_‐(C5)_2_ or TMR‐Tat‐C5 or TMR‐C5 for 1 h in conditioned media at 37°C, rinsed 3 times in PBS, and incubated with 5 μM of the membrane dye DiO (Thermo Fisher) for 10 min at room temperature. After an additional 3 washes in PBS, the hippocampal neurons were fixed in 4% PFA + 4% sucrose for 10 min at room temperature and subsequently washed three times in PBS and briefly in MilliQ. The coverslips were mounted by using Prolong Gold Antifade™ mounting medium (Life Science Technologies).

#### Permeability studies

20‐22 DIV hippocampal neurons were incubated with 5 nM of TMR‐Tat‐P_4_‐(C5)_2_ for 1 h at 37°C, rinsed 3 times in PBS, and fixed in 4% PFA + 4% sucrose for 20 min (10 min on ice and 10 min at room temperature), rinsed again in PBS, permeabilized, and blocked in 0.05% Triton X‐100 and 5% goat serum for 20 min at room temperature, labeled with primary GFP antibody for 1 h at room temperature, and followed by staining with goat anti‐rabbit Alexa‐488. After three final washes with PBS, the coverslips were mounted by using Prolong Gold Antifade™ mounting medium (Life Science Technologies). Quantification of the TMR‐Tat‐P_4_‐(C5)_2_ peptide penetration was carried out by ImageJ software. Images (16 bit) were masked based on the 488‐channel; the mask threshold was set at 1059 to 65535 and the background of the TMR‐Tat‐P_4_‐(C5)_2_ at 726. The TMR‐Tat‐P_4_‐(C5)_2_ peptide mean intensity within the mask was calculated for individual neurons, and each mean was divided by the corresponding masked area.

### Hippocampal neurons feeding assay

Live hippocampal neurons at 21 DIV were incubated with 20 μM Tat‐P_4_‐(C5)_2_ or Tat‐C5 or vehicle for 1 h before internalization in conditioned medium with 2 μM TTX and 100 μg/ml leupeptin at 37°C. Surface AMPA receptors were labeled with the antibody anti‐GluA2 (10 μg/ml) for the last 15 min. After 2 washes in PBS, neurons were treated with 1 μM PMA (Sigma‐Aldrich) in conditioned media (with or without Tat‐P_4_‐(C5)_2_, Tat‐(C5)) for 10 min. Neurons were fixed in 4% PFA + 4% sucrose for 5 min at room temperature, blocked with 10% goat serum for 15 min, and stained with anti‐mouse Alexa‐488 secondary antibody (5 μg/ml) in 3% goat serum for 45 min. Following fixation for 15 min, cells were permeabilized in 0.2% TX‐100 for 5 min and stained with anti‐mouse Alexa‐647 secondary antibody (1 μg/ml) in 3% goat serum for 45 min. The slides were washed three times in PBS and once in MilliQ and subsequently mounted to coverslips as described earlier. After outlining individual neurons on the basis of surface signal (488 nm), the mean intensity of the two channels was determined and the signal ratio (647 nm (internalized)/488 nm (surface)) was used as measure of internalization. A MatLab script was generated to automatically calculate overall intensity ratio for each neuron.

### Imaging

Imaging of SCMS, HEK293 cells, and hippocampal neurons was done on a Zeiss LSM 510 confocal laser‐scanning microscope, equipped with an oil immersion objective, numerical aperture 1.4, 63× (Zeiss). For excitation of YFP, a 488 nm argon‐krypton laser was used, with detection using a 505–550 nm bandpass emission filter. Red dyes were excited using a 543 nm helium‐neon laser, and emission was detected using a 560–615 nm emission filter. Far red fluorophores were excited using a 633 nm laser light from a helium‐neon (HeNe) laser source, and emitted fluorescent light was filtered using a long pass filter.

Images were acquired at 1,024 × 1,024 pixels, 8‐bit pr. pixel, with 4‐, 8‐, or 16‐line averaging scans. Images were treated using ImageJ or Zen (Zeiss software).

### Animals

SNI experiments were conducted on adult wild‐type male mice (10–15 weeks old at initiation of experiment) from Taconic Biosciences, Denmark (C57BL/6JBomTac), unless otherwise stated. Mice were left to habituate for at least 2 weeks before starting the experiment.

Mice were housed with littermates in a temperature‐controlled room maintained on a 12:12 light:dark cycle (tests performed during light phase) and allowed access to standard rodent chow Altromin1342 (Brogaarden, Denmark) and water *ad libitum*.

All experiments were approved by the Animal Experiments Inspectorate under the Danish Ministry of Food, Agriculture and Fisheries, and all procedures adhered to the European guidelines for the care and use of laboratory animals, EU directive 2010/63/EU. All efforts were made to minimize stress or discomfort as well as the number of animals used in each experiment.

### Co‐immunoprecipitation

#### Acute hippocampal slices and lysates preparation

Acute hippocampal brain slices were prepared from adult male C57BL/6 mice (8–16 weeks old). The hippocampi were quickly dissected and sliced (400‐μm transverse sections) into ice‐cold aCSF buffer (124 mM NaCl, 3 mM KCl, 26 mM NaHCO_3_, 1.25 mM NaH_2_PO_4_, 1 mM MgSO_4_, 2 mM CaCl_2,_ and 10 mM D‐glucose) and placed in carboxygenated aCSF for 1 h for recovery. Slices were then incubated with 20 μM Tat‐P_4_‐(C5)_2_ or Tat‐C5 for 1 h followed by 20 μM NMDA for 3 min for chemical LTD induction, in aCSF buffer. Hippocampal slices were lysed in lysis buffer (50 mM Tris, 150 Mm NaCl, 0.1% SDS, 0.5% NaDeoxycholate, 1% Triton X‐100, 5 mM NaF, and 1× Roche protease inhibitor cocktail at pH 7.4), and protein was incubated overnight at 4°C with 5 μg of antibody (either anti‐PICK1 or anti‐GluA2). Protein G agarose bead slurry was added for 3 h, and the beads were then washed 3 times in lysis buffer. Proteins were eluted in 2× Laemmli sample buffer at 100°C for 6 min before Western blotting.

#### Spinal cord lumbar tract total lysates preparation

Spinal cord lysates were prepared from 10‐week‐old C57BL/6 mice. The animals were injected i.t. with 20 μM Tat‐P_4_‐(C5)_2_ or Tat‐C5 and sacrificed 1 h post‐injection. The spinal cords were dissected in ice‐cold PBS1X by hydraulic extrusion according to the procedure described in Richner *et al* (2017). The lumbar tracts of the spinal cords were quickly harvested and lysed in lysis buffer. The same procedures described for hippocampal slices were used to produce spinal cord lysates.

#### Transfected HEK293 cells total lysates preparation

The same procedures described for hippocampal slices were used to produce HEK cell lysates.

### Surface biotinylation

Spinal cords were extruded from 8‐ to 12‐week‐old naïve and SNI mice and lumbar tract was dissected as in Richner *et al* (2011), and 400‐μM transverse slices were generated using a McIlwain tissue chopper. Slices were then placed and separated in ice‐cold aCSF (124 mM NaCl, 3 mM KCl, 26 mM NaHCO_3_, 1.25 mM NaH_2_PO_4_, 1 mM MgSO_4_, 2 mM CaCl_2,_ and 10 mM D‐glucose) before recovery in carboxygenated aCSF for 1 h. Slices were incubated for 1 h with 20 μM peptide inhibitor and subsequently incubated in 1 mg/ml biotin (EZ‐link sulfo‐NHS‐SS‐Biotin: Thermo #21331) in ice‐cold carboxygenated aCSF for 45 min, washed 3 times in 10 mM glycine, 1 time in Tris‐buffer saline (TBS) (20 mM Tris, 150 mM NaCl, pH 7.6), homogenized in lysis buffer (25 mM Tris, 150 mM NaCl, 1% Triton X‐100, 0.5% NaDeoxycholate, 0.1% SDS, 1 mM EDTA, 2 mM Naf, and 1 Roche protease inhibitor, pH 7.6) and centrifuged at 20,000 *g* for 15 min. 500 μg of the supernatant was incubated with Streptavidin dynabeads (Life Technologies: 65601) for 2 h. Bead complexes were washed 3 times in lysis buffer and 1 time in TBS. Proteins were eluted in 2× Laemmli sample buffer at 100°C for 8 min before Western blotting.

### Spared nerve injury surgical procedure

Spared nerve injury (SNI) model was performed according to methods described in Richner *et al* (2011). Briefly, under isoflurane (2%) anesthesia, the skin on the lateral surface of the left thigh was incised and the biceps femoris muscle was divided lengthwise to expose the three branches of the sciatic nerve. The common peroneal and tibial branches were tightly ligated and axotomized distally to the ligation, while the sural branch was left intact. Animals were monitored daily for signs of stress or discomfort but in all cases recovered uneventfully.

### Drug preparation and administration

For intrathecal (i.t.) drug administration, 7 μl was injected into isoflurane anesthetized mice with a 10 μl Hamilton syringe (30 G) in the intervertebral space between L4/L5 or L5/L6 at an angle of 30–45° eliciting a Straub tail response as described previously (Hylden & Wilcox, 1980). For intraperitoneal (i.p.) injections, 10 μl/g per mouse was administered with a 27 G needle. Drugs were diluted in 0.9% isotonic saline for both i.t. and i.p. injections.

### Behavioral experiments

Von Frey testing of mechanical allodynia was performed as previously described (Richner *et al*, 2011). In short, mice were placed in red plastic cylinders on a wire mesh (15‐min habituation prior to testing). von Frey filaments (0.02–1.4 g) were applied in ascending order to the lateral part of the hind paws. Each von Frey filament was applied five times and a positive response in at least three out of five stimuli determined the threshold level. A positive response was defined as sudden paw withdrawal, flinching, and/or paw licking induced by the filament. Testing was performed blinded toward the nature of injected substance and by the same female researcher to avoid person‐to‐person variation and gender bias.

The paw withdrawal threshold (PWT) was estimated by using the following formula:

$$\begin{array}{cc} \mathrm{PWT} & =\frac{\text{Number of response failures}}{\text{Total number of trials}}\times (\left(\text{filament A}+1gr\right)\\ & \;-(\text{filament A}gr))+\text{filament A}gr \end{array}$$

### Fluorescence peptide administration

10‐week‐old SNI male mice were injected with 20 μM Tat‐P_4_‐(C5)_2_ i.t. and transcardially perfused with cold 0.1 M phosphate buffer (PBS) at time 0, 30, 60, and 120 min post‐injection. *Naïve* animals were subjected to the same procedure after i.t. injection of 20 μM Tat‐P_4_‐(C5)_2_ or Tat‐C5 and perfused 60 min post‐injection. All spinal cords were isolated by hydraulic extrusion and post‐fixed in PFA 4% for 8 h. Spinal cords were subsequently cryoprotected in 30% sucrose, embedded in Tissue‐Tek^®^ OCT, and sliced at 20 μm thickness by Cryostat Leica CM3050 S.

### Immunohistochemistry

Mounted slides were dried at room temperature, rinsed in PBS1X twice for 5 min each, and incubated in blocking buffer containing 5% goat serum, 1% BSA, 0.3% Triton X‐100 in 1× PBS, for 90 min at room temperature. The slices were incubated in blocking buffer with primary antibody overnight at 4°C. On the second day, sections were rinsed 3 times in washing buffer (0.25% BSA, 0.1% Triton X‐100 in PBS) 10 min each. Secondary antibodies Alexa‐488 or Alexa‐647 were diluted in washing buffer and the slides incubated 45 min at room temperature. After one wash in washing buffer and additional washing steps in 1× PBS, sections were air‐dried and mounted with DAPI Fluoromount‐G (Southern Biotech) mounting media.

Regarding the tissue distribution of Tat‐P_4_‐(C5)_2_ or Tat‐C5, the slides were washed 5 min in PBS, air‐dried, and mounted with DAPI Fluoromount‐G mounting media.

### Epifluorescence microscopy

Epifluorescence microscopy was performed using a Zeiss axioscan.Z1, with a plan‐apochromat 10×/0.45 objective (Carl Zeiss). LED light sources were used as follows, DAPI (353‐nm excitation, 50‐ms exposure, 460/70‐nm bandpass filter), Alexa‐488 (493‐nm excitation, 50‐ms exposure, 525/50‐nm bandpass filter), TMR‐568 (577‐nm excitation, 100‐ to 300‐ms exposure, 600/70‐nm bandpass filter), and Alexa‐647 (653‐nm excitation, 200‐ms exposure, 690/50‐nm bandpass filter). All channels were imaged covering a total of around 6 μm of the 20‐μm‐thick spinal cord slices.

### Kainate‐induced cobalt uptake

Postnatal day 14 (P14), C57BL/6 mouse pups of both genders were decapitated and their spinal cord was dissected out in ice‐cold oxygenated stabilization buffer (139 mM sucrose, 32.5 mM NaCl, 2.5 mM KCl, 10 mM MgSO_4_, 12 mM d‐glucose, 24 mM NaHCO_3_, 1 mM NaH_2_PO_4_, 0.5 mM CaCl_2_). The lumbar part was embedded with cyanoacrylate in a 5% agar block, and 400‐μm slices were cut on a vibrating microtome (Thermo Scientific Microm HM 650 V). The slices were divided into three groups and left to recover in oxygenated stabilization buffer at RT for minimum 60 min. Tissue from each animal was subjected to all three experimental groups. Slices were pre‐incubated with 20 μM Tat‐P_4_‐(C5)_2_ (as indicated) in oxygenated stabilization buffer for the last 30 min of incubation in stabilization buffer. Slices were then transferred to oxygenated uptake buffer (139 mM sucrose, 57.5 mM NaCl, 5 mM KCl, 2 mM MgCl_2_, 1 mM CaCl_2_, 10 mM HEPES, 12 mM D‐glucose) followed by addition of 20 μM Tat‐P_4_‐(C5)_2_ and 20 nM TNFα (as indicated) for 10 min. Co^2+^ and kainate were added to a final concentration of 2 mM Co^2+^ and 200 μM kainate for 20 min to all three experimental groups. Cobalt loading was terminated by 2 × 5 min wash in uptake buffer containing 3.5 mM EDTA followed by treatment with 0.12% (NH_4_)_2_S for 6 min to precipitate intracellular Co^2+^. Slices were rinsed in PBS and fixed in 4% PFA for 3 h at 4°C followed by cryoprotection in 30% sucrose o/n at 4°C. Slices were rinsed in PBS, embedded in OCT, and cut to 25‐μm slices on cryostat. Sections were processed to enhance the CoS reaction by silver intensification as described in Engelman *et al* (1999). Due to non‐specific Co^2+^ labeling in the outer parts of the 400‐μm slices, these were excluded for analysis. Average cell count of 2–4 of the 25‐μm slices from each 400‐μm slice was used for quantification. Images were acquired using a Zeiss Widefield microscope using a 20× objective. For quantification, pictures were captured using identical exposure settings. Pictures were imported into ImageJ (version 2.0.0‐rc‐41/1.50b), and cells with black soma were counted manually. Analysis and counting were performed blinded by investigators.

### *In vivo* electrophysiology

Electrophysiological recordings were performed *in vivo* in adult decerebrate mice. The preparation has been previously described extensively (Meehan *et al*, 2012, 2017). Briefly, the initial surgery was performed under isoflurane anesthesia (1.5–2%), including the insertion of tracheal cannula for subsequent artificial ventilation, the dissection of the sural nerve (on the injured side), and a dorsal hemi‐laminectomy between vertebrae levels T12‐L1 (exposing spinal levels L3‐L4). Mice were then placed in a modified Narishige frame and attached to a ventilator (CWE, 72 breaths/min). Craniotomy was performed, superficial blood vessels cauterized, and the entire brain rostral to the superior colliculus removed. The void was packed with surgical to prevent bleeding. After approximately 10 min, this was removed to confirm complete decerebration before being replaced followed by removal of isoflurane from the ventilation flow. Throughout the experiment, the temperature of the animals was kept stable at approximately 37°C using a heat lamp and heat blanket thermostatically controlled by a rectal thermometer. Expired pCO_2_ was also stable throughout the experiments.

The dissected peripheral nerve was hooked on a custom‐made bipolar stimulating electrode, and the spinal cord mechanically stabilized using vertebral clamps above and below the segments of interest. A silver ball electrode was placed on the lateral edge of the laminectomy, and a reference electrode placed down the side of the ribcage on the ipsilateral side. This recorded the cord dorsum potential that shows the incoming volley of sensory input in the dorsal roots.

The peripheral nerve was stimulated at thresholds to ensure maximal stimulation of the high‐threshold Aδ and C fibers (Steffens *et al*, 2012). This electrode was manually moved rostral and caudal to determine the site with the maximal incoming volley. The dura was then removed from the dorsal surface at this region, and this site was used for recordings. A glass microelectrode filled with potassium acetate was inserted in the spinal cord using a motorized micro‐drive, and field potentials were recorded at depths corresponding to laminae 1 and 2 using an Axoclamp 2b amplifier (Axon Instruments). These measurements were made at locations in the lateral gray matter indicated in Fig 7C as these regions have been shown to show maximal field potentials (Schouenborg, 1984).

Both the incoming volley and field potentials were further amplified and filtered using Digitimer (UK) amplifiers, digitized using a Power 1401 digital to analogue converter (CED, UK), recorded, and analyzed using Spike 2 software (CED, UK). All measurements were made from averages of at least 10 successive stimulations (performed in Spike 2). The incoming volley was measured as the peak‐to‐peak amplitude from the first 3 inflections of the cord dorsum potential as illustrated on Fig 7C and D. Baseline recordings were initially performed, followed by application of the peptide to the surface of the spinal cord, and the recordings performed again 20‐30 min later in the same location. In 3 mice, the spinal cord was washed immediately after the second measurements were made and recording of the incoming volley repeated after 1 h and 2 h.

### Antibodies

Western blots, fixed primary hippocampal neurons, HEK 93 cells, and tissue sections were analyzed with primary antibodies directed against the following: GFAP (rabbit polyclonal, Dako Cat. No. Z0334; dilution 1:1,000 for immunohistochemistry), NeuN (mouse monoclonal, Millipore Cat. No. MAB377; dilution 1:100 for immunohistochemistry), PICK1 (rabbit polyclonal JH2906 kindly provided from Richard Huganir's laboratory, 5 μg for immunoprecipitation) and PICK1 (mouse monoclonal clone 2G10 custom generated; dilution 1:500 for Western blot), GFP (rabbit polyclonal, Abcam Cat. No. Ab290; dilution 1:500 for immunocytochemistry and Western blot), GluA2 (mouse monoclonal, Millipore Cat. No. MAB397; dilution 1:100 for immunohistochemistry and 1:1,000 for Western blot), pS880 GluA2 (mouse monoclonal, Millipore Cat. No. MABN103; dilution 1: 500 for Western blot), GluA1 (rabbit monoclonal, Millipore Cat. No. 04‐855; dilution 1:500 for Western blot), Pan‐Cadherin (rabbit polyclonal, Cell Signaling Cat. No. #4068; dilution 1:500 for Western blot), β‐actin HRP‐conjugated (mouse monoclonal, Sigma Cat. No A3854.; dilution 1:10,000 for Western blot), and IgG‐negative controls (normal mouse, Santa Cruz Cat. No. SC‐2025, 5 μg; rabbit polyclonal, Cell Signaling 27295, 5 μg for Western blot).

As secondary antibodies, we used Alexa Fluor 488‐conjugated goat anti‐rabbit IgG (Thermo Fisher scientific, Cat. No. A‐11034; dilution 1:500 for immunohistochemistry and immunocytochemistry), Alexa Fluor 647‐conjugated goat anti‐mouse IgG (Thermo Fisher scientific, Cat. No. A‐21236; dilution 1:500 for immunohistochemistry and immunocytochemistry), Alexa Fluor 488‐conjugated goat anti‐mouse IgG (Thermo Fisher scientific, Cat. No. A‐11029; dilution 1:500 for immunocytochemistry), and (HRP)‐conjugated secondary antibodies for Western blotting (anti‐mouse/anti‐rabbit, Abcam Cat. No. ab99632/ab99697; dilution 1:10,000).

### Statistical analysis

Statistical analyses were performed as indicated specifically for individual experiments. Significance level was set to *P *<* *0.05. GraphPad Prism was used for data analyses. For pharmacological, imaging, and biochemical experiments, sample size was chosen according to common practice with at least 3 independent determinations. More replicates were added in several of the biochemical experiments upon reviewer request. For SNI experiments, mice were randomly assigned to individual experimental groups and von Frey testing was performed blinded toward the nature of injected substance and by the same female researcher to avoid person‐to‐person variation and gender bias. A pre‐specified group size of 8 animals was chosen based on previous experience with pharmacology in the SNI model. In 6B and H, animals were taken down prior to experiment reducing the measured n, but in both cases significance was not compromised. Exception is Fig EV4D, where only 4 animals were used for the positive control gabapentin because of its well‐known effect size.

## Author contributions

Peptides were designed by AB, KS, MRa, and KLM. They were synthesized by AB, MS, and KBN, and plasma stability was assessed by CROB. Binding experiments by FP was done and analyzed by NRC, GNH, and MRa. Binding on SCMSs and FPLC was performed and analyses by NRC. Imaging of shRNA experiments was done by IAJ. All other imaging was performed by MDL with hippocampal cultures prepared by SEP. Co‐IP, phosphorylation, surface biotinylation, and biotin pull‐down were performed by MBL and ABH. Design, recording, and analysis of *in vivo* electrophysiology were done by DBJ and CFM and SNI operations for this done by GNH. *In vivo* behavioral experiments were designed by CBV, MDL, and KLM and carried out and analyzed by MRi, who also performed SNI operations. NMR studies were carried out by SE, NRC, and KT helped analyzing the data. SAXS experiments were carried out by NRC and GNH assisted by SRM, and LA was involved in data analyses and beamtime allocation. ATS, UG, and KLM designed the overall layout of experiments, and MDL, NRC, UG, and KLM wrote the manuscript.

## Conflict of interest

A priority founding patent application (P4997EP00; inhibitors of PICK1 and uses thereof) for Tat‐P_4_‐(C5)_2_ has been filed (KLM, UG, KST, AB, NRC).

## For more information

(i) http://in.ku.dk/research/Madsen-lab
(ii) https://in.ku.dk/research/claire-meehan/
(iii) https://biopharmaceuticals.ku.dk/research/stroemgaard-lab/
(iv) https://www.iasp-pain.org

## Supporting information



Appendix

Expanded View Figures PDF

Review Process File

Source Data for Figure 3

## Data Availability

This study includes no data deposited in external repositories.
