# Supplementary material for: A high‐affinity, bivalent PDZ domain inhibitor complexes PICK1 to alleviate neuropathic pain
Source: EMBO Mol Med. 2020 Apr 30;12(6):e11248. doi: 10.15252/emmm.201911248 (PMC7278562; doi:10.15252/emmm.201911248)
Supplement: Supplementary file 1 — Appendix [file EMMM-12-e11248-s001.pdf]

A high-affinity PDZ domain inhibitor bivalently complexes PICK1 to alleviate neuropathic pain

Appendix Materials

Christensen*, De Luca* et al.

This appendix includes

Appendix Figures S1 to 14

Appendix Tables S1 to 3

Appendix figures and legends

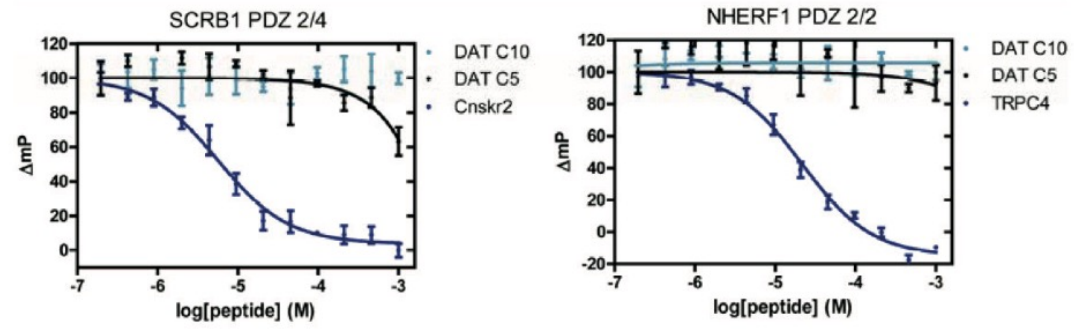

Appendix Figure S1 Fluorescence polarization competition curves for binding of DAT C5 and DAT C10 to SCRB1 PDZ2/4 and NHERF1 PDZ 2/2

SCRB1 PDZ2/4 (left) and NHERF1 PDZ 2/2 (right) were incubation of with fluorescently labelled peptide corresponding to the C-terminus of Cnskr2 and TRPC4, respectively, and competition binding curves constructed by addition of increasing concentration of the non-labelled peptides as indicated by the legends. Whereas Cnskr2 and TRPC4 both bound to their respective PDZ domains, the DAT C-terminal peptides did not bind to either of the domains indicating that the low values observed in the screen most likely represented false positives.

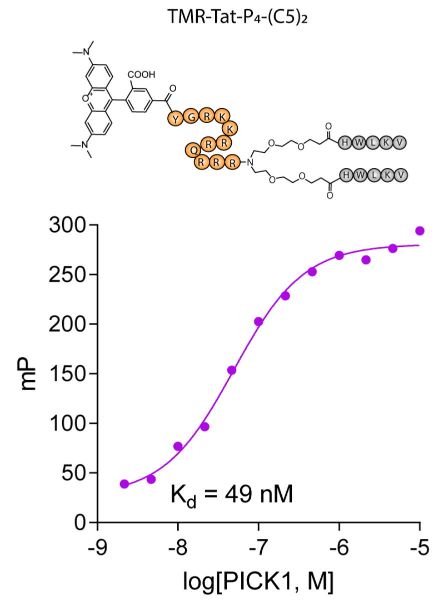

Appendix Figure S2. FP binding curves in competition with TMR-Tat-P_4-$(C5)_2$ as tracer.

Primary sequence of TMR-Tat-P_4-$(C5)_2$ and FP saturation binding curve after titration with increasing concentration of PICK1. Data are shown as mean with error-bars as SEM of n = 3. Binding curves were fitted to a log dose-response (three parameters) extracting the K_d GraphPad Prism 8.3.

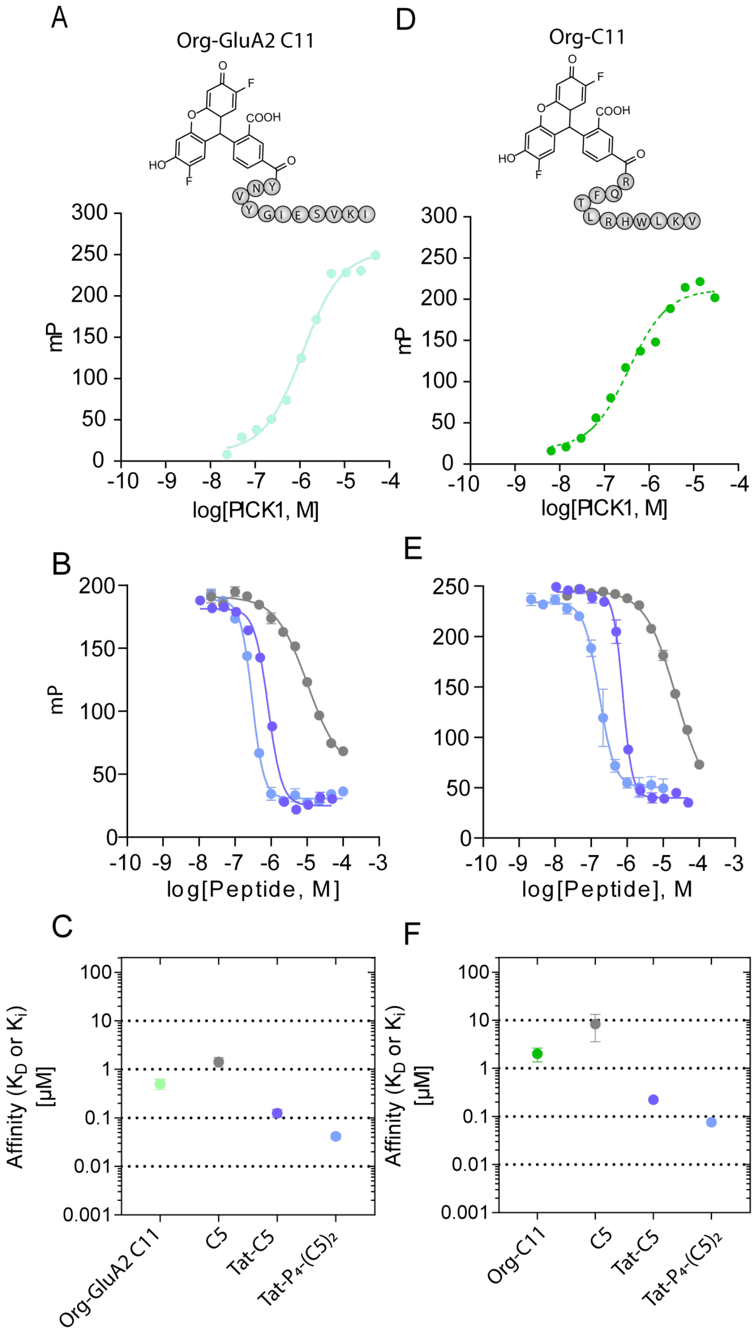

Appendix Figure S3. FP binding curves in competition with different tracers.

A. Primary sequence of Org-GluA2 C11 and FP saturation binding curve after titration with increasing concentration of PICK1.

B. FP competition binding of C5 (*grey*), Tat-C5 (*purple*) or Tat-P_4-$(C5)_2$ (*blue*), to PICK1 with Org-GluA2 C11 (20 nM) as tracer.

C. Summary of affinities derived from fits.

D. Primary sequence of Org-C11 and representative FP saturation binding curve after titration with increasing concentration of PICK1.

E. FP competition binding of C5 (*grey*), Tat-C5 (*purple*) or Tat-P_4-$(C5)_2$ (*blue*), to PICK1 with Org-C11 (20 nM) as tracer.

F. Summary of affinities derived from fits.

Data information: Data are shown as mean with error-bars as SEM of n = 6. Binding curves were fitted to a log dose-response (three parameters) extracting the K_d or IC_{50} using GraphPad Prism 8.3. K_i was calculated using the Cheng-Prusoff equation.

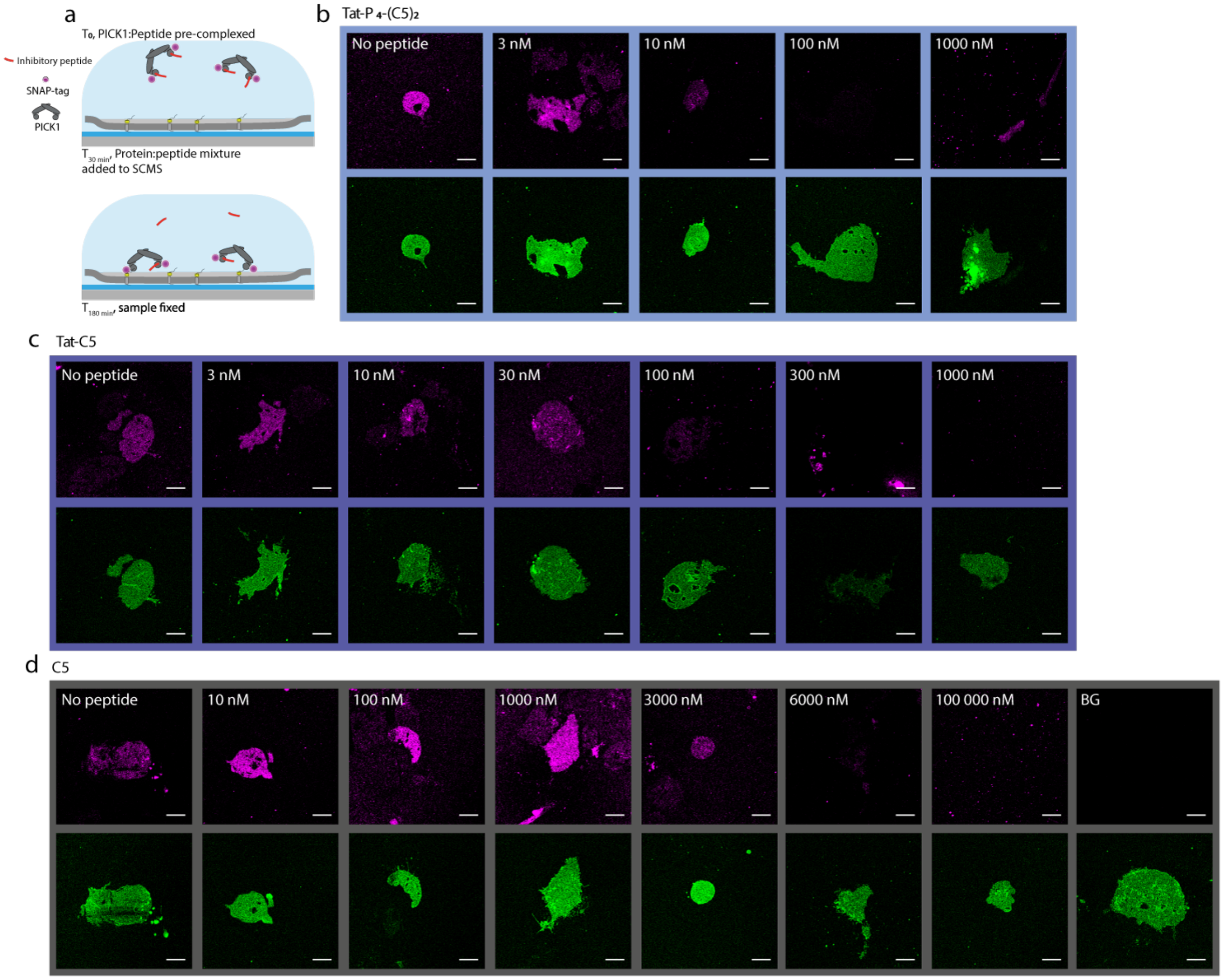

Appendix Figure S4 Competition of PICK1 binding to SCMSs with pre-incubation of inhibitory peptides.

A. Illustration of experimental approach. Fluorescently labeled PICK1 was incubated with inhibitor peptide before interaction with the Supported Cell Membrane Sheets (SCMS) expressing TAC-YFP-DAT C24.

B-D. Representative fluorescence confocal microscopy images of SCMS expressing TAC-YFP-DAT C24 (green), incubated with fluorescently labeled PICK1 (magenta) pre-complexed

with increasing concentration of peptides prior to binding to SCMSs. Visible reduction in binding of PICK1 (magenta) is observed at 10 nM, 100 nM and 3000 nM of Tat-P4-(C5)2, Tat-C5 and C5, respectively. For curves in Figure 1C images were pooled from three independent experiments. Note that images for 10 nM are also shown in Fig 2B.

Data information: In all images scale-bars represents 10 µm.

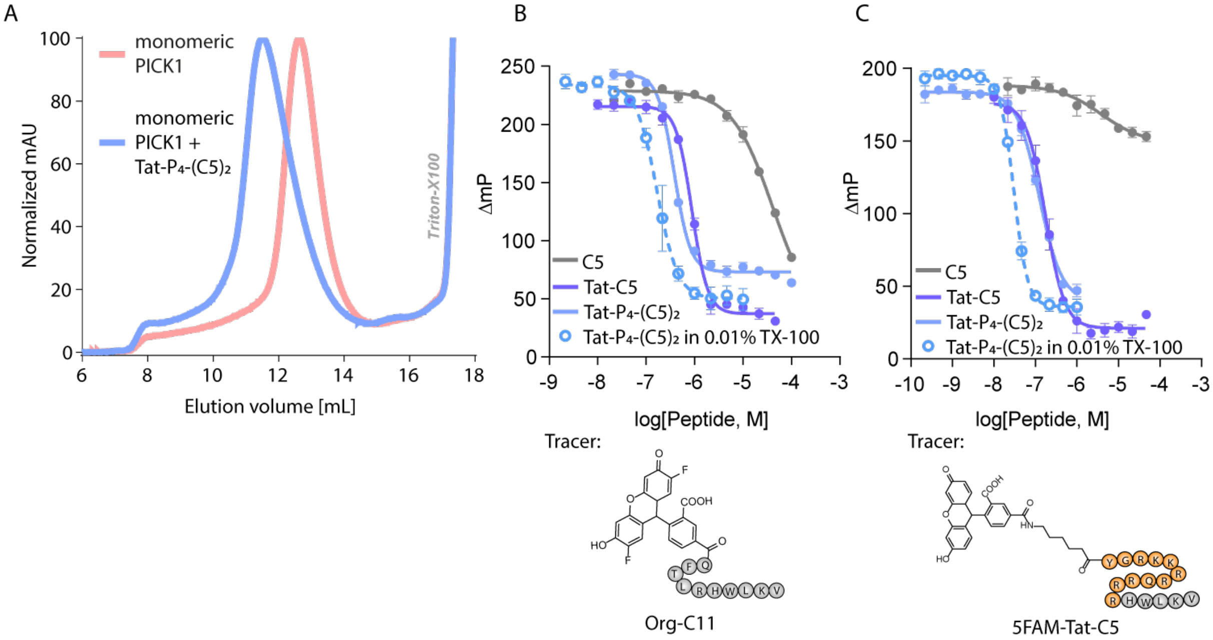

Appendix Figure S5. Tat-P_4-$(C5)_2$ induces a pseudo-dimeric structure of momoneric PICK1.

A. FPLC trace of PICK1 run under monomeric conditions in absence (pink) or presence (blue) of Tat-P_4-$(C5)_2$.

B. FP competition binding of C5, Tat-C5 or Tat-P_4-$(C5)_2$, to monomeric PICK1 with 5FAM-Tat-C5 (20 nM) as tracer. Dashed curve is Tat-P_4-$(C5)_2$ binding to dimeric PICK1

C. FP competition binding of C5, Tat-C5 or Tat-P_4-$(C5)_2$, to monomeric PICK1 with Org-C11 (20 nM) as tracer.

Data information: Data points are shown as mean with error-bars as SEM of n ≥3 replicates. Experiments were repeated for separate purification of PICK1. PICK1 run in a buffer containing 0.1% TX-100 is primarily monomeric, while it's primarily dimeric in a buffer containing 0.01% TX-100.

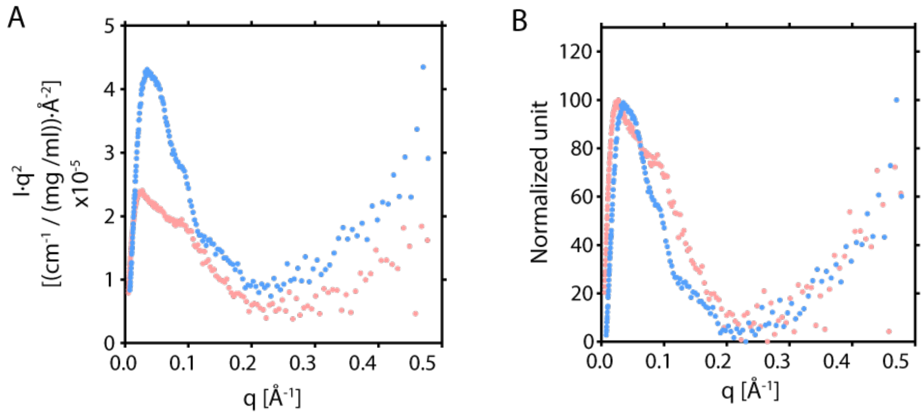

Appendix Figure S6. Kratky plot of SAXS data for PICK1 with and without Tat-P_4-$(C5)_2$.

A. Kratky plot (Iq^2/q) of tetrameric PICK1 complexed with Tat-P_4-$(C5)_2$ (blue) and PICK1 in absence of inhibitor (pink) on absolute scale

B. Kratky plot (Iq^2/q) of tetrameric PICK1 complexed with Tat-P_4-$(C5)_2$ (blue) and PICK1 in absence of inhibitor (pink) normalized to top and bottom (set to 0 and 100, respectively) of the blue curve to facilitate comparison of the shape of the curves.

Data information: The plots show that incubation of PICK with Tat-P_4-$(C5)_2$ (blue) confine the signal slightly more at low q than seen for PICK1 alone (pink) indicating that the peptide reduces inter-domain flexibility.

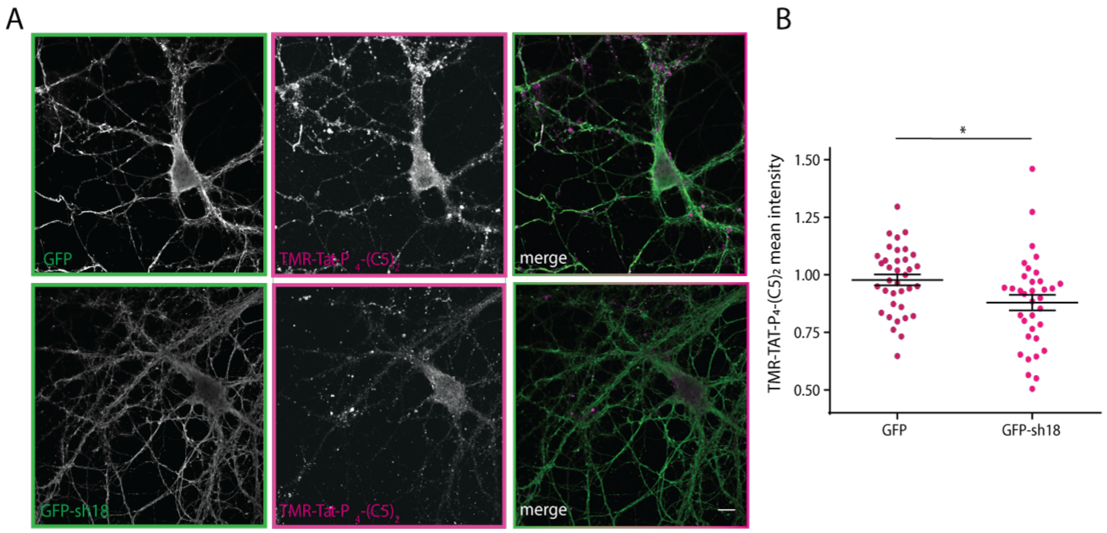

Appendix Figure S7. PICK1 knock down reduces TMR-Tat-P_4-$(C5)_2$ accumulation in hippocampal neurons.

A. Representative confocal images of hippocampal neurons transduced with the viral vector encoding GFP (top) or with the GFP co-expressed with the short hairpin 18 silencing PICK1 (GFP-sh18, middle). Images show: GFP, GFP-sh18 in green, 5 nM of TMR-Tat-P_4-$(C5)_2$ in magenta, as well as the two images merged.

B. Quantification of TMR-Tat-P_4-$(C5)_2$ intensity values of each transduced neuron from A. normalized to the TMR-Tat-P_4-$(C5)_2$ intensity mean of the GFP construct.

Data information: Scale bar: 10 µm. Each dot represents a single neuron, Unpaired t-test, $t_{(69)}$=2,390, *P<0.05, GFP n = 36 and GFP-sh18 n = 35. Horizontal bars show mean with error-bars as SEM.

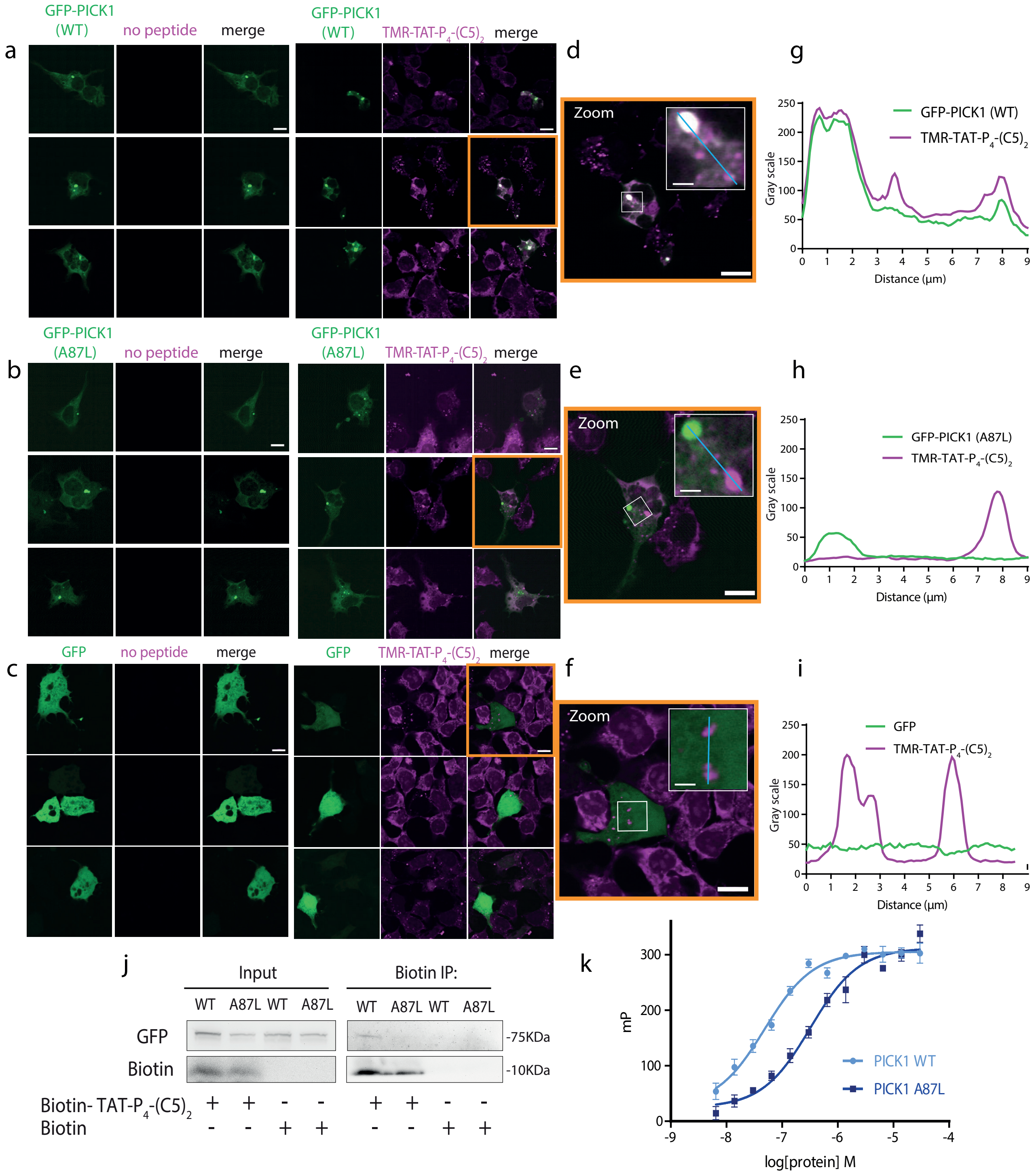
a
GFP-PICK1 (WT)
no peptide
merge
GFP-PICK1 (WT)
TMR-TAT-P4-(C5)2
merge
b
GFP-PICK1 (A87L)
no peptide
merge
GFP-PICK1 (A87L)
TMR-TAT-P4-(C5)2
merge
c
GFP
no peptide
merge
GFP
TMR-TAT-P4-(C5)2
merge
d
Zoom
e
Zoom
f
Zoom
g
GFP-PICK1 (WT)
TMR-TAT-P4-(C5)2
Gray scale
Distance (µm)
h
GFP-PICK1 (A87L)
TMR-TAT-P4-(C5)2
Gray scale
Distance (µm)
i
GFP
TMR-TAT-P4-(C5)2
Gray scale
Distance (µm)
j
Input
Biotin IP:
WT A87L WT A87L
WT A87L WT A87L
GFP
Biotin
-75KDa
-10KDa
Biotin- TAT-P4-(C5)2 + + - - + + - -
Biotin - - + + - - + +
k
mP
log[protein] M
PICK1 WT
PICK1 A87L

Appendix Figure S8. **PDZ-dependent interaction between PICK1 and TMR-Tat-P_4-$(C5)_2$ in heterologous cell.**

A. Representative confocal images of HEK 293 cells overexpressing GFP–PICK1 WT in absence (*left*) or presence (*right*) of TMR-Tat-P_4-$(C5)_2$.

B. Representative confocal images of HEK 293 cells overexpressing GFP–PICK1 A87L (PDZ mutant) in absence (*left*) or presence (*right*) of TMR-Tat-P_4-$(C5)_2$.

C. Representative confocal images of HEK 293 cells overexpressing soluble GFP in absence (*left*) or presence (*right*) of TMR-Tat-P_4-$(C5)_2$

D. Zoom in of merged image highlighted in A. where white indicates collocalization of GFP-PICK1 WT (*green*) and TMR-Tat-P_4-$(C5)_2$ (*magenta*).

E. Zoom in of merged image highlighted in B. Showing no indication of colocalization for GFP-PICK1 A87L (*green*) and TMR-Tat-P_4-$(C5)_2$ (*magenta*).

F. Zoom in of merged image highlighted in B. Showing no indication of colocalization for soluble GFP (*green*) and TMR-Tat-P_4-$(C5)_2$ (*magenta*).

G. Intensity profiles of the line (light blue) from insert zoom D. showing clear colocalization for GFP-PICK1 WT (*green*) and TMR-Tat-P_4-$(C5)_2$ (*magenta*).

H. Intensity profiles of the line (light blue) from insert zoom E. showing no clear colocalization for GFP-PICK1 A87L (*green*) and TMR-Tat-P_4-$(C5)_2$ (*magenta*).

I. Intensity profiles of the line (light blue) from insert zoom F. showing clear colocalization for soluble GFP (*green*) and TMR-Tat-P_4-$(C5)_2$ (*magenta*).

J. Representative immunoblots of pull-down experiment in HEK 293 cells of PICK1 WT or A87L mutant with Biotin-Tat-P_4-$(C5)_2$ or Biotin as negative control.

K. FP saturation binding of (20 nM) TMR-Tat-P_4-$(C5)_2$, to PICK1 WT or PICK1 A87L, showed a decrease in binding capacity of the PICK1 A87L mutant (K_d = 323 nM) compared to PICK1 WT (K_d = 49 nM).

Data information: HEK293 cells shown in A-C. were incubated with 20 μM TMR-Tat-P_4-$(C5)_2$ for 1 h prior to fixation with 4% PFA. Scale bars: 10 μm, inserts 2 μM.

Data points in K. are shown as mean with error-bars as SEM of n = 3. Data in K. was fitted to a log dose-response (three parameters) using GraphPad Prism 8.3.

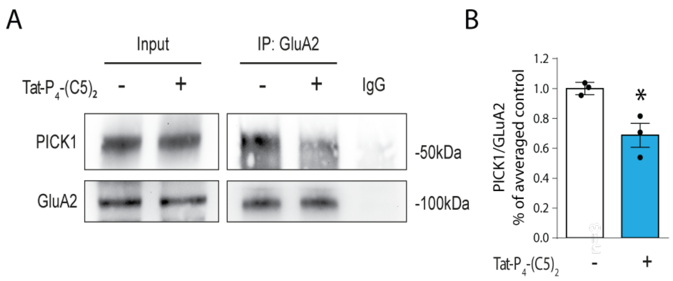

Appendix Figure S9. Tat-P_4-$(C5)_2$ reduces PICK1-GluA2 co-immunoprecipitation *ex vivo*.

A. Representative immunoblots of co-immunoprecipitated PICK1:GluA2 (IP, left panels) from hippocampal slices.

B. Densitometry analysis of immunoblots show a partial disruption of PICK1:GluA2 interaction after 1 hour bath application of 20 µM Tat-P_4-$(C5)_2$ peptide.

Data information: For the pull-down procedure the primary antibody anti-GluA2 has been used instead of anti-PICK1 **(Figure 3C)** in order to validate the procedure. Unpaired t-test, $t_{(4)}$=4,444, *P< 0.05 n=3/group. All data are shown as mean with error-bars as SEM.

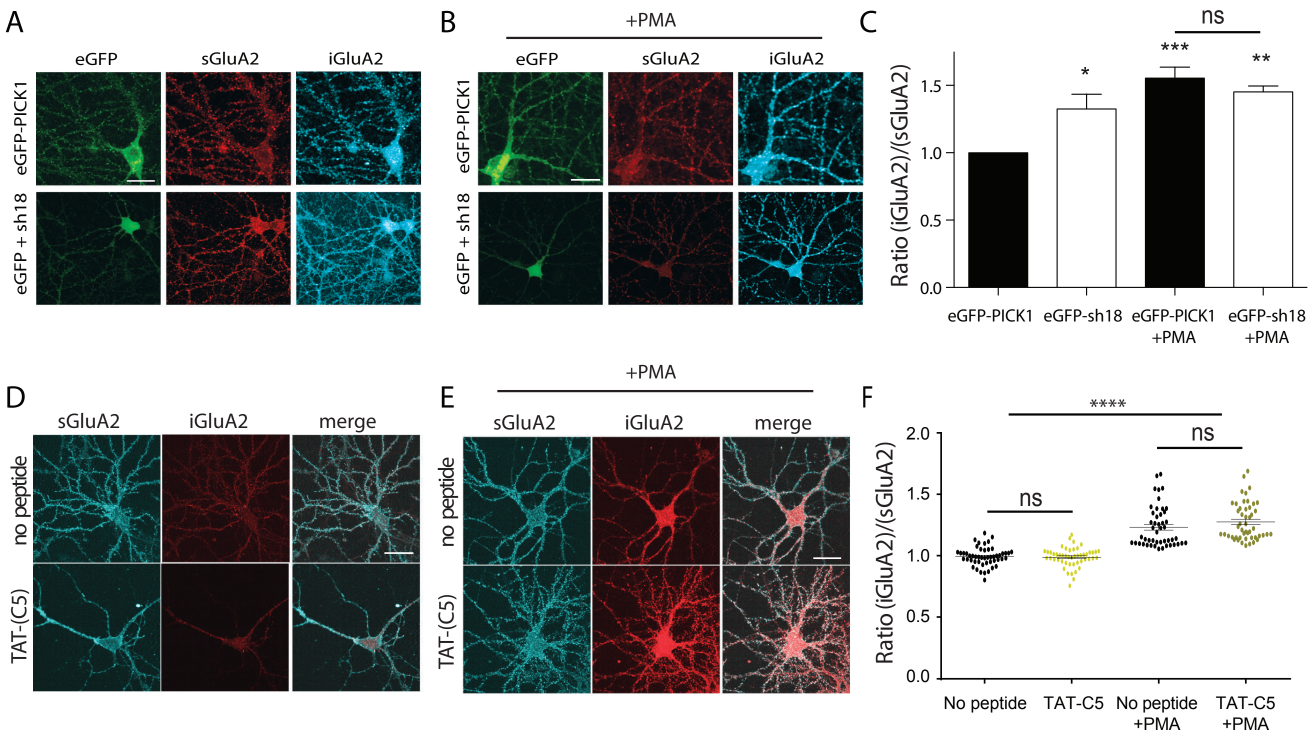

Appendix Figure S10. Knock down of PICK1 increase GluA2 internalization, while Tat-C5 does not

A. Representative confocal images of hippocampal neurons transduced with eGFP-PICK1 (green, top) or GFP co-expressed with the short hairpin 18 silencing PICK1 expression (GFP-sh18, green, bottom), subjected to anti-GluA2 feeding. Under basal conditions knockdown of PICK1 (eGFP-sh18, bottom) induces an increase in internalized GluA2 (iGluA2, cyan) over surface GluA2 (sGluA2, red) ratio.

B. Representative confocal images of hippocampal neurons transduced with eGFP-PICK1 (green,top) or GFP-sh18 (green, bottom), subjected to anti-GluA2 feeding, following PMA stimulation. In this case knockdown of PICK1 (eGFP-sh18, bottom) does not affect the internalized GluA2 (iGluA2, cyan) over surface GluA2 (sGluA2, red) ratio.

C. Quantification of the internalized GluA2 measured as the ratio of internalized/surface labelled GluA2 within the region of interest outlining individual neurons on the basis of surface signal. Statistics were done using a One-way ANOVA with Tukey's post-test * $p \leq 0.05$, ** $p \leq 0.01$, *** $p \leq 0.01$ (n = 9-13 neurons/condition).

D. Representative confocal images of un-transduced hippocampal neurons in absence (top) or presence (bottom) of Tat-C5. Subjected to anti-GluA2 feeding. Under basal conditions neither absence nor presence of Tat-C5 changes internalized GluA2 (iGluA2, cyan) over surface GluA2 (sGluA2, red) ratio. Ordinary two-way ANOVA, followed by Sidak post-test, revealed a significant effect between untreated control and PMA treatment ($F_{(1,192)} = 217.6$ ****p-value ≤ 0.0001), while the effect of Tat-C5 treatment was not significant $F_{(1,192)} = 1.137$, p-value=0.2875) and it did not enter into any interaction (no peptide n=50, Tat-C5 n=49, PMA n=48, Tat-C5 + PMA n=49). All data expressed as mean ± SEM.

E. Representative confocal images of un-transduced PMA stimulated hippocampal neurons in absence (top) or presence (bottom) of Tat-C5. Subjected to anti-GluA2 feeding. Following PMA stimulation neither absence nor presence of Tat-C5 changes internalized GluA2 (iGluA2, cyan) over surface GluA2 (sGluA2, red) ratio.

F. Quantification of the internalized GluA2 measured as the ratio of internalized/surface labelled GluA2 within the region of interest outlining individual neurons on the basis of surface signal.

Data information: In B. and E. neurons were stimulated for 10 min with PMA. In D. and E. 20 µM Tat-C5 was added for 1 hr. Scale-bars represents 30 µm.

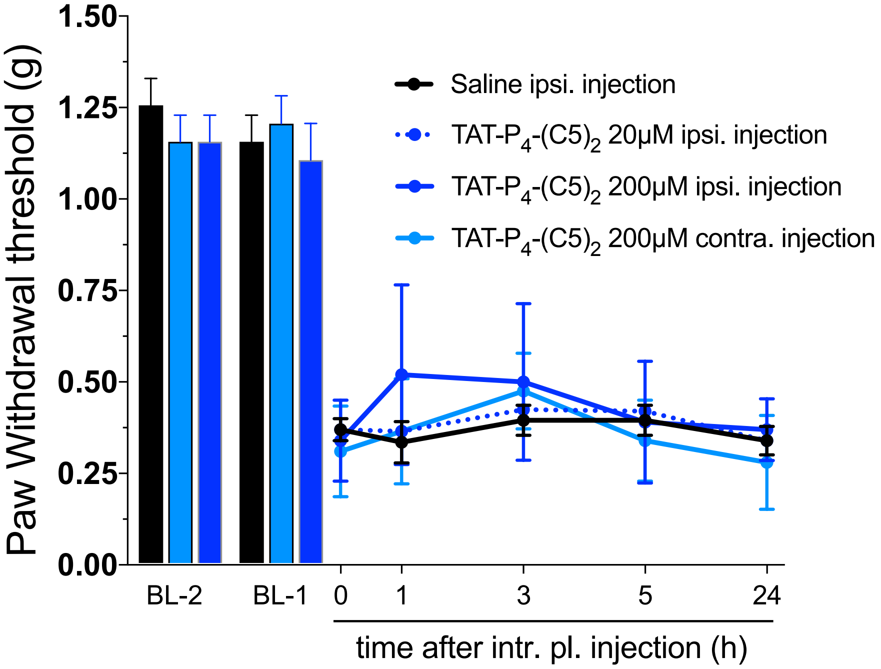

Appendix Figure S11 Tat-P_4-$(C5)_2$ does not alleviate allodynia after intraplantar administration

Intraplatar administration of Tat-P_4-$(C5)_2$ (7µl, 20 µM) or (7µl, 200µM) in the initiation phase of the SNI model did not change the paw withdrawal threshold significantly.

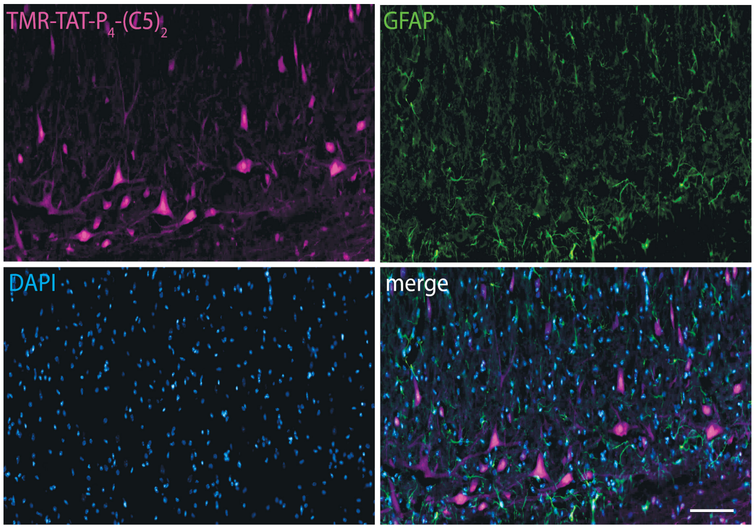

Appendix Figure S12. TMR-Tat-P_4-$(C5)_2$ does not label glia cells.

Sagittal section of the spinal cord lumbar tract shows no overlap between immunostaining of the glial marker GFAP (green) and TMR-Tat-P_4-$(C5)_2$ peptide (magenta) 1 hour after administration i.t (20 μM) in SNI mice. All cell nuclei were stained with DAPI (blue). Scale Bar: 100 μm.

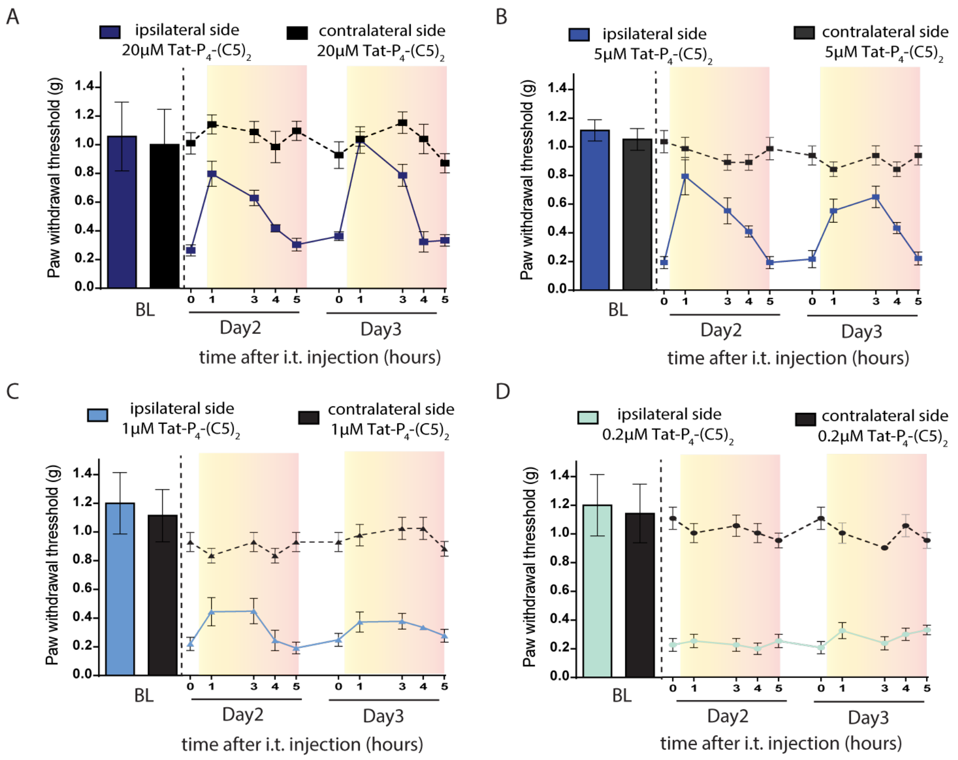

Appendix Figure S13. I.t. administrations of Tat-P$_4$-(C5)$_2$ dose-dependently attenuate mechanical hyperalgesia.

A-D. Acute phase Von Frey test show dose-dependent reduction in SNI-induced hypersensitivity, at 1 and 3 h compared to 0 h, after i.t. administration, of Tat-P$_4$-(C5)$_2$ both at Day 2 and 3 post-surgery. A. 20 µM Tat-P$_4$-(C5)$_2$, n = 7 mice. B. 5 µM Tat-P$_4$-(C5)$_2$, n = 8 mice. C. 1 µM Tat-P$_4$-(C5)$_2$, n = 8 mice. D. 0.2 µM Tat-P$_4$-(C5)$_2$, n= 8 mice.

Data information: All data points are shown as mean with error-bars as SEM.

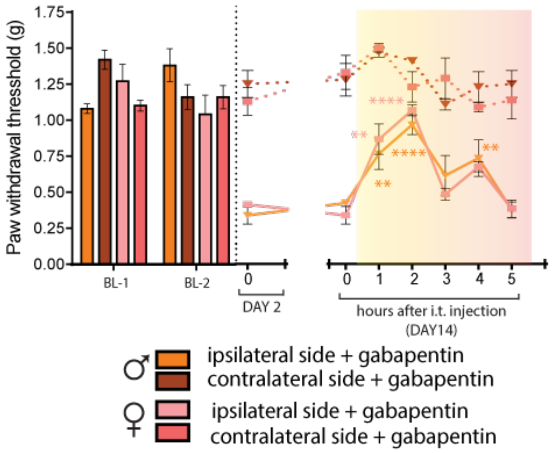

Appendix Figure S14. Gabapentin i.p. administration in chronic phase SNI animals.

Von Frey test in chronic phase (day 14 after surgery) shows full recovery from mechanical hypersensitivity at 2 h following i.p. administration of 30 mg/kg gabapentin in both genders SNI animals.

Data information: Both groups contain n = 4 pr. group pr. gender. All datapoints are expressed as mean with error-bars as SEM. Ipsi- and contralateral paw withdrawal thresholds at different time points were compared to time 0 using two-way repeated measures ANOVA followed by Dunnett's multiple comparisons test (***P*<0.01, *****P*<0.0001).

Appendix tables and legends

Appendix Table S1. N-terminal modifcation to increase affinity of DAT C3, C4 or C5 peptides

**Starting peptide**	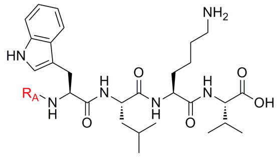 4 AA	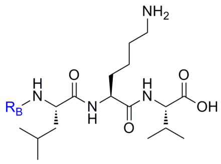 3 AA	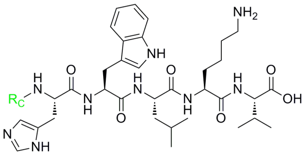 5 AA
	R_A K_i (µM) [SEM interval]	R_B K_i (µM) [SEM interval]	R_c K_i (µM) [SEM interval]
**No modification**	5.69 [5.35;6.05]	4.10 [3.78;4.44]	1.16 [0.93;1.46]
**N-alkylation (R)**			
**CH_3**	18.75 [17.55; 20.04]	9.38 [8.73; 10.07]	2.52 [2.34; 2.70]
**CH_2CH_3**	30.01 [28.09; 32.06]	N.D	N.D.
**$(CH_2)_2CH_3$**	46.64 [42.54; 51.15]	N.D	N.D.
**$(CH_2)_3CH_3$**	45.27 [41.78; 49.06]	N.D	N.D.
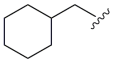	15.06 [14.48;15.66]	6.82 [6.67; 6.97]	2.87 [2.73; 3.03]
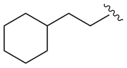	31.84 [25.08; 40.43]	N.D	N.D.
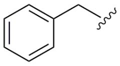	24.52 [20.27; 29.66]	N.D	N.D.
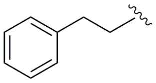	26.25 [21.92; 31.44]	N.D	N.D.
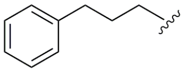	38.55 [37.78; 39.34]	N.D	N.D.
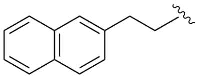	48.03 [38.33; 60.19]	N.D	N.D.
**N-acetylation (R)**			
**$COCH_3$**	3.49 [3.35; 3.64]	3.80 [3.69; 3.92]	1.48 [1.40; 1.57]

Appendix Table S2. Peptide affinities for purified PICK1 determined by FP. At 0.01% Triton-X 100 PICK1 is a dimer. At 0.1% Triton-X 100 PICK1 is a monomer.

Tracer identity*	Tested peptide	Triton-X 100 concentration	K_D** or K_i***	[SEM] interval
OrG-C11 (DAT)	OrG-C11	0.01 %	0.44 µM	[0.35;0.54] µM
	C5	0.1 %	9.10 µM	[6.0;13.8] µM
		0.01 %	1.90 µM	[1.6;2.2] µM
	Tat-C5	0.1 %	113 nM	[106;120] nM
		0.01 %	106 nM	[86.5;129] nM
	Tat-P_4-$(C5)_2$	0.1 %	30.2 nM	[25.4;35.9] nM
		0.01 %	39.3 nM	[36.0;43.0] nM
OrG-GluA2 C11	Org-GluA2 C11	0.01 %	1.53 µM	[1.09;2.14] µM
	C5	0.01 %	3.4 µM	[2.88;4.0] µM
	Tat-C5	0.01 %	216.7 nM	[206.4;227.6] nM
	Tat-P_4-$(C5)_2$	0.01 %	75.8 nM	[71.4;80.4] nM
5FAM-Tat-C5	5FAM-Tat-C5	0.01 %	94.8 nM	[80.5;111.8] nM
	C5	0.1 %	471.8 nM	[296.0;752.0] nM
		0.01 %	1.7 µM	[1.3;2.2] µM
	Tat-C5	0.1 %	44.4 nM	[33.3;59.2] nM
		0.01 %	18.3 nM	[15.9;21.0] nM
	Tat-P_4-$(C5)_2$	0.1 %	28.9 nM	[19.5;42.9] nM
		0.01 %	6.2 nM	[5.53;7.0] nM
5FAM-Tat-C5 (2 nM)	5FAM-Tat-C5	0.01 %	19.5 nM	[18.6;20.3] nM
	Tat-C5	0.01 %	13.30 nM	[10.6;16.5] nM
	Tat-P_4-$(C5)_2$	0.01 %	1.7 nM	[1.1;2.7] nM

Notes:
* Tracer concentration is 20 nM unless stated otherwise
** K_D is obtained from saturation curves fitted to a log dose response curve using GraphPad Prism 8.3
*** K_i is obtained form competetion curves fitted to a one-site competetion curve using GraphPad Prism 8.3

Appendix Table S3. SAXS data table

	PICK1 Apo							PICK1, Tat-P_4-$(C5)_2$				
**Sample details**												
Uniprot ID			Q9EP80									
Organism			Rat									
Ligands	-							Tat-P_4-$(C5)_2$				
Buffer	50mM Tris, 125mM NaCl, 2mM DTT, 0.01% Triton-X100, pH 7.4							50mM Tris, 125mM NaCl, 2mM DTT, 0.01% Triton-X100, pH 7.4				
Extinction coefficient	32320*[1]							35443*[2]				
Molecular weight	46.6 kDa *[1]							47.5 kDa*[2]				
Protein concentration	0.5-3 mg/ml*[3]							0.5-2.45 mg/ml*[3]				
	**SAXS data collection details**											
Instrument	P12, Petra III, DESY											
Date for data collection			10-06-2015									
Wavelength			1.24									
Beam intensity			12.16									
Measured *q*-range			0.0027-0.48									
Absolute calibration			water									
Exposure time			20 ms									
Temperature			283.35									
	**Software**											
Indirect Fourier transformations to obtain p(*r*)			Bayersapp.org									
Fitting of data with combined analytical and atomic models			ATSAS, EOM, Ranch, Gajoe									
Data rebin			WillItRebin*[4]									
	**Structural parameters**											
**P(r) analysis**	PICK1 WT						PICK1 + Tat-P_4-$(C5)_2$					
Sample concentration [mg/ml]	0.5	0.88	1.5	2.0	2.5	3.0	0.5	0.75	1	1.5	2.0	2.45
I0 estimate	0.15	0.19	0.17	0.24	0.27	0.30	0.13	0.13	0.13	0.13	0.14	0.14
Rg [Å]	114	134	132	147	148	163	69	77	69	75	75	80
Dmax [Å]	368	453	442	507	506	567	237	307	235	309	289	308
q-range	0.0075-0.3918											
Reduced χ^2	0.86	0.94	0.61	0.55	1.03	0.55	0.99	0.66	0.87	0.73	0.85	0.69
Number of good parameters	13.95	21.85	33.36	29.07	31.72	39.28	9.11	13.78	13.85	18.45	18.96	21.68
Number of Shannon channels	44.95	55.35	54.10	62.00	61.84	69.34	29.02	37.54	28.84	37.78	35.35	21.68
Number of	103	86	109	106	88	115	45	97	103	94	87	96

error calculations												
Regularization parameter log(α)	13.97	13.45	13.36	13.30	13.17	12.47	14.47	13.64	13.77	13.34	13.37	13.10
Mw determination												
Molecular weight (kDa)	210	262	233	336	373	420	183	186	177	184	197	219
Expected ratio	4.4	5.5	4.9	7.1	7.9	8.8	3.8	3.9	3.7	3.9	4.1	4.6
	**Model fitting parameters**											
Combined analytical and atomistic model												
q-range	Not fitted							0.015-0.269				
Reduced χ^2 (best fit)	Not fitted							0.8				
Ensemble optimization method (EOM)												
Ranch pool generation								10.000 / pool<br>15 Harmonics<br>0-10% Symmetric structures				
Gajoe selection and fitting								1000 generations<br>100 ensembles<br>max 20 curves ensemble<br>100 repetitions				
	**SASBDB IDs for data and models**											
SASDB ID	TBA							TBA				
	**Footnotes and references**											
	[1] Calculated using Expasy Protparam (https://web.expasy.org/protparam/)<br>[2] as *1 but assuming ½ peptide pr. PICK1 molecule<br>*[3] Protein concentration was determined using UV280 absorption<br>*[4] Binfactor 1.02, absolute scaling factor (water) for beamtime 0.00000234.											
