# Supplementary material for: A high‐affinity, bivalent PDZ domain inhibitor complexes PICK1 to alleviate neuropathic pain
Source: EMBO Mol Med. 2020 Apr 30;12(6):e11248. doi: 10.15252/emmm.201911248 (PMC7278562; doi:10.15252/emmm.201911248)
Supplement: Supplementary file 2 — Expanded View Figures PDF [file EMMM-12-e11248-s002.pdf]

Expanded View Figures

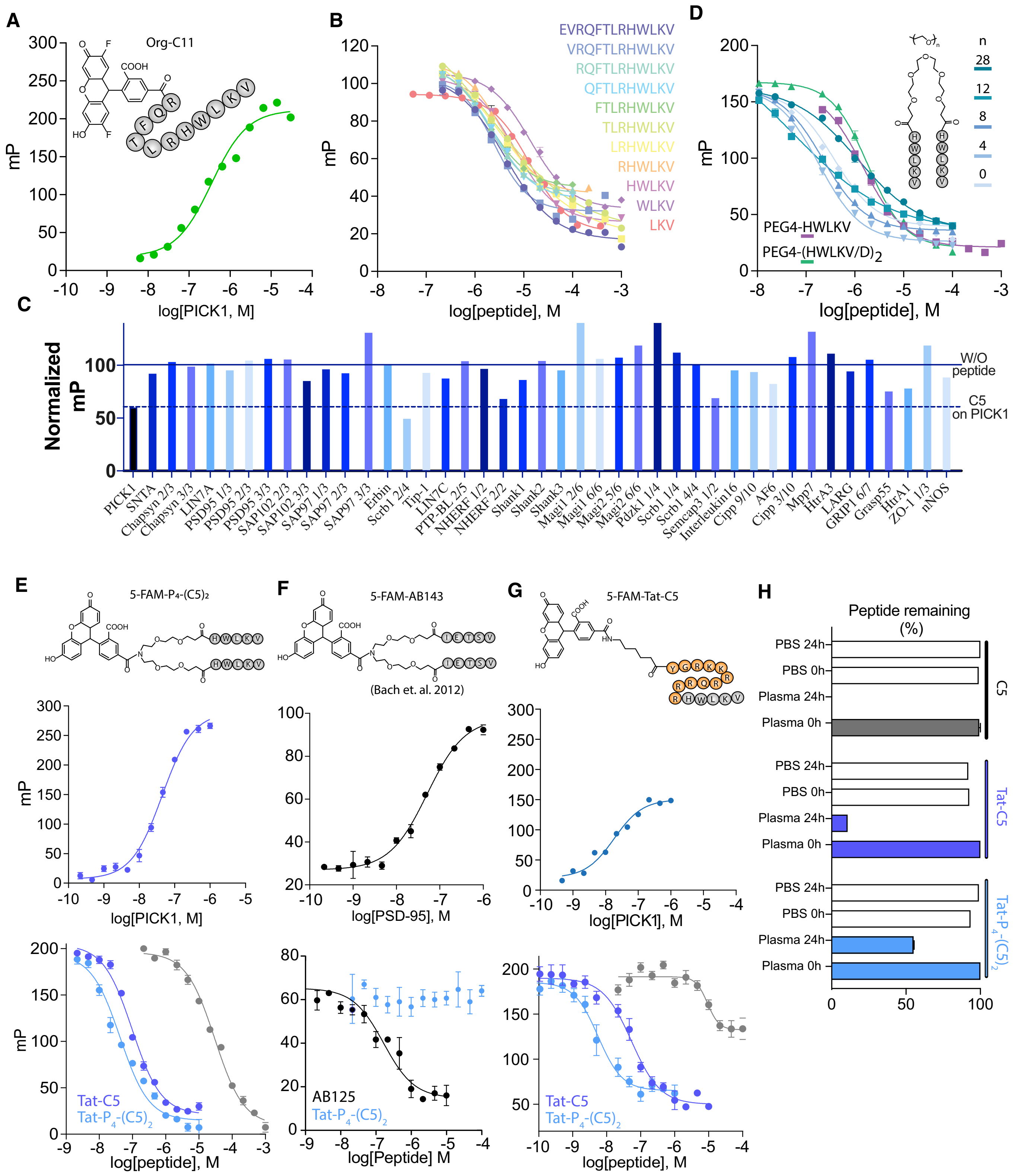

Figure EV1.

 © 2020 The Authors

Figure EV1. Development of Tat-C5 and Tat-P_4-$(C5)_2$.

A Fluorescence polarization saturation binding using Org-C11 (20 nM) against an increasing concentration of PICK1.
B Effects of truncation on competitive binding of DAT C-terminal peptides to PICK1 using Org-C11 (20 nM) as reporter.
C Selectivity screen for Tat-C5 against a selection of 42 purified PDZ domains, performed with a fixed concentration of C5 (10 μM) in competition with PDZ domains and their respective fluorescent ligands. Data are normalized to binding in absence of peptide (full line). Dashed line represents the level of competition obtained for PICK1.
D Competitive binding curves of bivalent C5 PEG_n variants. With PEG linker, length indicated.
E Saturation (top) and competition binding curves using 5-FAM-PEG_4-$(C5)_2$ (5 nM) and PICK1 against C5, Tat-C5, and Tat-P_4-$(C5)_2$.
F Saturation (top) and competition binding curves using 5-FAM-AB143 (5 nM) and PSD-95 against AB125 (positive control), and Tat-P_4-$(C5)_2$.
G Saturation (top) and competition binding curves using 5-FAM-Tat-C5 (2 nM) and PICK1 against C5, TatC5, and Tat-P_4-$(C5)_2$.
H Plasma stability as assessed by incubation of peptides as indicated with human plasma for 24 h followed by quantification by UPLC.

Data information: Data are shown as mean with error bars as SEM of $n \geq 3$. Binding curves were fitted to a log dose response (three parameters) extracting the K_d or IC_{50} using GraphPad Prism 8.3. K_i was calculated using the Cheng–Prusoff equation.

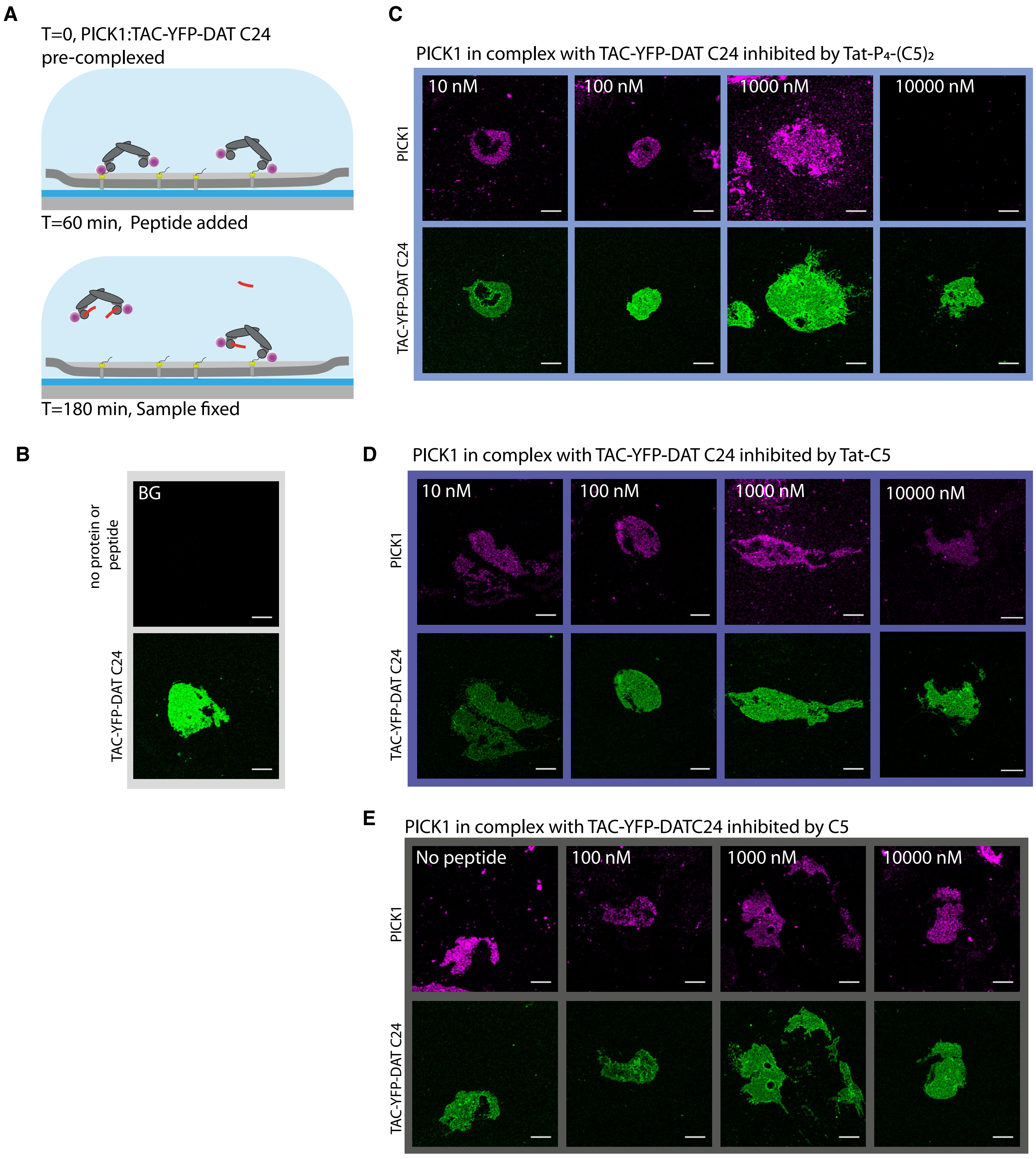

Figure EV2.

 © 2020 The Authors

Figure EV2. Dissociation of PICK1 binding from SCMSs in presence of inhibitory peptides.

A Illustration of experimental approach. Fluorescently labeled PICK1 was allowed to interact with the supported cell membrane sheet (SCMS) expressing TAC-YFP-DAT C24 prior to incubation with peptide for 1 h.

B–E Representative fluorescence confocal images of SCMS expressing TAC-YFP-DAT C24 (green) incubated with fluorescently labeled PICK1 (magenta) and subsequently incubated with unlabeled peptide at indicated concentrations. Visible reduction in binding of PICK1 (magenta) is observed at 10 μM of Tat-P_4-$(C5)_2$, but not for Tat-C5 and C5, suggesting that Tat-P_4-$(C5)_2$ can facilitate dissociation of PICK1 from SCMSs. For curves in Fig 1F, images were pooled from three independent experiments. Note that images with 10,000 nM peptide are the same as used in Fig 2E.

Data information: In all images, scale bars represent 10 μm.

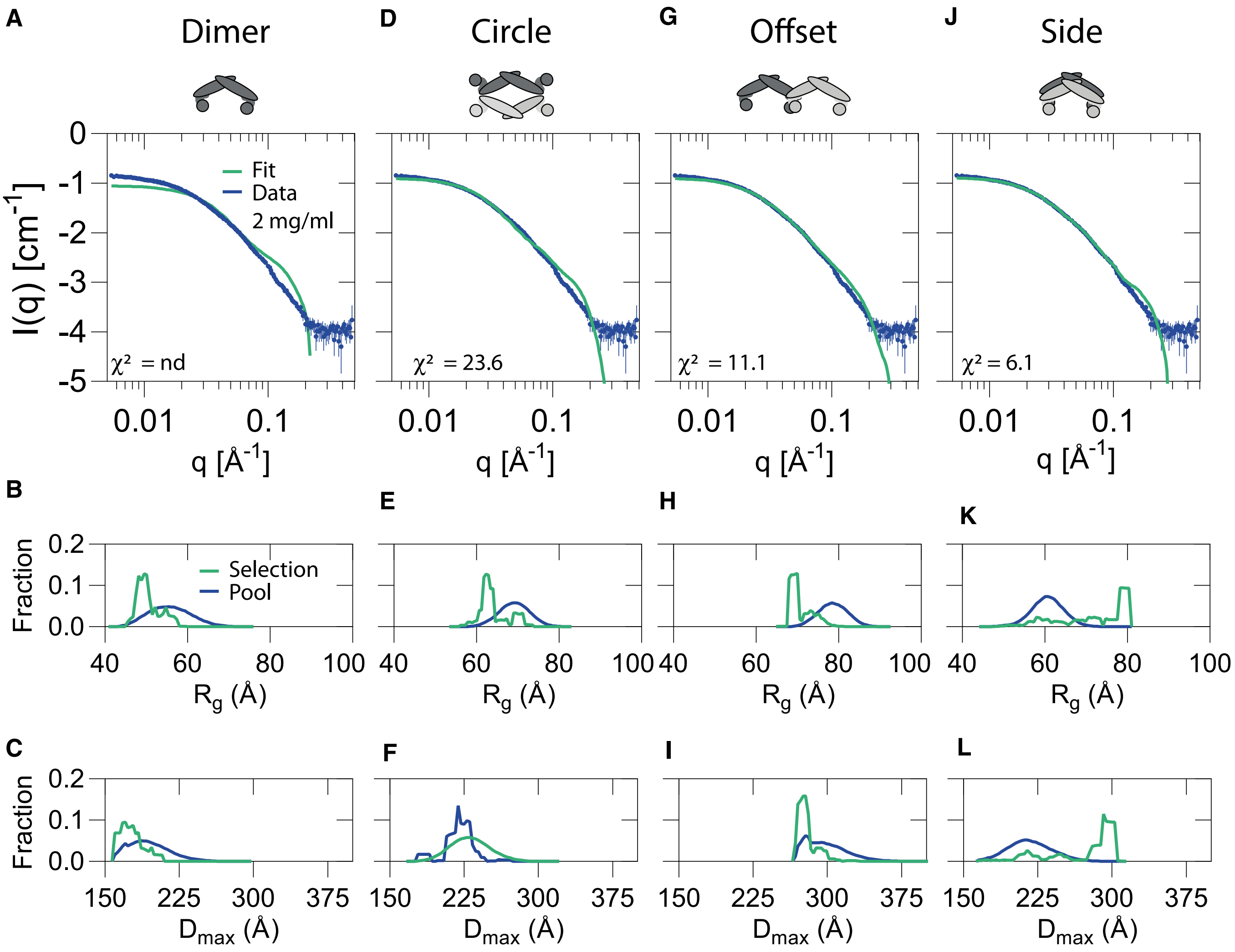

Figure EV3. Ensemble optimization method fit of putative tertiary PICK1 configurations to the SAXS data.

A–L Ensemble optimization method (EOM) fit (green) to scattering data from Fig 3H of PICK1 in complex with Tat-P_4-$(C5)_2$ (2 mg/ml) (blue) using a pool (10,000 structures) with R_g and D_{max} distribution shown below for (A-C) dimeric PICK1, (D-F) a circularly configuration of tetrameric PICK1, (G-I) an offset configuration of tetrameric PICK1, and (J-L) a side-by-side circularly configuration of tetrameric PICK1. The side-by-side configuration provided the best fit as indicated by the lowest χ^2-value.

© 2020 The Authors

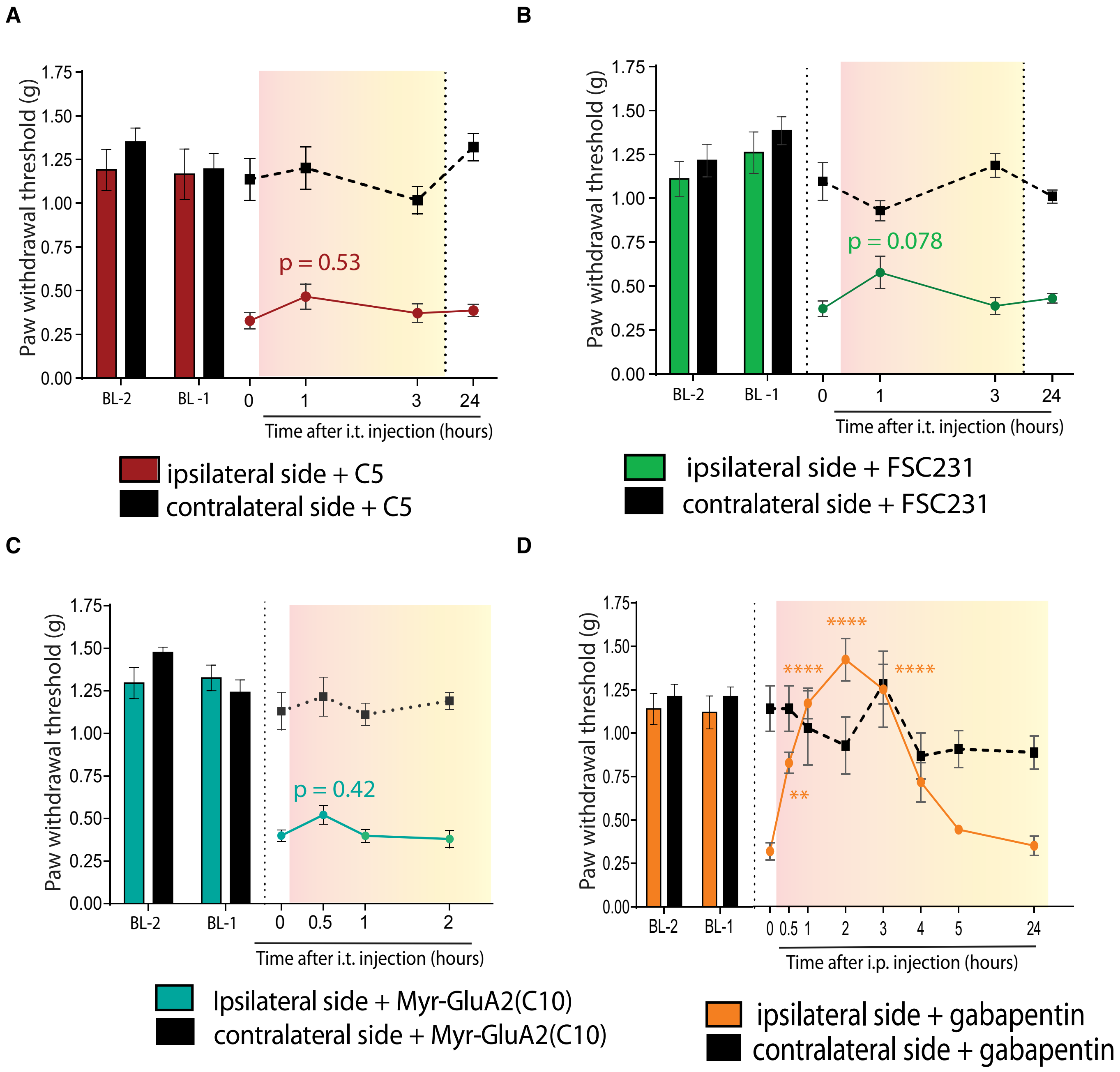

Figure EV4. Other PICK1 inhibitors fail to attenuate mechanical hyperalgesia but gabapentin does.

A i.t. administration (20 μM) of C5 did not significantly change PWT in the initiation stage (2 days after injury) of SNI-induced hypersensitivity

B i.t. administration (20 μM) of the small-molecule PICK1 inhibitor FSC231 did not significantly change PWT in the initiation stage (2 days after injury) of SNI-induced hypersensitivity

C i.t. administration (20 μM) of myrGluA2 did not significantly change PWT in the initiation stage (2 days after injury) of SNI-induced hypersensitivity

D i.p. administration of gabapentin (30 mg/kg) produced full recovery of ipsilateral paw threshold in the initiation stage (2 days after injury) of SNI-induced hypersensitivity serving as reference compound in acute phase SNI animals at time 1, 2, 3 h.

Data information: Data on graphs are mean ± SEM. Ipsi- and contralateral paw withdrawal thresholds at different time points were compared to time 0 using two-way ANOVA followed by Dunnett's multiple comparison test (**$P < 0.01$, ****$P < 0.0001$). (A-C) $n = 8$ mice/group, (D) $n = 4$ mice.

© 2020 The Authors

Figure EV5. Illustration of proposed model for the ability of Tat-P_4-$(C5)_2$ to increase macroscopic off-rate of PICK1 bound to receptors embedded in the membrane.

PICK1 binds to membrane-embedded receptors, such as the AMPARs (orange), in two distinct states, a bivalent and a multivalent bound conformation, mainly driven by lipid binding motifs (black dashed circles) positioned in the PDZ domain and the amphipathic BAR-PDZ linker (Erlendsson *et al*, 2019). Upon addition of Tat-P_4-$(C5)_2$, two or more PDZ domains of the PICK1 tetramer and the lipid binding motifs gets constrained, thereby favoring an upright position of PICK1, with a maximum of two PDZ domains and lipid binding regions of the PICK1 tetramer binding to the membrane and membrane-embedded receptors. Addition of Tat-C5 or C5 does not constrain the PDZ domains, which are then free to exchange between the bivalent upright PICK1 and the multivalent membrane parallel bound PICK1, thereby not affecting the macroscopic off-rate. The model was created using PyMoL 2.0. The tetrameric PICK1 model was generated in earlier EOM modeling, and the position of the model is based on earlier work (Erlendsson *et al*, 2019). The structure of AMPARs (PDB: 5VHW) was positioned into a POPE lipid bilayer manually, and a *de novo* model of the GluA2 cytoplasmic tail (UniProt: P42262, residues 834-883) was generated using PEP-FOLD 3 (Thevenet *et al*, 2012; Shen *et al*, 2014; Lamiable *et al*, 2016) and orientated to the C-terminal helix of the AMPAR structure manually.

© 2020 The Authors

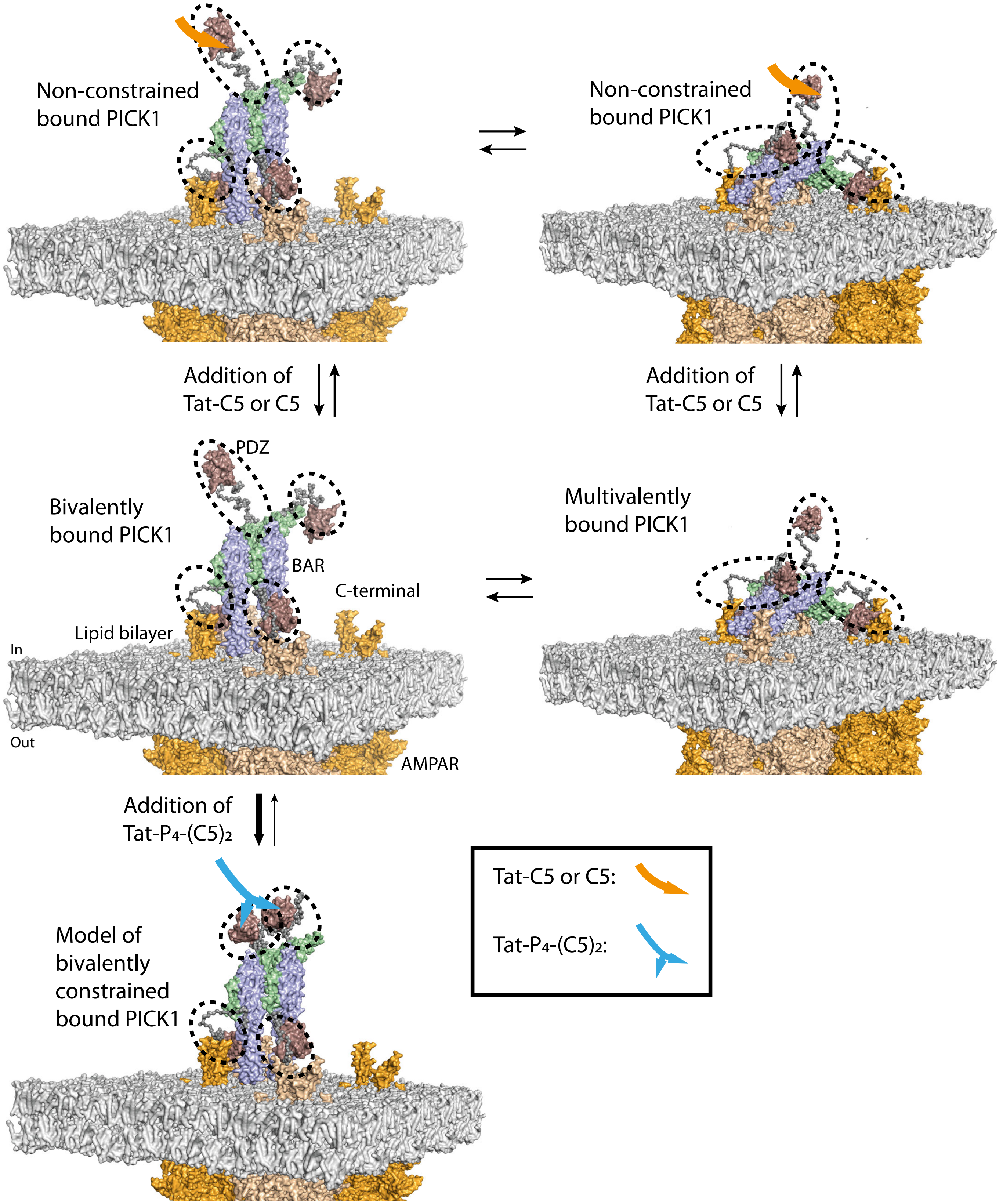

Figure EV5.

© 2020 The Authors
