# Supplementary figures and images for: A high‐affinity, bivalent PDZ domain inhibitor complexes PICK1 to alleviate neuropathic pain
Source: EMBO Mol Med. 2020 Apr 30;12(6):e11248. doi: 10.15252/emmm.201911248 (PMC7278562; doi:10.15252/emmm.201911248)

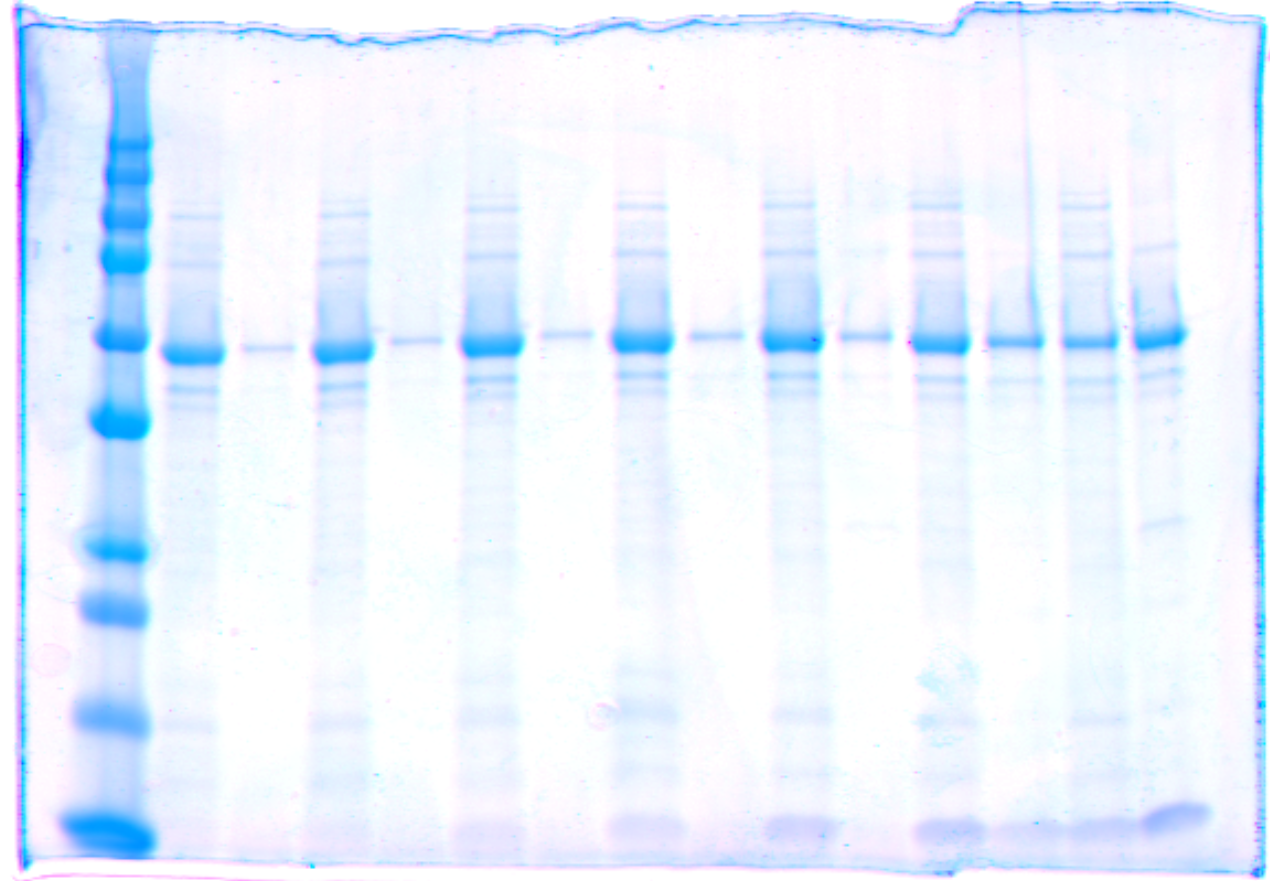

Supplement: Supplementary file 4 — Source Data for Figure 3 [file EMMM-12-e11248-s003.tif]
